# Identification of novel genes associated with longevity in *Drosophila melanogaster* - a computational approach

**DOI:** 10.18632/aging.102527

**Published:** 2019-12-03

**Authors:** Bethany S. Hall, Yvonne A. Barnett, Jonathan J. Crofts, Nadia Chuzhanova

**Affiliations:** 1 School of Science and Technology, Nottingham Trent University, Nottingham NG11 8NS, UK; 2 Current address: Faculty of Science and Engineering, Anglia Ruskin University, Cambridge, Cambridgeshire CB1 1PT, UK

**Keywords:** single nucleotide polymorphisms, longevity, *Drosophila*, networks, target genes

## Abstract

Despite a growing number of studies on longevity in *Drosophila*, genetic factors influencing lifespan are still poorly understood. In this paper we propose a conceptually new approach for the identification of novel longevity-associated genes and potential target genes for SNPs in non-coding regions by utilizing the knowledge of co-location of various loci, governed by the three-dimensional architecture of the *Drosophila* genome. Firstly, we created networks between genes/genomic regions harboring SNPs deemed to be significant in two longevity GWAS summary statistics datasets using intra- and inter-chromosomal interaction frequencies (Hi-C data) as a measure of co-location. These networks were further extended to include regions strongly interacting with previously selected regions. Using various network measures, literature search and additional bioinformatics resources, we investigated the plausibility of genes found to have genuine association with longevity. Several of the newly identified genes were common between the two GWAS datasets and these possessed human orthologs. We also found that the proportion of non-coding SNPs in borders between topologically associated domains is significantly higher than expected by chance. Assuming co-location, we investigated potential target genes for non-coding SNPs. This approach therefore offers a stepping stone to identification of novel genes and SNP targets linked to human longevity.

## INTRODUCTION

Despite a growing number of studies on survival into old (≥ 85 years) and advanced (≥ 90 years) age, factors influencing longevity (or lifespan) are still poorly understood. Human twin studies estimated that 20–30% of variation in survival into old and advanced age, besides maintaining a healthy life style, is determined by heritable genetic factors [1, 2].

In order to determine these genetic factors, several genome-wide scans for linkage, genome-wide association studies (GWAS) and genome-wide association meta-analyses have been carried out on panels of long-lived individuals. Variations in many loci, e.g. near the *D4S1564* [3], *MINPP1* [4], *HLA-DQA1/DRB1* and *LPA* [5] genes, have been identified as contributing to survival into old age, but only single nucleotide polymorphisms (SNPs) in *TOMM40*/*APOE* and *FOXO3* loci were found to robustly associate with longevity [6–11]. In a whole-genome scan for genetic linkage performed by Kerber et al. [12] on individuals from the Utah Population Database, in which high levels of both familial longevity and individual longevity were exhibited, the strongest signal was observed in marker D3S3547 on chromosome 3p24.1. In addition, a locus on chromosome 3p24-22, previously identified in [13], was found to link to exceptional longevity [12], strengthening the case that genes found in these regions play a role in the regulation of human lifespan. Boyden and Kunkel [13] have identified several additional loci as having significant association with longevity, e.g. on chromosomes 9q31-34, 12q24 and 4q22-25. Recently, GWAS of parental longevity was performed on participants of European descent available via the UK Biobank [14]. Several previously known variants have been confirmed in this study. In addition, other common variants previously found by disease-specific GWAS to associate with e.g. cellular senescence, inflammation, lipid metabolism and cardiovascular conditions were also found to associate with parental longevity [14]. Their results suggest that human longevity is a highly polygenic trait influenced by many variants with a small effect size [14].

Progress in studies of human longevity is being exacerbated by small sample sizes making model organisms, such as *Drosophila melanogaster*, increasingly important for studying and understanding genetic factors affecting longevity. The lifespan of *Drosophila* is affected by several factors including genetics, differences in environmental conditions, diet and overcrowding. In laboratory conditions under controlled environment the average lifespan is found to be 26 and 33 days for female and male *Drosophila*, respectively [15]. Mutations in several genes have been found to increase the lifespan of *Drosophila*. For example, a mutation in the *mth* (Methuselah) G protein-coupled receptor gene, which leads to the partial loss-of-function, has been found to extend the average lifespan by 35% [16]. Mutant versions of the *Indy* gene, which encodes an amino acid transporter, has been shown to double the average lifespan [17]. It was also shown that single gene mutations in the target of rapamycin (TOR) and the insulin/insulin-like growth factor (IIS) signaling pathways can slow down the aging process in model organisms including flies [18].

Up to date, *Drosophila* GWAS have identified millions of naturally occurring SNPs that potentially influence longevity. Burke et al. [19] compared allele frequencies in the oldest surviving *Drosophila* with the randomly selected individuals from the same “synthetic” populations, derived from eight inbred founders. Eight significantly divergent regions have been identified. A small proportion of genes, found in these regions, were enriched in Genome Ontology (GO) biological process terms ‘defense response’ and ‘glutathione metabolic process’ [19]. Ivanov et al. [20] used lines from the *Drosophila melanogaster* Genetic Reference Panel (DGRP) to perform GWAS and identified ~2 M common SNPs. However, none of the SNPs found reached genome-wide significance level prompting the hypothesis of a possible combined effect of common SNPs on longevity. Gene-based analysis with either gene regions or gene regions extended into ±5 Kb of flanking sequences had identified several top-ranked genes including the *CG11523* and *Neprilysin 1*. The former was found to have a *GSK3β* interaction domain that is a crucial component of the TOR pathway in human cell lines [20]; the latter could be essential for female fitness [20]. Among the top-ranked 100 genes (p < 4.79×10^-6^) found in this study were *Chrb, slif, mipp2, dredd, RpS9* and *dm* genes enriched in the ’TOR pathway’ GO term [20]. Several of the longevity associated genes found are involved in processes which are known to impact aging (e.g. carbohydrate metabolism), however the function of others (although not known) provided opportunity for further, promising experimental examination. Polygenic score analysis was also used to find the additive effects of common SNPs [20]. In the absence of the second dataset, cross validation was performed. It was found that a small proportion of the observed lifespan variation (~4.7%) is explained by the additive effect of common SNPs. Despite the success in identification of variants, associated with longevity, the functional role of the majority of them – especially the variants residing outside the gene coding regions – remains to be determined.

In this paper we hypothesize that co-location of known longevity-associated genes with genes, not previously implicated in longevity, and their enrichment in the same biological function or pathway as known genes, make them novel candidate genes, potentially linked to longevity. We further hypothesize that both non-coding SNPs and their potential target genes also reside within co-located loci. To identify these novel genes/genomic regions we devised a computational approach based on analysis of networks of co-located loci, harboring both GWAS-identified variants and novel genes. Two datasets of SNPs generated by GWA studies [19–20] were used, comprising respectively ~1 million and ~2 million SNPs and sharing 2139 SNPs residing within 1515 (possibly overlapping) genes and 1044 non-coding SNPs.

As a measure of co-location (or proximity) of two distinct loci, not necessarily on the same chromosome, we used inter- and intra-chromosomal contacts generated by chromosome conformation capture Hi-C technique for the *Drosophila melanogaster* genome [21]. Studies of chromosome conformations have revealed that three-dimensional architecture of chromatin dictates the co-location of specific genes within the nucleus, thereby prompting the hypothesis of existence of common mechanisms controlling their transcription in a tissue-specific manner [22–23]. Recently, Won et al. [24] have demonstrated the advantages of using 3D chromatin maps for identifying target genes for schizophrenia-associated SNPs, residing within non-coding reasons of the genome. The findings have shown that for many non-coding SNPs their target genes were neither adjacent to SNPs nor in linkage disequilibrium, proving the point that many regulatory interactions are not captured by linear chromosomal organization. Analysis of intra-chromosomal interactions showed more frequent and stronger interactions within continuous genomic regions, called topologically associated domains (TADs), than with regions residing in other TADs [22–23]. TADs have been proven to play important roles in 3D organization of genomes and gene regulation and, when mutated, may lead to disease through disruption of gene regulatory pattern (reviewed in [25]).

A network of interactions was created from the inter- and intra-chromosomal contacts with nodes representing genomic regions, connected by edges, weighted by interaction frequencies. We calculated various network measures (e.g. degree [26]) and identified communities (i.e. densely connected subnetworks) existing within the network with the aim of revealing influential nodes/regions and densely connected communities (clusters) within networks. Candidate regions and communities were further explored using FlyBase (http://flybase.org/) and FlyMine (http://www.flymine.org/) resources, and GeneAge database (http://genomics.senescence.info/genes/models.html) to provide a body of evidence for genomic regions having genuine and/or previously unknown association with longevity.

To explore the role that SNPs occurring in TAD borders play in longevity, we analyzed genes residing in close proximity to TAD borders and sharing both ‘long-lived’ and ‘short-lived’ phenotypes. We hypothesized that a SNP(s) in nearby TAD borders may lead to a disruption of a regulatory pattern of a gene resulting in one of the phenotypes, ‘long-lived’ or ‘short-lived’, whereas the opposite phenotype could be a consequence of SNPs residing within genes themselves.

## Results and Discussion

### Choice of interaction frequency thresholds and genome-wide significance level

To assess the strength of interactions between intra- and inter-chromosomal genomic regions, distributions of interacting frequencies were analyzed individually for each chromosome and between chromosomes. Only 1% of the strongest intra-chromosomal interactions corresponding to the tails of these distributions and resulting in frequencies greater than 247, 215, 1308 and 342 for chromosomes 2, 3, 4 and X, respectively, were considered. The threshold for inter-chromosomal interaction frequencies, corresponding to 1% of strongest interactions, was 10. We refer to interactions with frequencies exceeding these thresholds as “strong” interactions.

The genome-wide significance level, required for finding association between ~10^6^ SNPs, is usually set to p < 5×10^-8^. This value corresponds to 0.05 level of significance after correction for multiple testing. In our case, each SNP was binned into a 80 Kb region. There are 1503 distinct 80 Kb regions recorded in the *Drosophila* Hi-C data. Taking this into account, we corrected the required significance level to 3.33×10^-5^. In the analysis of SNPs in non-coding regions the Hi-C data with finer resolution, 10 Kb, was used where interaction frequencies between 11,839 10 Kb bins were available [21]; in this case the genome-wide level of significance was set to 0.05/11839=4.22×10^-6^. Following [19], SNPs with *D*-values exceeding 7.9 were deemed to be significant.

### Original networks of interaction based on Synthetic and DGRP GWAS data

The original network of interaction based on the Synthetic GWAS data consists of 279 nodes each representing a 80 Kb region harboring at least one SNP with *D* > 7.9. In turn, the original network of interaction based on the DGRP GWAS data consists of 80 nodes corresponding to regions harboring SNPs with p-values < 3.33×10^-5^. The original networks share 14 common nodes covering 1.12 Mb of the *Drosophila* genome and harboring 168 genes. Only five genes ‒ *Rim2* (replication in mitochondria 2), *GlyP* (glycogen phosphorylase), *aop* (anterior open), *HDAC1* (histone deacetylase 1) and *Tpi* (triose phosphate isomerase) ‒ were found in FlyBase database as having “long-lived” phenotype. The number of SNPs residing within these common regions and satisfying chosen thresholds was 91 and 19 for Synthetic and DGRP GWAS-based data, respectively. Among the genes with the highest number of SNPs recorded in both GWAS datasets were *nmo*, *sima*, *axo*, *CG9967*, *eys*, *chinmo* and *dpr3* (for the full list of genes see Supplementary Table 1).

### Extended networks of interactions

Original networks were further expanded to create extended networks by adding extra nodes, corresponding to 80 Kb fragments that interact with frequencies meeting interaction frequency thresholds with the nodes, already present in the original networks. Together with regions that harbor SNPs recorded in the corresponding GWAS datasets, the extended networks contain novel regions that may not be covered by techniques used for SNP identification. We refer to these networks as Synthetic and DGRP GWAS-based (extended) networks.

The Synthetic GWAS-based extended network is fully connected and consists of 1099 nodes harboring ~75% (69,951) of SNPs recorded in the Synthetic GWAS dataset with 2,409 SNPs residing within genes. Among 13,838 genes residing within the network nodes 217 genes were found to have “long-lived” phenotype as recorded in the FlyBase database. The node labelled 547 (corresponding to region Chr2R: 20800000-20880000) has the highest degree, 150.

The DGRP GWAS-based extended network has six disconnected components and consists of 671 nodes harboring ~50% (1,093,533) of SNPs recorded in the DGRP GWAS dataset with 114 SNPs residing within genes. Among 8,929 genes residing within the network nodes 145 genes were found to have “long-lived” phenotype according to the FlyBase database. The node labelled 1183 (region Chr3R: 25920000-26000000) has the highest degree of 68.

The extended networks share 527 common nodes covering 42.16 Mb of the *Drosophila* genome and harboring 7,413 genes among which 121 have “long-lived” phenotype. Fifteen common regions do not harbor any genes. For approximately 30% and 3% of genes residing within common regions no SNPs were recorded in the Synthetic and DGRP GWAS datasets, respectively. Among the genes with the highest number of SNPs recorded in both GWAS datasets were *Ptp61F*, *CG45186*, *kirre*, *Ptp99A* and *CG44153*. Only a small proportion of genes found in regions common for both datasets were harboring SNPs meeting our significance threshold – 717 and 57 in the Synthetic and DGRP GWAS-based networks, respectively.

Several novel regions with the highest degree were selected for further analysis and each of the subnetworks centered around these novel regions (i.e. together with all connected regions) were considered (Supplementary Table 2). Genes residing within these subnetworks were sought for enrichment in longevity-associated GO terms. The results are summarized in Table 1.

**Table 1:** Novel nodes with the highest degree in the Synthetic and DGRP GWAS-based networks harboring genes enriched in longevity-associated GO terms.

**Novel node**	**Network**	**GO term**	**P-value**	**Genes enriched in GO term**	**Number of nodes harboring genes enriched in GO term/ total number of nodes**
928	Synthetic	Apoptotic process	2.27E-04	*E2f2*, lola*, *egr, Ret, Vps25, TER94, ptc, eEF5*(*CG3186*)*, snama, ninaA, yki, sigmar, l(2)tid, Mcm10*	7/16
928	Synthetic	Nervous system development	4.37E-04	*CG10339, amos, CG10431*, Sidpn**, RpL30, hook, Dap160, *enok, lola, dgo, egr*, ***CG12935***, *Ret, Pka-R2, Eps-15, Galphao*	9/16
1220	Synthetic	DNA repair	0.0294	***Top3alpha**, PCNA2*(*CG10262*), *Nipped-B, CG9272, **RPA2***	4/21
28	DGRP	immune system process	0.021515	*Vps16B, Cad99C, **aop**, DPCoAC*(*CG4241*)*, **Stat92E**, Mtl, **GlyP***	4/8
2	DGRP	cellular response to stress	0.006104	*CG11498, **Clbn**, CG13473, CG14130, Sld5, mu2, **Atg16**, **kay**, CG3448, Rad9, Mtl, Grx1*(*CG6852*)*, **Cat**, **HipHop**, **BI-1**, Wdr24*(*CG7609*)*, Drice*	13/15

* Genes residing within original nodes, i.e. harboring SNPs with D > 7.9 are underlined.

**Genes previously found to have association with longevity as recorded in FlyBase or GeneAge resources are shown in bold.

Genes residing within a subnetwork centered around node 928 (chr3R:5520000-5600000) in the extended Synthetic GWAS-based network were enriched in two GO terms, ‘apoptotic process’ and ‘nervous system development’ (Table 1). Among them the *trbd* and *CG8412* genes that have ‘short-lived’ phenotype according to in FlyBase resources. The loss of the *trbd* gene, a negative regulator of the *Drosophila* immune-deficiency pathway, has previously been observed to reduce lifespan [27]. A number of genes in this subnetwork, including *dmt*, *hyd*, *CG16908* and *CG9471*, were found to have phenotypes ‘increased mortality’ and ‘lethal’. The *MED6* gene was found to have a phenotype of ‘cell lethal’ and is known to be required for elevated expression of a distinct set of developmentally regulated genes. This gene is essential for viability and/or proliferation of most cells and mutants of this gene have previously been observed to fail to pupate, dying in the third larval instar with severe proliferation defects in imaginal discs and other larval mitotic cells [28]. Finally, this subnetwork also contains the *FoxP* gene, a protein that encodes a transcription factor expressed in the nervous system. This gene has recently been shown to be important for regulating several neurodevelopmental processes and behaviors that are also related to human disease [29].

Many of the newly found genes (see Table 1) share the same biological function and co-locate with genes that have previously been reported to associate with longevity and/or aging, thus acting as a proof of concept. For example, the *sidpn*, *hook* and *CG12935* genes residing in subnetwork centered around bin 928 (chr3R:5520000-5600000) were reported to have a ‘short-lived’ phenotype. Loss-of-function mutation in the *hook* gene has been found to reduce maximum lifespan by up to 30% [30]. Mutant flies lacking mitochondrial *Top3alpha* gene have also been found to have decreased maximum lifespan by up to 25%, in which a premature aging phenotype was demonstrated and mobility defects were observed [31]. Several genes, e.g. *RpL30*, *Eps-15*, *Nipped-B* and *RPA2*, listed in Table 1 were also found to have an ‘increased mortality’ phenotype according to the FlyBase resources.

Five genes residing in a subnetwork centered around bin 1220, were enriched in the ‘DNA repair’ GO term. Interestingly, this novel region is located on chr4: 960000-1040000, a chromosome seen as an anomaly because of its small size in comparison to other chromosomes and its chromatin structure. Due to its size, this chromosome is often ignored, however it is known to harbor at least 16 genes where many of them are thought to have male-related functions [32]. Using a comprehensive database of *Drosophila* regulatory sequences available via RedFly database (http://redfly.ccr.buffalo.edu), several enhancers were found in this region that target lncRNA *sphinx* and the transcription factor *toy* residing within this novel region although for some enhancers their target genes are not known. One can speculate that these enhancers could target genes co-located in 3D, i.e. residing within the same subnetwork centered around bin 1220.

In the extended DGRP network two novel bins, 2 and 28, were found to have the highest degree. Seven and 17 genes residing in subnetworks centered around bin 28 (chr2L: 2160000-2240000) and bin 2 (chr2L:80000-160000) were enriched in the ‘immune system process’ and ‘cellular response to stress’ GO terms, respectively. Some of these genes have previously been implicated in aging or have phenotypes which could be linked to longevity. For example, flies heterozygous for the mutation in the *Stat92E* gene have been found to have maximum lifespan up to 30% shorter than those of wild-type control flies [33]. The mean lifespan of *Drosophila* was found to be increased through post developmental RNA interference of *GlyP* by up to 17.1% [34]. Another gene listed in Table 1 found to have a positive effect on lifespan is *Cat*, where an overexpression of this gene results in an increase in lifespan by up to a third [35]. Searches in the FlyBase database show that several other genes have phenotypes associated with aging, e.g. *Clbn* and *Atg16* genes have a ‘short-lived’ phenotype, the *BI-1* gene has both a ‘short-lived’ and ‘long-lived’ phenotype and genes *kay* and *HipHop* have phenotypes for increased mortality.

Using the RedFly database, we found that the novel region on chr2L:2160000-2240000 (bin 28), which was added to the original nodes of the DGRL GWAS-based network on the basis of its strong interactions with the original nodes, harbors several enhancers. Some of these enhancers target *CG34172*, *Uch* and the transcriptional-repressor protein *aop* genes. The latter strongly associates with longevity and is found to be central to lifespan extension caused by reduced IIS or Ras attenuation [36]. For some enhancers their target genes were not specified. One can speculate that these enhancers could target other co-located genes residing within the subnetwork centered around bin 28.

### Clusters in the extended GWAS-based networks

**Figure 1:**
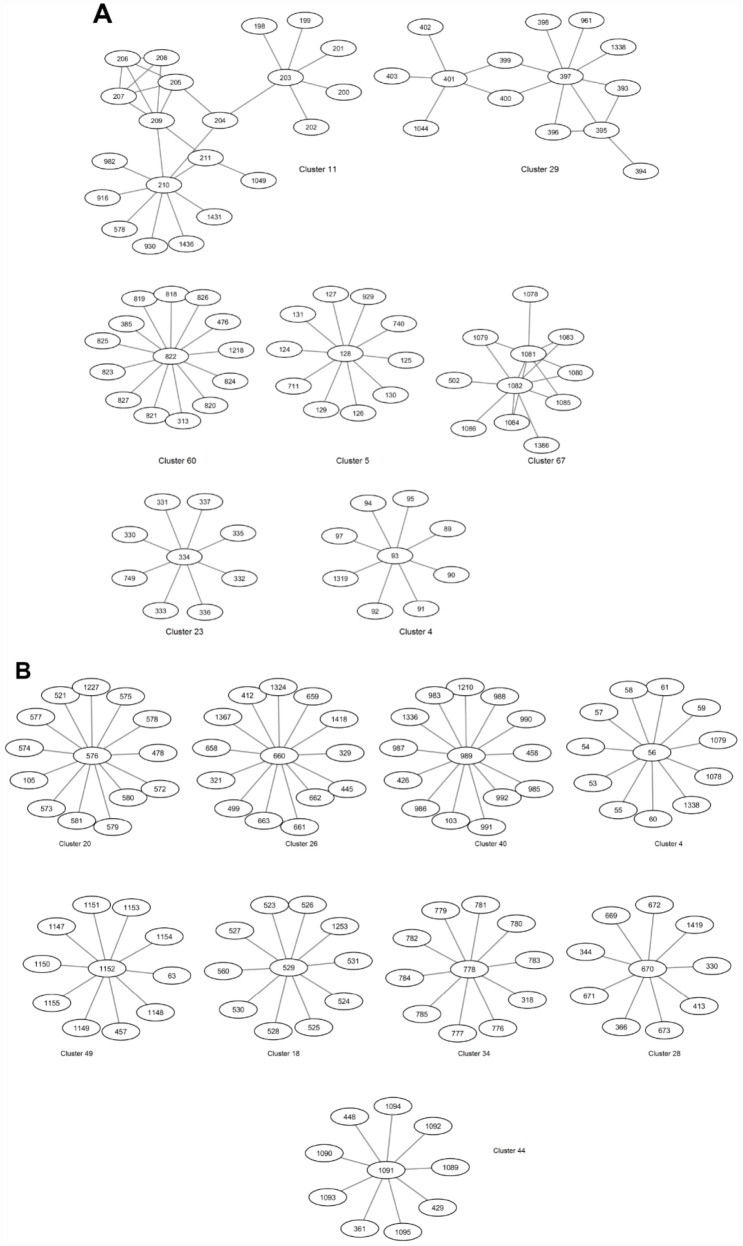
Selected clusters in the Synthetic (**A**) and DGRP (**B**) GWAS-based extended networks of interactions.

Community detection algorithm implemented in GEPHI which uses the Louvain modularity method [37] was performed to identify clusters in the Synthetic and DGRP GWAS-based networks. Selected clusters are shown in Figure 1. Complete sets of clusters for each network are shown in Supplementary Tables 3–4. A ‘resolution’ parameter was set to 0.1, enabling us to identify more communities/clusters as compared with the smaller number of communities that could be obtained by using a greater value for this parameter [38]. These clusters were further explored with the aim of identifying novel genes that co-locate with known longevity-associated genes and are enriched in the same biological function as known genes.

### Clusters in the extended Synthetic GWAS-based network

The Synthetic GWAS-based network was found to have 81 communities/clusters with the smallest consisting of three nodes and the largest of 72 nodes (see Supplementary Table 3). Selected clusters with the most significant enrichment in longevity-associated GO terms are summarized in Table 2. Nodes constituting these clusters are listed in Supplementary Table 5.

**Table 2:** Number of nodes in the selected clusters in the Synthetic GWAS-base network and genes enriched in longevity-associated GO terms.

**Cluster**	**GO term**	**P-value**	**Genes enriched in GO term**	**Number of nodes harboring genes enriched in GO term/ total number of nodes**
4	cellular response to stimulus	0.004503	Rab39, Tom40, santa-maria, Mnn1*, sem1, Pvf2, **Gr28b**, Pvf3, Ziz, RapGAP1, Wnt4, wg, Wnt6, Wnt10**, ninaC, CG5160, CG5181, mir-305	6/9
4	localization	0.007119	*Rab39, Tom40, Sem1, Pvf2, CG13793, CG13794, CG13795, CG13796, CG31904, CG31907, CG33296, Pvf3, Ndae1, Wnt4, ninaC, Ntl, ATPsynGL, Nuf2*	5/9
4	cell communication	0.023993	*Rab39, santa-maria, **Mnn1**, Pvf2, Gr28b, Pvf3, Ziz, RapGAP, Wnt4, wg, Wnt6, Wnt10, ninaC, CG5160, mir-305*	7/9
5	macromolecule modification	0.003413	***Atg1**, Ptp69D, Cnot4, RluA-1, CG32847, CG33303, CG34183, CG42366, Fkbp59, CG4839, Ror, CG4968, Sps2, gny, STUB1, Sp27A, LManI, Bug22, Cdk1, Cand1, Usp14, **CYLD**, Utx, **Pten**, **bsk**, Dref, RluA-2, LMannII, FBXO11*	9/11
5	cellular catabolic process	0.020971	***Atg1**, lft, CG32847, CG4592, CG4594, CG4598, yip2, Prosalpha6, RpS27A, CG5367, Usp14, Utx, **Pten**, CG5676, **bsk**, **chico**, CG5731, CG8526, FBXO11*	9/11
11	DNA repair	0.021953	*CG17329, **ku80**, CG31807, CG33552, EndoGI, CG5316*	5/21
11	developmental process	0.010492	***cact**, Cas, chif, cni, crp, dac, **foxo**, fzy, glu, goe, grp, heix, her, mdy, mir-9b, mir-9c, sing, squ, twe, wek, yellow-b, BicC, BuGZ, CG17328, CG32572, CG4793, CG5953, **Ca-alpha1D**, Cyp303a1, Cyt-c-d, EndoGI, GMF, Idgf1, Idgf2, Idgf3, Mhc, Npc2b, Syx5, TwdIX, TwdIY, TwdIZ, TwdIaplha, **VhaSFD**, beat-Ia, beat-Ib, beat-Ic*	14/21
23	apoptotic process	0.033954	***azot**, tor, **cathD**, Cul1, fwe, mir-263b*	4/9
23	positive regulation of gene expression	0.028920	*CG12769, Rpt1, Kdm4A, udd, **Nup50**, nito, CG6244, Lpin, lig*	6/9
29	negative regulation of transcription, DNA-templated	0.024472	*CG10038, spt4, wuc, Iz, seq, Kdm4B, **sug**, **Psc**, **Su(z)2**, Iswi*	7/14
60	gene expression	5.6 × 10^-4^	*CG10474, Rpb8, sa, CG11906, mip40, Pc, croc, barc, CRIF, Hr78, wbl, rib, Tsr1, eg, CycH, CG7414, Nopp140, **mub**, RpLPO, Cdk12, TfAP-2, **rho-7***	9/14

* Genes previously found to have association with longevity as recorded in FlyBase or GeneAge resources are shown in bold.

**Genes residing within original nodes, i.e. harboring SNPs with D > 7.9 are underlined.

Six genes residing within five nodes of cluster 11 were enriched in the ‘DNA repair’ GO term (p-value = 0.022). DNA integrity and stability depend upon the ability of DNA repair mechanisms to detect and repair damaged DNA. A DNA repair gene *ku80* is involved in repair of double-stranded DNA breaks [39] and was found to have a ‘short-lived’ phenotype. The *EndoGI* gene is involved in positive regulation of the Notch signaling pathway and associated with an ‘increased mortality’ phenotype. Notch signaling is important for cell-cell communication and plays an important role in processes such as neuronal function and development (reviewed in [40]). The other four genes residing within this cluster, *CG17329*, *CG31807*, *CG33552* and *CG5316*, are currently not fully characterized. One can speculate that close proximity of these genes within the cell nucleus and shared biological function with the *ku80* and *EndoGI* genes, make them potential candidate genes, linked to longevity. In fact, the human ortholog of the *CG5316* gene identified via the Integrative Ortholog Prediction Tool available at https://www.flyrnai.org/cgi-bin/DRSC_orthologs.pl, the *APTX* gene, which encodes the DNA strand-break repair protein aprataxin was found to have a broader role in DNA single-strand break repair in neurodegenerative disease (reviewed in [41]) that shortens lifespan. In a longitudinal study with 11 years of follow-up on survival in the oldest-old Danes, Soerensen et al. [42] have shown that rs705649 SNP in DNA repair protein XRCC5, which is the human ortholog of the *ku80* gene, is associated with mortality in late life.

Six genes, *azot, tor, cathD, Cul1, fwe* and *mir-263b*, residing in cluster 23 were enriched in the ‘apoptotic process’ GO term (p-value = 0.034). The apoptotic process has almost an opposite role to the previously discussed GO term ‘DNA repair’, whereby when DNA is damaged, the checkpoint protein p53 is activated and the decision is made as to whether replication should be stopped and the DNA repaired, or the cell made to die by apoptosis (reviewed in [43]). Studies have found that in mammals, at least in part, apoptosis plays an important role in the process of aging and tumorigenesis and that age-enhanced apoptosis may work as a protective mechanism against age-associated tumorigenesis [44]. The ahuizotl gene, *azot*, which encodes a calcium dependent protein responsible for the elimination of less fit cells, is known to play a role in delaying aging and extending lifespan. This gene has the ‘long-lived’ phenotype and was previously found to increase lifespan [45]. Another gene in this group, *cathD*, a gene with phenotypes that include those that associate with apoptosis such as ‘increased cell death’ as well as longevity associated phenotype ‘short-lived’. The human ortholog of this gene, *CTSD*, encoding cathepsin D was found to associate with cognitive abilities in both demented and non-demented individuals [46] and was also implicated in increasing the risk of developing Alzheimer's disease [47]. Another gene in this group, *Cul1*, belongs to the cullin family and has phenotypes of ‘increased mortality’ and ‘neuroanatomy defective’. The *fwe* gene encodes a transmembrane protein that mediates win/lose decisions in cell competition and neuronal culling during development and aging; this gene has longevity related phenotypes - ‘increased mortality’ and ‘lethal’. Given the longevity association that these genes in this cluster have, through both phenotypes and biological functions, one can speculate that other genes that are found to reside within this cluster 23 may also influence longevity in the same way as the genes discussed above, due to their close proximity and strong interaction in the genome.

Four out of 19 genes enriched in the ‘cellular catabolic process’ GO term (cluster 5; Table 2) have been previously shown to have association with longevity or display phenotypes which associate with aging and, in most cases, with increased lifespan. This includes *chico*, a gene encoding an insulin receptor substrate that belongs to an insulin/insulin-like growth factor (IGF) signaling pathway and found to increase lifespan by up to 48% [48]. Koohy et al. [49] have identified transcriptional downregulation of components of the insulin-like growth factor signaling pathway in mouse, in particular downregulation of the mouse homolog of *chico* gene, *IRS1*, as a signature of aging in developing B cells. The overexpression of the *Pten* gene was found to delay the process of proteostatis and therefore resulted in a decrease in the loss of muscle strength during muscle aging, increasing maximum lifespan in *Drosophila* by up to 7.7% in comparison with matched controls [50]. Interestingly, the human *PTEN* (phosphatase and tensin homolog) gene was found to encode upstream regulators for the *FOXO3* gene [51], one of the few loci robustly associated with longevity in humans [10], stressing that longevity-associated SNPs may reside in regulatory regions as well as in protein-coding genes [52]. The *Drosophila* ortholog of the *FOXO3* gene, *foxo*, a transcription factor involved in the regulation of the insulin signaling pathway, is a commonly known longevity gene [53–56]. The 80 Kb region harboring this gene interacts with the *Pten* region although the interaction frequency is below the threshold chosen in this study. The neuronal-specific upregulation of the *Atg1* gene was found to result in increased median lifespan of *Drosophila* by up to 25% [57]. The human ortholog of this gene, *ULK1*, involved in longevity-regulating pathways identified by the KEGG database [58]. Salas-Pérez et al. [59] have shown that methylation level of the CpG region residing within this gene strongly associates with age-related obesity and metabolic syndrome traits, suggesting a role for DNA methylation in aging-related metabolic alterations. Another gene found to be enriched in the ‘cellular catabolic process’ GO term was the *bsk* gene which is involved in RNA interference. Such interference in intestinal stem cells results in short life due to impaired intestinal homeostasis and tissue regeneration and has been found to reduce mean lifespan by 16.4% and 10.2% in males and females, respectively [60].

Several genes residing within other clusters and enriched in longevity-related GO terms have been previously implicated in longevity. The overexpression of the *VhaSFD* gene that encodes a regulatory subunit of the vacuolar ATPase proton pump (H+-ATPase) and Sugar baby (*Sug*) gene related to a maltose permease from *Bacillus* result in an increase in mean life span by 5–10% [61]. The *mushroom–body expressed* (*mub*) gene has previously been found to have an association with longevity when mutated; the insertion of a p-element in the gene resulted in an increased lifespan up to 21.4% [62]. On the contrary, the *rho-7* gene was found to decrease lifespan; the knocked-out study showed that flies develop severe neurological defects as well as a greatly reduced lifespan [63]. Several genes in Table 1 were found to display the phenotype ‘increased mortality’ according to FlyBase resources. These includes the *CYLD* gene, a cancer consensus gene responsible for tightly limiting the immune response duration [64]. A mutant of this gene, *dCYLD*, was proven to be essential for JNK (Jun-N-terminal Kinase)-dependent oxidative stress resistance and normal lifespan and has also been indicated to play a critical role in modulating TNF-JNK-mediated cell death [65]. The *Mnn1* gene that also play a role in the regulation of stress response in *Drosophila* [66] displays this phenotype. The association between stress and lifespan has often been made, and previous studies have observed differences in gene expression when comparing normal and stress conditions which has resulted in the identification of aging genes in *Drosophila*. The genes found to reside in the same clusters as the genes previously shown to play roles in biological processes associated with longevity were found to harbor a number of SNPs. Although not all SNPs residing within genes enriched in the same GO term had a significant *D*-value (*D* > 7.9), one can speculate that SNPs in one or several functionally-related gene(s) co-located within the cell nucleus may contribute collectively to the longevity phenotype.

### Clusters in the DGRP GWAS-based network

The DGRP GWAS-based network comprised 61 communities, where the smallest consisted of three nodes and the largest of 42 nodes (see Supplementary Table 4). Selected clusters with the most significant enrichment in longevity-associated GO terms are summarized in Table 3. Nodes constituting these clusters are listed in Supplementary Table 6.

**Table 3:** Number of nodes in the selected clusters in the DGRP GWAS-base network and genes enriched in longevity-associated GO terms.

**Cluster**	**GO term**	**P-value**	**Genes enriched in GO term**	**Number of nodes harboring genes enriched in GO term/ total number of nodes**
4	development growth	0.030823	Elp3, ine, bdl, ft*, CASK, tsl	4/12
18	nervous system process	0.033071	Or59c, bw**, Gr59c, Gr59a, Gr59b, Gr59d, Gr59e, Gr59f, Or59b, Or59a, tko	4/11
20	organelle assembly	0.016230	*Oseg2, Pp2A-29B, Rcd4, sls, Oseg4, CG42787, hts, Cnb, RpL11, Ar16, mtsh, RpL23A*	9/14
20	immune system process	0.035555	*CG10764, asrij, HBS1, sls, Rap1, ac, ecd, cnk, Ostgamma, Bgb, Bro, **Btk29A**, par-1*	9/14
26	regulation of immune system process	0.019532	***Traf6**, **PGRP-SA**, CG1572, Cyt-b5, GNBP3, GstO2, **Sod2**, Spn42Dd*	8/14
34	response to stimulus	0.031506	*geko, skl, AstC-R2, Adf1, Dic4, Trap1, geminin, Bap170, **Debcl**, **Chmp1**, GNBP2, not, CG4306, **rpr**, **grim**, hid, CG6893, GNBP1*	8/11
40	open tracheal system development	0.001555	*stumps, Cad88C, cv-c, grh, btsz, thr, put, scb*	5/14

* Genes previously found to have association with longevity as recorded in FlyBase or GeneAge resources are shown in bold.

**Genes residing within original nodes, i.e. harboring SNPs with p<3.33×10^-5^ are underlined.

Thirteen genes in cluster 20 and eight genes in cluster 26 were enriched in the ‘immune system process’ (p-value = 0.036) and ‘regulation of immune system process’ (p-value = 0.0195), respectively. Immune senescence is the deterioration of immune function with age. As well as resistance to infection, immunosenescence may also reduce resistance to cancer and chronic activation of the immune system, usually as a result of autoimmune diseases, cancer, HIV infection and other chronic infections. The changes in immune response were found to be very similar to the changes that occur in elderly individuals [67]. In response to aging most physiological functions are altered, e.g. the declination in cellular and humoral immunity. The most sensitive immune cells to aging appeared to be T cells, and the most critical component of immunological aging is known to be changes in the T lymphocyte compartment, concluded by studies on aging in humans [68], documenting significant changes in the functional and phenotypic profiles of T cells. Further analysis of literature has also suggested that the inability of the innate immune system to work efficiently is a contributing factor to the development of many diseases observed in the elderly [69].

Several genes shown in Table 3 have been found previously to have association with longevity, with many of them being associated with a decrease in life span. It has been found that *Drosophila*, heterozygous for the tumor suppressor gene *ft,* had a shorter lifespan, where it was suggested that this mortality effect was associated with the interaction between this *ft* tumour suppressor and signal transduction pathways mediated by the Hippo pathway [70]. Phenotype searches for genes in this table found *grim*, *Btk29A* and *tko* to express the phenotype ‘increased mortality’ whereas *Chmp1* was found to express the phenotype ‘short-lived’. An increase in the proapoptotic protein *grim* has been shown to significantly reduce lifespan in female drosophila by up to 34% in median lifespan and 25% in maximum lifespan [71]. The *Btk29A* and *Traf6* genes are FOXO targets in the JNK signaling pathway. This signaling pathway is stress-activated and involved in developmental and metabolic regulation, immune responses and lifespan extension [72–73].

The *Sod2* gene has been observed, in separate studies, to have both a positive and negative effect on lifespan in *Drosophila*. When overexpressed, the gene was found to result in a 20% increase in both mean and maximum lifespan [74] whereas RNA interference-mediated silencing of the *Sod2* gene caused an increase in oxidative stress leading to early-onset mortality in young adults [75]. The *PGRP-SA* gene has also been observed as one of few genes to show age-related changes in expression without being affected by diet, allowing this gene to be considered a candidate marker of aging [76].

### SNPs in non-coding regions

Total of 26,499 and 653,030 non-coding SNPs were recorded in the Synthetic and DGRP GWAS datasets, respectively. First, we explored whether these SNPs tend to occur within border regions separating adjacent topologically associated domains (TADs). Second, using intra-chromosomal Hi-C data with finer resolution we explore potential target genes for SNPs residing in non-coding regions utilizing co-location of SNP- and gene-harboring loci.

### SNPs in Topologically Associated Domain (TAD) boundary regions

Approximately 2% (11,982) of all SNPs recorded in non-coding regions in the DGRP GWAS dataset were found in TAD boundary regions as compared to 9,321 SNPs in controls. Fisher’s exact test shows that TAD boundary regions are enriched in SNPs (p=1.0376×10^-75^). These SNPs were found in 998 (~35%) of all TAD boundary regions. On the contrary, just a small proportion of SNPs from the Synthetic GWAS dataset were found within TAD boundary regions.

In the absence of individual genotype data, it is extremely difficult to assess the effect that SNPs in TAD borders may have on the genes residing within a given TAD. We assumed that one of the observed manifestations of latent changes in patterns of interactions between genomic regions could be in longevity-associated genes known to share both ‘long-lived’ and ‘short-lived’ phenotypes. We hypothesized that a SNP(s) in nearby TAD borders may lead to a disruption of a regulatory pattern of these genes resulting in one of the phenotypes, either ‘long-lived’ or ‘short-lived’, whereas the opposite phenotype could be caused by SNPs residing within genes themselves. Genomic positions were available for 124 out of 131 genes recorded in FlyBase resources as sharing both ‘long-lived’ and ‘short-lived’ phenotypes. We found that the majority of these genes, 106, were residing within 30 Kb regions spanning bins harboring a TAD border and including ±10 Kb of flanking regions (i.e. two adjacent bins). From these genes, 43 were found to reside in the vicinity of 51 TAD borders that harbor SNPs; 89 genes were found to reside in the vicinity of 120 TAD borders that don’t harbor SNPs. Thirty of these genes were found in the vicinity of both mutated and non-mutated TAD borders. (Note that the length of the topologically associated domains in our dataset for *Drosophila* varies between ~2 and 436 Kb that leads to the same gene being in the vicinity of two or more borders depending upon its length). Seventeen genes including *Charon*, *foxo*, *Jafrac1*, *mei-9* and *sun* occurred exclusively in the vicinity of mutated TAD borders (see Supplementary Table 7). Based on these observations one can speculate that SNPs residing in border regions of TADs may disrupt regulatory pattern of longevity related genes in the corresponding TADs by forming looping interactions with regulatory elements residing in the adjacent TADs potentially leading to the change of function, e.g. phenotype.

### Target genes for SNPs in non-coding regions

It is often assumed that a SNP residing in non-coding regions could potentially occur within a regulatory region(s) for a nearby gene(s). In many cases, if this nearby gene carries out biological functions which are related to the disease being studied, the SNPs found are automatically become a subject of further investigations. However, various looping interactions could happen between seemingly remote DNA fragments. For example, Sahlén et al. [77] have observed looping interactions between different promoters and postulated that promoters can also have enhancer activity influencing the expression of other genes not necessarily the nearest ones [78].

Analysis of the intra-chromosomal Hi-C data at 10 Kb resolution shows that in most cases interactions between adjacent bins are the strongest. This observation justifies the extension of gene boundaries to include SNPs residing within ±10 Kb of flanking non-coding regions as it is often done in analyses of GWAS data. However, there are regions harboring non-coding SNPs recorded in the Synthetic GWAS dataset and DGRP GWAS datasets for which the strongest interacting regions were as distant as 50 Kb and 100 Kb, respectively. Thirty of these top long-range interacting pairs in both datasets were selected for further investigation. The summary of these regions is given in Supplementary Tables 8–9.

The calculated *D*-value for only one non-coding SNPs from the Synthetic GWAS dataset in the selected regions residing in bin 2006 (chr2L:20170000-20180000; Supplementary Table 8) exceeded the significance level (*D*=12.009 >7.9). The strongest interacting region for this bin was found 30 Kb upstream of the SNP. Not a single SNP chosen from the DGRP GWAS dataset residing within the selected regions meets the genome-wide significance level which in this case was set to 4.22×10^-6^.

A total of 73 and 59 genes were found in the top 30 regions selected for SNPs from the Synthetic and DGRP GWAS datasets, respectively. Several bins, 1165, 4366, 7816 and 7887, which correspond to regions chr2L:11,680,000-11,690,000, chr3L:380,000-390,000, chr3R: 10,760,000-10,770,000 and chr3R:11,470,000-11,480,000, respectively, were gene-less. Further analysis of potential target genes residing within long-range interacting regions using FlyBase resources have found many genes that share phenotypes that could be associated with longevity, e.g. ‘increased mortality’, ‘lethal’ and ‘immune response defective’ (Tables 4 and 5). Only one gene, *AttC*, encoding an immune inducible peptide homologous to antibacterial peptides having activity against Gram-negative bacteria was previously considered to be a candidate marker of aging [76]. For the interacting bins containing more than one gene with longevity related phenotypes, we can speculate that non-coding SNPs could reside in an enhancer and this single enhancer may target all these genes, influencing their expressions and phenotypes. Phenotypes of potential target genes and their human orthologs are summarized in Tables 4–5.

**Table 4:** Phenotypes of genes, found in regions most strongly interacting with regions containing non-coding SNPs from the Synthetic GWAS dataset, and their human orthologs.

**SNP- harbouring bin**	**Possible target gene**	**Longevity related phenotypes**	**Human ortholog**
4566	*CG45186*	lethal; increased mortality during development; increased mortality	*SVIL*
	*CG32298*	partially lethal - majority die; flightless	
5660	*SNCF*	lethal - all die during P-stage	
	*CG14107*	partially lethal - majority die; some die during pupal stage; lethal - all die during P-stage	
1608	Ca-alpha1D*	increased mortality during development; lethal - all die before end of P-stage	*CACNA1D, CACNA1S*
2464	*jing 16*	locomotor behavior defective; cell death defective	*AEBP2*
3149	*AttC 3*	partially lethal; some die during pupal stage; neuroanatomy defective	
4840	*CG4597*	some die during pupal stage; partially lethal - majority die	
	*CG4611*	lethal - all die during P-stage	*PTCD1*
5705	*Hml*	immune response defective	*SSPO, VWF, OTOG, MUC5B*
7886	*CG43335*	partially lethal - majority die; some die during pupal stage; partially lethal	
554	*GluRIIA*	locomotor behavior defective; neurophysiology defective; neuroanatomy defective; lethal	
554	*GluRIIB*	neuroanatomy defective; neurophysiology defective	
943	*numb*	decreased cell number; some die during embryonic stage; increased mortality; increased cell number; lethal - all die before end of prepupal stage; flight defective; tumorigenic	*NUMBL*
996	*bib*	lethal - all die before end of pupal stage	
1176	***crol***	locomotor behavior defective; increased occurrence of cell division; increased mortality; cell death defective	*ZNF569, ZNF99, ZNF841, ZNF814*

* Genes previously found to have association with longevity as recorded in FlyBase or GeneAge resources are shown in bold.

**Table 5:** Phenotypes of genes, found in regions most strongly interacting with regions containing non-coding SNPs from the DGRP GWAS dataset, and their human orthologs.

**SNP- harbouring bin**	**Possible target gene**	**Longevity related phenotypes**	**Human ortholog**
2962	*en*	lethal - all die during embryonic stage; size defective; planar polarity defective; increased cell death; some die during pupal stage; partially lethal - majority die	
2453	*Pld*	developmental rate defective; partially lethal - majority die; some die during embryonic stage; neurophysiology defective; lethal - all die before end of embryonic stage	*PLD2*
2463-2464	*jing*	locomotor behavior defective; cell death defective	*AEBP2*
4366	*trh*	neuroanatomy defective; partially lethal - majority die; lethal; some die during embryonic stage; lethal - all die before end of embryonic stage	*NPAS1*
4566	*CG45186*	lethal; increased mortality during development; increased mortality	*SVIL*
	*CG32298*	some die during pupal stage; partially lethal - majority die; flightless	
5660	*SNCF*	lethal - all die during P-stage	
	*CG14107*	partially lethal - majority die; some die during pupal stage; lethal - all die during P-stage	
1608	Ca-alpha1D*	increased mortality during development; lethal - all die before end of P-stage	*CACNA1D, CACNA1S*
2286	*RpL38*	increased mortality; increased mortality during development; developmental rate defective	*RPL38*
2375	*laccase2*	lethal; partially lethal; lethal - all die during embryonic stage;	*HEPHL1, CP, HEPH*
3149	*AttC*	partially lethal; some die during pupal stage; neuroanatomy defective	
4840	*CG4597*	some die during pupal stage; partially lethal - majority die	
	*CG4611*	lethal - all die during P-stage	*PTCD1*
5705	*Hml*	immune response defective	*SSPO*, *VWF*, *OTOG*, *MUC5B*
7636	*timeout 1*	increased mortality; lethal - all die before end of P-stage; some die during P-stage	*TIMELESS*
7886	*CG43335*	partially lethal - majority die; some die during pupal stage; partially lethal	
8955	*CG33970*	lethal; sleep defective; flightless	

* Genes previously found to have association with longevity as recorded in FlyBase or GeneAge resources are shown in bold.

Several genes with longevity-associated phenotypes were common between two datasets: *CG45186*, *CG4611*, *jing*, *Ca-alpha1D*, *Hml*, *CG32298*, *SNCF*, *CG14107*, *AttC*, *CG4597* and *CG43335*. The first three genes in this list have matched human orthologs *SVIL*, *PTCD1* and *AEBP2*, respectively. The *Ca-alpha1D* gene was found to match two human genes, *CACNA1D* and *CACNA1S*, whereas the *Hml* gene has four human orthologs: *SSPO*, *VWF*, *OTOG* and *MUC5B*. The human *SSPO* gene is involved in the modulation of neuronal aggregation and was suggested to be involved in developmental events during the formation of the central nervous system (https://www.uniprot.org/uniprot/A2VEC9). Dysregulation of the *CACNA1D* gene and loss-of-function mutations in the *SSPO* gene were found to associate with age-related diseases such as Alzheimer’s [79] and Parkinson’s [80]. Although no other human orthologs have been previously implicated in longevity, one can speculate that SNPs in non-coding regions may target these genes remotely in a similar way as was found in *Drosophila* and play a role in longevity.

## CONCLUSIONS

In this study we applied a conceptually new approach for identification of novel genes associated with longevity in *Drosophila* and provided the evidence for using co-location of genes/genomic regions governed by the 3D architecture of the *Drosophila* genome for predicting these novel genes. First, we created networks of interactions between genes and genomic regions harboring SNPs that meet a predefined level of significance for each GWAS dataset by using intra- and inter-chromosomal interaction frequencies (Hi-C data) as a measure of co-location. Then each of these networks was extended by adding regions that co-locate with the existing regions. We identified several genes residing within these newlyadded regions both known to associate with longevity and the novel ones that were not originally included in the analysis. Community detection algorithm identified several tightly-knit clusters in both networks. Genes residing within the same clusters were found to be enriched in longevity-related GO terms including ‘DNA repair’, ‘apoptotic process’, ‘nervous system process’ and ‘immune system process’. Using literature search and additional bioinformatics resources we investigated the plausibility of genes found to have genuine association with longevity. Our network approach identified several novel genes (see Tables 1–3) with no prior known associations with longevity as well as genes with prior reported associations with longevity, acting as a proof of concept. Among these genes are the *Vps16B, Cad99C, DPCoAC* (*CG4241*) and *Mtl* genes residing within important nodes in the DGRP GWAS-based network and, together with the known longevity-associated genes *aop, Stat92E, GlyP,* being enriched in ‘immune system process’ GO term. The *Cad99C* gene, which encodes a member of the cadherin superfamily of transmembrane proteins, harbors an SNP (chr3R:25674492) which is present in both GWAS datasets. Another novel gene, *CG5316*, co-located with genes *CG17329*, *CG31807*, *CG33552,* *EndoGI* and the longevity-associated gene *ku80* in the Synthetic GWAS-based network, was found to be enriched in ‘DNA repair’ GO term. Although the function of this gene is unknown, its human ortholog ‒ the *APTX* gene ‒ was implicated in longevity [41]. A group of genes ‒ *CG1572*, *Cyt-b5*, *GNBP3*, *GstO2* and *Spn42Dd* ‒ residing within the cluster 26 in the DGRP GWAS-based network together with the known longevity associated genes *Traf6*, *PGRP-SA* and *Sod2* are also strong candidates for novel longevity genes. None of these genes harbor SNPs that reach genome-wide significance level in the DGRP GWAS dataset. One gene, the glutathione S transferase O2 gene *GstO2*, harbors one SNP with genome-wide significant D-value 8.38 in the Synthetic GWAS dataset. Interestingly, all these genes reside in close proximity (i.e. within 30 Kb region) to TAD borders. Three of the nearest TAD border regions harbor SNPs prompting speculations that these SNPs could influence longevity either separately or together with SNPs residing within the genes. This observation could explain the enrichment of TAD borders in SNPs.

Many of these newly found genes harbor SNPs that do not reach a predefined genome-wide significance level, leading to speculation that SNPs residing within genes enriched in the same GO term may influence longevity collectively (when one or several SNPs in these functionally-related genes occur in the same fly to cause a phenotype) rather than individually when a single SNP in one of these genes could cause a phenotype. Further, we explored potential target genes for SNPs in non-coding regions also assuming co-location of SNP-harboring loci and target genes within cell nucleus. Several novel target genes for non-coding SNPs have been identified using our network approach (see Tables 4–5) including genes such as *CG45186*, *CG4611*, *Ca-alpha1D*, *Hml*, and *AttC* that are common between two datasets and have human orthologs associated with age-related diseases.

Further experimental validation is required in order to establish the functional significance of the genes and SNP-target gene pairs found. This computational approach promises to be a stepping stone to identification of novel genes and SNP targets linked to longevity in humans and further understanding of genetic factors associated with this phenotype.

## MATERIALS AND METHODS

### *Drosophila* GWAS data used in this study

Two datasets of SNPs summary statistics generated by GWA studies reported in Burke et al. [19] and Ivanov et al. [20], containing respectively ~1 million and ~2 million SNPs, were used in this study. The first dataset was obtained from a “synthetic” population of *Drosophila* derived from a small number of inbred founders. Two independent sets of seven inbred *Drosophila* lines with another founding line added to both sets, were crossed to initiate two synthetic recombinant populations, A and B. Populations A and B were then maintained as four independent large populations (A1/A2, B1/B2). Next-generation sequencing was used to identify allele frequencies in the ‘young’ control group, comprising 120 14-day-old females, and the last surviving ~2% of females from the remaining cohort (an ‘old’ group). The occurrence of SNP in each of the eight ‘old’ populations and eight ‘young’ control populations was recorded, resulting in ~1.2M SNPs in the A populations and ~1.1M SNPs in the B populations (see [19] for details). The SNPs for both populations were combined; duplicated SNPs were recorded only once with haplotype allele frequencies combined. Henceforth, we will refer to this data set as the Synthetic GWAS data.

The second dataset was obtained by GWAS performed on The Drosophila Genetic Reference Panel (DRGP), Freeze 2.0 [20], which comprises 205 *D. melanogaster* lines derived from 20 generations of full-sib mating from inseminated wild-type caught females from Raleigh, North Carolina. Lifespan data was available for virgin females for 197 DGRP lines, with ~25 females per line. A total of 2,193,745 SNPs was recorded together with the corresponding p-values, quantifying association with lifespan. P-values were calculated using linear regression under an additive model with four first principal components and the presence of *Wolbachia pipientis* included as a covariate (see [20] for details). Henceforth, we will refer to this data set as the DRGP GWAS data.

SNPs were considered to be in coding regions of certain genes if they resided between gene start and gene end positions as defined by BDGP Release 6/dm6 assembly [81] and recorded in FlyBase database (http://flybase.org/). All other SNPs were considered to reside within non-coding regions.

### Intra- and inter-chromosomal interaction (Hi-C) data

A dataset of intra- and inter-chromosomal normalized contacts (interaction frequencies) between 1503 80 Kb regions (bins) obtained by Sexton et al. [21] was downloaded from GEO database (accession number GSM849422). In this dataset bins 1-287 correspond to Chromosome 2L, bins 288-551 to Chromosome 2R, bins 552-858 to Chromosome 3L, bins 859-1207 to Chromosome 3R, bins 1208-1223 to Chromosome 4 and bins 1224-1503 to Chromosome X. In addition, a dataset of intra-chromosomal interaction between 11,839 10Kb regions was downloaded from the same GEO database.

### Dataset of Topologically Associated Domain (TAD) boundary regions

For each of 2,847 TAD borders compiled by [82] 100 bp of flanking sequences were added from both sides to create a dataset of TAD border regions. Using SNP position data, non-coding SNPs residing in each TAD border region in the GWAS datasets were counted. Matched control dataset was generated as follows. For each TAD border a random border was generated by randomly selecting a position on the matching chromosome and adding ±100 bp of flanking sequences not overlapping with any “real” border region. This process was repeated 100 times. The number of SNPs residing within generated control datasets were counted and averaged across 100 control datasets. Fisher’s Exact Test was then used to assess the overrepresentations of SNPs within TAD borders.

### Pre-processing of Synthetic GWAS data

To identify positions with divergent haplotype frequencies in the young (control) and old groups in this dataset, Euclidean distances between the control and old groups were calculated for haplotype data for populations A1/A2 and B1/B2 combined. All duplicates were removed. The distance, *D*, for a given SNP was calculated as suggested in Burke at al. [19]:

$$D=100.\sqrt{\frac{\overset{n}{\underset{j=1}{\sum }}{\left(h_{O,j}-h_{Y,j}\right)}^{2}}{n}}$$

where *h_o,j_* is the haplotype frequency of the *j^th^* founder in the old samples, *h_Y,j_* is the haplotype frequency of the *j^th^* founder in the young control sample, *n* is the number of haplotypes found at that position. For SNP positions with combined haplotype frequencies, the *n* in the equation used was altered accordingly. SNP positions with the largest calculated *D* values were those showing the largest differences between haplotype frequencies in the control and old groups, and it was therefore these SNPs that were indicated as most likely to have association with longevity. Following Burke et al. [19], *D* > 7.9 was considered to correspond to genome-wide significance p-value < 0.05.

### Identification of candidate longevity genes and potential regulatory regions

To align SNP positions with Hi-C data, SNPs were binned into 80 Kb regions. Start and end positions of each bin and corresponding chromosomes are given in Supplementary Table 10. Each region, harboring SNP(s) meeting the *D* > 7.9 threshold in the Synthetic GWAS data or a predefined p-value threshold (see in the Results and Discussion section) in the DRGP GWAS data, was identified and considered as a node of an *original* network of interactions. Links between the nodes were added to create a network of interactions when an intra- or inter-chromosomal interaction between two nodes was recorded in Hi-C data and the frequency of interaction exceeded a certain threshold. Further, the resulting network was expanded to create *an extended network* by adding extra nodes, corresponding to 80 Kb fragments that interact with the nodes, already present in the original network, with frequencies exceeding a predefined threshold. For each node in networks its degree, i.e. the number of connections a given node has with other nodes, was calculated with the aim of finding influential nodes/regions and novel genes not necessarily covered by GWAS SNP array. In addition, the Louvain modularity method [33] was used to detect communities within the resulting networks, i.e. groups of nodes/regions that are densely connected to each other within a given community but sparsely connected to nodes in other communities of the network. All measures were calculated using GEPHI software tool available at https://gephi.org/.

Genes residing within important nodes/regions of interest were identified using genomic coordinates corresponding to the BDGP Release 6/dm6 assembly [81] downloaded from the FlyBase database (http://flybase.org/). To align the Hi-C data and GWAS SNP positions, all gene positions were lifted over to BDGP Release 5/dm3. This was done using a LiftOver tool (https://genome.ucsc.edu/cgi-bin/hgLiftOver).

### Gene ontology enrichment analysis

The FlyMine software (http://www.flymine.org/) was used to analyze the enrichment of the set of genes, residing within important nodes/clusters, in Gene Ontology (GO) terms for cellular component, biological process and molecular function. Each gene was also sought in the GeneAge database (http://genomics.senescence.info/genes/models.html) of longevity genes and FlyBase resources (http://flybase.org/) as having longevity-related phenotype.

## Supplementary Material

Supplementary Tables

Supplementary Table 1

Supplementary Table 3

Supplementary Table 4

Supplementary Table 7

Supplementary Table 10
