# Supplementary material for: Identification of novel genes associated with longevity in Drosophila melanogaster - a computational approach
Source: Aging (Albany NY). 2019 Dec 3;11(23):11244–67. doi: 10.18632/aging.102527 (PMC6932890; doi:10.18632/aging.102527)
Supplement: Supplementary Tables [file aging-11-102527-s005..pdf]

SUPPLEMENTARY TABLES

Please browse Full Text version to see the data of Supplementary Table 1.

Supplementary Table 1. Number of SNPs residing in regions common between the original GWAS-based networks. Genes with 'long-lived' phenotype are shown in bold.

Supplementary Table 2. Selected subnetworks centered around novel nodes with the highest degree in the extended Synthetic and DGRP GWAS-based networks.

Novel node	Network	All other nodes in subnetwork	Degree	Total number of genes
1220	Synthetic	244, 245, 255, 262, 270, 271, 272, 273, 275, 276, 277, 295, 302, 305, 334, 359, 799, 848, 920, 923	20	188
928	Synthetic	11, 233, 234, 238, 265, 343, 360, 361, 366, 370, 409, 456, 536, 531, 545	15	290
28	DGRP	27, 29, 30, 1063, 1124, 1152, 1179	7	114
2	DGRP	576, 655, 660, 670, 699, 736, 787, 1120, 1131, 1132, 1152, 1178, 1179, 1183	14	255

Please browse Full Text version to see the data of Supplementary Tables 3 and 4.

Supplementary Table 3. Nodes and interactions comprising each cluster of the extended Synthetic GWAS-based networks.

Supplementary Table 4. Nodes and interactions comprising each cluster of the extended DGRP GWAS-based networks.

Supplementary Table 5. List of nodes constituting selected clusters in the Synthetic GWAS-based network.

Cluste r	Nodes in cluster	Total number of	
		nodes in cluster	genes in cluster
4	89, 90, 91, 92, 93, 94, 95, 97, 1319	9	92
5	124, 125, 126, 127, 128, 129, 130, 131, 711, 740, 929	11	229
11	198, 199, 200, 201, 202, 203, 204, 205, 206, 207, 208, 209, 210, 211, 578, 916, 930, 982, 1049, 1431, 1436	21	249
23	330, 331, 332, 333, 334, 335, 336, 337, 749	9	141
29	393, 394, 395, 396, 397, 398, 399, 400, 401, 402, 403, 961, 1044, 1338	14	222
60	313, 385, 476, 818, 819, 820, 821, 822, 823, 824, 825, 826, 827, 1218	14	174
67	502, 1078, 1079, 1080, 1081, 1082, 1083, 1084, 1085, 1086, 1386	11	150

Supplementary Table 6. List of nodes constituting selected clusters in the DGRP GWAS-based network.

Cluster	Nodes in cluster	Total number of	
		nodes in cluster	genes in cluster
4	53, 54, 55, 56, 57, 58, 59, 60, 61, 1078, 1079, 1338	12	125
18	523, 524, 525, 526, 527, 528, 529, 530, 531, 560, 1253	11	155
20	105, 478, 521, 572, 573, 574, 575, 576, 577, 578, 579, 580, 581, 1227	14	256
26	321, 329, 412, 445, 499, 658, 659, 660, 661, 662, 663, 1324, 1367, 1418	14	223
28	330, 344, 366, 413, 669, 670, 671, 672, 673, 1419	10	177
34	318, 776, 777, 778, 779, 780, 781, 782, 783, 784, 785	11	103
40	103, 426, 458, 983, 985, 986, 987, 988, 989, 990, 991, 992, 1210, 1336	14	156
44	361, 429, 448, 1089, 1090, 1091, 1092, 1093, 1094, 1095	10	156
49	63, 457, 1147, 1148, 1149, 1150, 1151, 1152, 1153, 1154, 1155	11	142

Please browse Full Text version to see the data of Supplementary Table 7.

Supplementary Table 7. Genes sharing ‘long-lived’ and ‘short-lived’ phenotypes found in close proximity to mutated and non-mutated TAD border regions.

Supplementary Table 8. Top 30 long-range interacting regions for non-coding SNPs from the Synthetic GWAS dataset.

Non-coding SNP region			Interacting region		
**Bin number**	**Number of SNPs in bin**	**Highest recorded SNP's D-value**	**Strongest interacting bin**	**Distance (Kb)**	**Number of genes in bin**
2367	3	2.727	2362	50	2
3982	7	5.421	3987	50	16
5470	9	5.765	5465	50	13
2459	4	5.170	2463	40	6
4370	12	7.189	4366	40	10
4560	4	3.814	4566	40	10
5656	9	6.284	5660	40	16
11643	2	5.029	11647	40	6
1605	7	7.657	1608	30	9
2006	3	12.009	2003	30	7
2461	1	4.592	2464	30	6
3146	13	3.468	3149	30	13
3447	1	2.160	3444	30	10
4837	8	6.213	4840	30	15
5702	1	3.582	5705	30	8
6981	6	4.577	6978	30	4
7819	4	3.189	7816	30	6
7883	6	5.029	7886	30	11
7884	4	5.989	7887	30	12
8958	11	4.396	8995	30	11
155	14	7.658	153	20	6
229	7	7.550	227	20	10
324	3	5.987	326	20	15
556	11	6.606	554	20	10
592	1	2.066	590	20	12
945	6	4.168	943	20	10
994	5	5.459	996	20	3
1167	10	6.455	1165	20	13
1173	7	6.222	1171	20	10
1174	11	5.694	1176	20	9

Supplementary Table 9. Top 30 long-range interacting regions for non-coding SNPs from the DGRP GWAS dataset.

Non-coding SNP region			Interacting region		
**Bin number**	**Number of SNPs in**	**Lowest recorded SNP P-value**	**Strongest interacting bin**	**Distance (Kb)**	**Number of SNPs**
2388	8	0.3858	2378	100	168
3644	26	0.1303	3637	70	190
2458	34	0.06431	2464	60	171
2367	53	0.05865	2362	50	74
2957	16	0.06397	2962	50	228
3982	256	0.007077	3987	50	205
5470	280	0.003503	5465	50	420
2457	28	0.08089	2453	40	81
2459	73	0.5731	2463	40	120
4370	297	0.008632	4366	40	192
4560	88	0.02264	4566	40	280
5656	222	0.002052	5660	40	307
11643	51	0.05711	11647	40	138
1605	95	0.0236	1608	30	230
2006	64	0.01037	2003	30	153
2124	14	0.0354	2121	30	22
2283	17	0.1617	2286	30	62
2372	68	0.01846	2375	30	128
2456	44	0.01432	2453	30	81
2461	32	0.04341	2464	30	171
3146	377	0.006937	3149	30	295
3447	44	0.13	3444	30	285
4837	181	0.007141	4840	30	359
5702	27	0.01703	5705	30	322
6981	87	0.00301	6978	30	83
7639	11	0.09939	7636	30	126
7819	238	0.001871	7816	30	203
7883	108	0.007967	7886	30	234
7884	120	0.006706	7887	30	255
8958	222	0.001062	8955	30	197

Please browse Full Text version to see the data of Supplementary Table 10.

Supplementary Table 10. Chromosome, start and end positions for each 80 Kb bin.
