# Supplementary material for: Identification of novel genes associated with longevity in Drosophila melanogaster - a computational approach
Source: Aging (Albany NY). 2019 Dec 3;11(23):11244–67. doi: 10.18632/aging.102527 (PMC6932890; doi:10.18632/aging.102527)
Supplement: Supplementary Table 1 [file aging-11-102527-s001..docx]

**Supplementary Table 1. Number of SNPs residing in regions common between the original GWAS-based networks. Genes with ‘long-lived’ phenotype are shown in bold.**

| Gene symbol | Bin | Number of SNPs recorded in | | Number of SNPs with | |
| --- | --- | --- | --- | --- | --- |
|  |  | Synthetic GWAS dataset | DGRP GWAS dataset | D > 7.9 | p < 3.33×10^-5^ |
| *AdoR* | 1183 | 24 | 536 | 0 | 0 |
| *AIF* | 27 | 0 | 33 | 0 | 0 |
| *alrm* | 1124 | 1 | 30 | 0 | 0 |
| ***aop*** | 27 | 17 | 399 | 8 | 0 |
| *Arr2* | 660 | 3 | 60 | 1 | 0 |
| *Atxn7* | 30 | 2 | 83 | 1 | 0 |
| *axo* | 609 | 67 | 1747 | 10 | 0 |
| *Axud1* | 30 | 4 | 75 | 0 | 0 |
| *Cad88C* | 989 | 10 | 295 | 0 | 1 |
| *c-cup* | 23 | 0 | 21 | 0 | 0 |
| *CG10635* | 619 | 2 | 14 | 0 | 0 |
| *CG11498* | 1183 | 11 | 279 | 0 | 0 |
| *CG11723* | 27 | 3 | 34 | 0 | 0 |
| *CG12674* | 27 | 2 | 35 | 0 | 0 |
| *CG13562* | 534 | 3 | 48 | 1 | 0 |
| *CG13716* | 609 | 1 | 12 | 0 | 0 |
| *CG14853* | 989 | 6 | 273 | 0 | 0 |
| *CG15382* | 27 | 1 | 14 | 0 | 0 |
| *CG15390* | 30 | 0 | 13 | 0 | 0 |
| *CG15529* | 1183 | 4 | 67 | 0 | 0 |
| *CG16995* | 29 | 3 | 42 | 1 | 0 |
| *CG17234* | 29 | 1 | 3 | 0 | 0 |
| *CG17237* | 29 | 0 | 19 | 0 | 0 |
| *CG17239* | 29 | 2 | 21 | 0 | 0 |
| *CG17242* | 29 | 2 | 33 | 0 | 0 |
| *CG17646* | 22 | 23 | 501 | 0 | 0 |
| *CG17648* | 22 | 0 | 19 | 0 | 0 |
| *CG17650* | 22 | 2 | 15 | 0 | 0 |
| *CG17652* | 22 | 2 | 31 | 0 | 0 |
| *CG17658* | 534 | 1 | 26 | 1 | 0 |
| *CG17660* | 22 | 4 | 70 | 0 | 0 |
| *CG17712* | 22 | 3 | 36 | 0 | 0 |
| *CG2812* | 534 | 4 | 54 | 1 | 0 |
| *CG2970* | 534 | 1 | 56 | 1 | 0 |
| *CG31028* | 1183 | 5 | 139 | 0 | 0 |
| *CG31029* | 1183 | 2 | 89 | 0 | 0 |
| *CG31030* | 1183 | 5 | 79 | 0 | 0 |
| *CG31437* | 1124 | 1 | 19 | 0 | 0 |
| *CG31664* | 23 | 2 | 74 | 0 | 0 |
| *CG31681* | 29 | 2 | 32 | 0 | 0 |
| *CG31815* | 208 | 0 | 44 | 0 | 0 |
| *CG31933* | 23 | 2 | 54 | 0 | 0 |
| *CG31937* | 22 | 1 | 40 | 0 | 0 |
| *CG31949* | 29 | 0 | 26 | 0 | 0 |
| *CG32022* | 660 | 0 | 13 | 0 | 0 |
| *CG32246* | 609 | 2 | 33 | 1 | 0 |
| *CG34049* | 29 | 1 | 66 | 0 | 0 |
| *CG3557* | 30 | 1 | 13 | 0 | 0 |
| *CG3597* | 30 | 1 | 24 | 0 | 0 |
| *CG3609* | 30 | 1 | 54 | 0 | 0 |
| *CG3735* | 534 | 2 | 61 | 1 | 0 |
| *CG4168* | 190/191 | 21 | 784 | 12 | 0 |
| *CG42540* | 609 | 33 | 855 | 0 | 0 |
| *CG4259* | 27 | 1 | 24 | 0 | 0 |
| *CG42658* | 29 | 0 | 29 | 0 | 0 |
| *CG4267* | 29 | 4 | 83 | 0 | 0 |
| *CG4270* | 29 | 3 | 27 | 0 | 0 |
| *CG4271* | 29 | 0 | 8 | 0 | 0 |
| *CG43230* | 191 | 1 | 27 | 1 | 0 |
| *CG43750* | 30 | 3 | 61 | 0 | 0 |
| *CG43880* | 651 | 0 | 10 | 0 | 0 |
| *CG43965* | 660 | 0 | 3 | 0 | 0 |
| *CG44094* | 989 | 0 | 5 | 0 | 0 |
| *CG45072* | 1183 | 3 | 54 | 0 | 0 |
| *CG45073* | 1183 | 0 | 39 | 0 | 0 |
| *CG4631* | 208 | 4 | 97 | 0 | 0 |
| *CG4882* | 534 | 2 | 23 | 1 | 0 |
| *CG5339* | 534 | 0 | 15 | 0 | 0 |
| *CG5597* | 534 | 1 | 33 | 0 | 0 |
| *CG6511* | 660 | 0 | 91 | 0 | 0 |
| *CG7886* | 989 | 13 | 367 | 0 | 1 |
| *CG7987* | 989 | 2 | 65 | 0 | 0 |
| *CG8038* | 651 | 1 | 14 | 0 | 0 |
| *CG8042* | 651 | 3 | 57 | 0 | 0 |
| *CG8209* | 651 | 1 | 19 | 0 | 0 |
| *CG9870* | 30 | 3 | 71 | 0 | 0 |
| *CG9967* | 29/30 | 45 | 1225 | 0 | 1 |
| *chinmo* | 22 | 44 | 1142 | 0 | 2 |
| *Cp18* | 660 | 0 | 18 | 0 | 0 |
| *cpb* | 22 | 4 | 33 | 0 | 0 |
| *CR42859* | 29 | 2 | 44 | 0 | 0 |
| *CR43357* | 191 | 0 | 32 | 0 | 0 |
| *CR43682* | 191 | 2 | 57 | 0 | 0 |
| *CR43753* | 29 | 6 | 213 | 0 | 0 |
| *CR43754* | 29 | 0 | 44 | 0 | 0 |
| *CR43853* | 190 | 0 | 21 | 0 | 0 |
| *CR43854* | 190 | 0 | 11 | 0 | 0 |
| *CR44055* | 27 | 1 | 32 | 0 | 0 |
| *CR44073* | 23 | 1 | 7 | 0 | 0 |
| *CR44151* | 208 | 0 | 3 | 0 | 0 |
| *CR44196* | 191 | 1 | 7 | 0 | 0 |
| *CR44515* | 619 | 0 | 20 | 0 | 0 |
| *CR44516* | 619 | 0 | 7 | 0 | 0 |
| *CR44526* | 660 | 1 | 23 | 0 | 0 |
| *CR44706* | 190 | 0 | 16 | 0 | 0 |
| *CR44770* | 190 | 2 | 13 | 1 | 0 |
| *CR44771* | 190 | 0 | 15 | 0 | 0 |
| *CR44787* | 29 | 0 | 13 | 0 | 0 |
| *CR44788* | 29 | 0 | 10 | 0 | 0 |
| *CR44806* | 534 | 0 | 12 | 0 | 0 |
| *CR44808* | 27 | 0 | 4 | 0 | 0 |
| *CR44976* | 22 | 2 | 16 | 0 | 0 |
| *CR44982* | 30 | 0 | 7 | 0 | 0 |
| *CR45438* | 609 | 0 | 15 | 0 | 0 |
| *CR45743* | 619 | 0 | 12 | 0 | 0 |
| *CR45926* | 534 | 1 | 26 | 0 | 0 |
| *CR46082* | 1183 | 6 | 90 | 0 | 0 |
| *CR46112* | 1183 | 1 | 18 | 0 | 0 |
| *CR46153* | 989 | 0 | 1 | 0 | 0 |
| *Cul3* | 191 | 2 | 90 | 1 | 0 |
| *dao* | 191 | 5 | 107 | 2 | 0 |
| *DCP1* | 534 | 0 | 11 | 0 | 0 |
| *DIP-delta* | 619 | 24 | 526 | 3 | 0 |
| *DNA-ligI* | 534 | 4 | 40 | 1 | 0 |
| *dpr3* | 27 | 42 | 1095 | 10 | 0 |
| *Eno* | 22 | 6 | 115 | 0 | 0 |
| *Eogt* | 30 | 1 | 29 | 0 | 0 |
| *exex* | 651 | 3 | 115 | 0 | 0 |
| *eys* | 29/30 | 43 | 1190 | 0 | 1 |
| *frtz* | 22 | 6 | 114 | 0 | 0 |
| *fzr2* | 534 | 1 | 20 | 1 | 0 |
| *Galphas* | 534 | 2 | 89 | 1 | 0 |
| ***GlyP*** | 27 | 4 | 99 | 1 | 0 |
| *Gr22a* | 23 | 4 | 61 | 0 | 0 |
| *Gr22b* | 23 | 4 | 32 | 1 | 0 |
| *Gr22c* | 23 | 1 | 44 | 1 | 0 |
| *Gr22d* | 23 | 1 | 42 | 0 | 0 |
| *Gr22e* | 23 | 0 | 21 | 0 | 0 |
| *Gr22f* | 22 | 3 | 34 | 0 | 0 |
| *h* | 660 | 3 | 59 | 0 | 0 |
| ***HDAC1*** | 609 | 4 | 76 | 0 | 1 |
| *His4r* | 989 | 0 | 11 | 0 | 0 |
| *HP4* | 651 | 1 | 16 | 1 | 0 |
| *Ir64a* | 619 | 8 | 193 | 0 | 1 |
| *kcc* | 534 | 6 | 195 | 2 | 0 |
| *ken* | 534 | 4 | 31 | 4 | 0 |
| *l(2)35Cc* | 191 | 1 | 21 | 1 | 0 |
| *l(3)L1231* | 989 | 1 | 159 | 0 | 0 |
| *Lpt* | 534 | 4 | 113 | 1 | 1 |
| *Membrin* | 619 | 0 | 16 | 0 | 0 |
| *mir-2280* | 23 | 0 | 1 | 0 | 0 |
| *Mlc2* | 1183 | 3 | 55 | 0 | 0 |
| *mRpL48* | 22 | 2 | 33 | 0 | 0 |
| *Muc96D* | 1124 | 1 | 13 | 0 | 0 |
| *Nap1* | 534 | 1 | 36 | 0 | 0 |
| *nmo* | 651 | 74 | 2128 | 2 | 0 |
| *Orcokinin* | 534 | 3 | 43 | 1 | 0 |
| *Pex7* | 660 | 5 | 136 | 1 | 0 |
| *PHDP* | 534 | 2 | 19 | 0 | 0 |
| *Pol32* | 191 | 3 | 25 | 0 | 0 |
| *Ppi1* | 1183 | 4 | 91 | 0 | 0 |
| *put* | 989 | 4 | 91 | 0 | 0 |
| *Rab5* | 30 | 2 | 117 | 0 | 0 |
| ***Rim2*** | 22 | 14 | 247 | 1 | 0 |
| *RNaseX25* | 651 | 1 | 29 | 0 | 0 |
| *robl22E* | 29 | 1 | 20 | 0 | 0 |
| *Rrp40* | 22 | 2 | 24 | 0 | 0 |
| *Send1* | 29 | 0 | 18 | 0 | 0 |
| *Ser12* | 29 | 1 | 8 | 0 | 0 |
| *SERCA* | 534 | 6 | 153 | 2 | 0 |
| *Sfp35C* | 191 | 0 | 7 | 0 | 0 |
| *sima* | 1183 | 81 | 1865 | 1 | 3 |
| *Src64B* | 609 | 38 | 917 | 3 | 0 |
| *Srp9* | 651 | 3 | 25 | 0 | 0 |
| *SrpRbeta* | 660 | 0 | 41 | 0 | 0 |
| *stumps* | 989 | 28 | 803 | 1 | 0 |
| *syd* | 651 | 10 | 224 | 0 | 0 |
| *Taldo* | 534 | 4 | 33 | 1 | 0 |
| *TBCD* | 27 | 4 | 77 | 1 | 0 |
| *Tengl1* | 29 | 1 | 22 | 0 | 0 |
| *tho2* | 27 | 4 | 77 | 0 | 0 |
| *TM4SF* | 534 | 0 | 11 | 0 | 0 |
| ***Tpi*** | 1183 | 1 | 27 | 0 | 0 |
| *tRNA:L:35C* | 190 | 0 | 0 | 0 | 0 |
| *UK114* | 191 | 0 | 22 | 0 | 0 |
| *Upf3* | 534 | 0 | 41 | 0 | 0 |
| *VGlut* | 30 | 21 | 514 | 2 | 0 |
| *wry* | 23 | 30 | 650 | 0 | 0 |
| *yuri* | 191 | 6 | 138 | 2 | 0 |
| *ZnT35C* | 191 | 19 | 476 | 5 | 0 |
