# Supplementary material for: Identification of novel genes associated with longevity in Drosophila melanogaster - a computational approach
Source: Aging (Albany NY). 2019 Dec 3;11(23):11244–67. doi: 10.18632/aging.102527 (PMC6932890; doi:10.18632/aging.102527)
Supplement: Supplementary Table 3 [file aging-11-102527-s004..docx]

**Supplementary Table 3. Nodes and interactions comprising each cluster of the extended Synthetic GWAS-based networks.**

| Cluster | Bin 1 | Bin 2 | Cluster | Bin 1 | Bin 2 | Cluster | Bin 1 | Bin 2 | Cluster | Bin 1 | Bin 2 |
| --- | --- | --- | --- | --- | --- | --- | --- | --- | --- | --- | --- |
| 0 | 6 | 11 | 22 | 293 | 1212 | 39 | 534 | 1246 | 47 | 659 | 661 |
| 0 | 7 | 11 | 22 | 293 | 1221 | 39 | 534 | 1250 | 47 | 659 | 662 |
| 0 | 8 | 11 | 22 | 294 | 295 | 39 | 534 | 1255 | 47 | 659 | 663 |
| 0 | 8 | 12 | 22 | 294 | 296 | 39 | 535 | 536 | 47 | 659 | 664 |
| 0 | 9 | 11 | 22 | 294 | 300 | 39 | 535 | 537 | 47 | 659 | 1430 |
| 0 | 9 | 12 | 22 | 294 | 301 | 39 | 535 | 555 | 47 | 660 | 661 |
| 0 | 10 | 11 | 22 | 294 | 1210 | 39 | 535 | 574 | 47 | 660 | 662 |
| 0 | 10 | 12 | 22 | 294 | 1215 | 39 | 535 | 589 | 47 | 660 | 663 |
| 0 | 10 | 13 | 22 | 294 | 1219 | 39 | 535 | 590 | 47 | 660 | 664 |
| 0 | 11 | 12 | 22 | 294 | 1223 | 39 | 535 | 592 | 47 | 660 | 1324 |
| 0 | 11 | 13 | 22 | 295 | 296 | 39 | 535 | 603 | 47 | 660 | 1367 |
| 0 | 11 | 14 | 22 | 295 | 300 | 39 | 535 | 1064 | 48 | 635 | 668 |
| 0 | 11 | 15 | 22 | 295 | 301 | 39 | 535 | 1143 | 48 | 665 | 667 |
| 0 | 11 | 1035 | 22 | 295 | 302 | 39 | 535 | 1147 | 48 | 665 | 668 |
| 0 | 11 | 1090 | 22 | 295 | 303 | 39 | 535 | 1170 | 48 | 666 | 667 |
| 0 | 11 | 1135 | 22 | 295 | 1209 | 39 | 535 | 1202 | 48 | 666 | 668 |
| 0 | 11 | 1162 | 22 | 295 | 1210 | 39 | 535 | 1239 | 48 | 667 | 668 |
| 0 | 11 | 1199 | 22 | 295 | 1212 | 39 | 535 | 1240 | 48 | 668 | 669 |
| 0 | 12 | 13 | 22 | 295 | 1215 | 39 | 535 | 1246 | 49 | 67 | 673 |
| 0 | 12 | 14 | 22 | 295 | 1216 | 39 | 535 | 1250 | 49 | 67 | 675 |
| 0 | 12 | 1150 | 22 | 295 | 1219 | 39 | 535 | 1280 | 49 | 76 | 673 |
| 0 | 13 | 14 | 22 | 295 | 1222 | 39 | 535 | 1297 | 49 | 514 | 674 |
| 0 | 14 | 15 | 22 | 295 | 1223 | 39 | 535 | 1395 | 49 | 670 | 673 |
| 0 | 15 | 693 | 22 | 296 | 300 | 39 | 535 | 1426 | 49 | 671 | 673 |
| 0 | 15 | 964 | 22 | 296 | 301 | 39 | 535 | 1429 | 49 | 671 | 674 |
| 0 | 15 | 1056 | 22 | 296 | 746 | 39 | 536 | 537 | 49 | 672 | 673 |
| 0 | 15 | 1149 | 22 | 296 | 1210 | 39 | 536 | 555 | 49 | 672 | 674 |
| 0 | 15 | 1199 | 22 | 296 | 1215 | 39 | 536 | 574 | 49 | 672 | 675 |
| 0 | 15 | 1321 | 22 | 296 | 1216 | 39 | 536 | 581 | 49 | 673 | 674 |
| 1 | 16 | 17 | 22 | 296 | 1219 | 39 | 536 | 584 | 49 | 673 | 675 |
| 1 | 16 | 18 | 22 | 296 | 1221 | 39 | 536 | 590 | 49 | 674 | 675 |
| 1 | 16 | 19 | 22 | 296 | 1222 | 39 | 536 | 592 | 49 | 674 | 676 |
| 1 | 16 | 20 | 22 | 296 | 1223 | 39 | 536 | 603 | 49 | 674 | 677 |
| 1 | 17 | 18 | 22 | 297 | 298 | 39 | 536 | 1064 | 49 | 674 | 678 |
| 1 | 17 | 19 | 22 | 297 | 299 | 39 | 536 | 1143 | 49 | 674 | 679 |
| 1 | 17 | 20 | 22 | 297 | 300 | 39 | 536 | 1147 | 49 | 675 | 676 |
| 1 | 17 | 1148 | 22 | 298 | 299 | 39 | 536 | 1170 | 49 | 675 | 677 |
| 2 | 21 | 22 | 22 | 298 | 300 | 39 | 536 | 1202 | 49 | 675 | 678 |
| 2 | 22 | 23 | 22 | 298 | 301 | 39 | 536 | 1255 | 49 | 675 | 679 |
| 2 | 22 | 24 | 22 | 298 | 305 | 39 | 537 | 555 | 50 | 697 | 701 |
| 2 | 22 | 25 | 22 | 298 | 1210 | 39 | 537 | 574 | 50 | 698 | 700 |
| 2 | 22 | 1151 | 22 | 298 | 1222 | 39 | 537 | 603 | 50 | 698 | 701 |
| 2 | 22 | 1411 | 22 | 299 | 300 | 39 | 537 | 947 | 50 | 699 | 700 |
| 2 | 23 | 24 | 22 | 300 | 301 | 39 | 537 | 1147 | 50 | 699 | 701 |
| 2 | 23 | 25 | 22 | 300 | 302 | 39 | 537 | 1170 | 50 | 700 | 701 |
| 2 | 24 | 25 | 22 | 300 | 303 | 39 | 537 | 1202 | 50 | 700 | 702 |
| 2 | 24 | 26 | 22 | 300 | 304 | 39 | 537 | 1239 | 50 | 700 | 703 |
| 2 | 24 | 1068 | 22 | 300 | 1209 | 40 | 538 | 539 | 50 | 701 | 702 |
| 2 | 25 | 26 | 22 | 300 | 1210 | 40 | 538 | 540 | 50 | 701 | 703 |
| 2 | 25 | 27 | 22 | 300 | 1212 | 40 | 538 | 541 | 50 | 701 | 704 |
| 2 | 25 | 28 | 22 | 300 | 1215 | 40 | 538 | 542 | 51 | 718 | 720 |
| 2 | 25 | 1151 | 22 | 300 | 1216 | 40 | 538 | 575 | 51 | 719 | 720 |
| 2 | 26 | 27 | 22 | 300 | 1219 | 40 | 538 | 1175 | 51 | 719 | 721 |
| 2 | 26 | 28 | 22 | 300 | 1221 | 40 | 538 | 1196 | 51 | 719 | 722 |
| 2 | 27 | 28 | 22 | 300 | 1222 | 40 | 538 | 1251 | 51 | 719 | 723 |
| 2 | 28 | 582 | 22 | 300 | 1223 | 40 | 539 | 540 | 51 | 720 | 721 |
| 2 | 28 | 742 | 22 | 301 | 302 | 40 | 539 | 541 | 51 | 720 | 722 |
| 2 | 28 | 1388 | 22 | 301 | 303 | 40 | 539 | 542 | 51 | 720 | 723 |
| 3 | 29 | 30 | 22 | 301 | 304 | 40 | 539 | 564 | 51 | 720 | 724 |
| 3 | 29 | 31 | 22 | 301 | 1210 | 40 | 539 | 575 | 51 | 720 | 725 |
| 3 | 29 | 32 | 22 | 301 | 1222 | 40 | 539 | 1137 | 51 | 720 | 726 |
| 3 | 29 | 33 | 22 | 301 | 1223 | 40 | 540 | 541 | 51 | 720 | 1326 |
| 3 | 29 | 34 | 22 | 302 | 303 | 40 | 540 | 542 | 51 | 721 | 722 |
| 3 | 30 | 31 | 22 | 302 | 304 | 40 | 540 | 543 | 51 | 721 | 723 |
| 3 | 30 | 32 | 22 | 302 | 307 | 40 | 540 | 544 | 51 | 721 | 724 |
| 3 | 30 | 33 | 22 | 302 | 1209 | 40 | 540 | 564 | 51 | 721 | 725 |
| 3 | 30 | 34 | 22 | 302 | 1210 | 40 | 540 | 1008 | 51 | 721 | 1326 |
| 3 | 30 | 1036 | 22 | 302 | 1215 | 40 | 540 | 1137 | 51 | 722 | 723 |
| 3 | 30 | 1453 | 22 | 302 | 1219 | 40 | 540 | 1196 | 51 | 722 | 724 |
| 3 | 31 | 32 | 22 | 302 | 1221 | 40 | 541 | 542 | 51 | 722 | 725 |
| 3 | 31 | 33 | 22 | 302 | 1222 | 40 | 541 | 543 | 51 | 722 | 1326 |
| 3 | 31 | 34 | 22 | 302 | 1223 | 40 | 541 | 544 | 51 | 723 | 724 |
| 3 | 31 | 35 | 22 | 303 | 304 | 40 | 541 | 564 | 51 | 723 | 725 |
| 3 | 31 | 36 | 22 | 303 | 305 | 40 | 541 | 565 | 51 | 723 | 1326 |
| 4 | 89 | 93 | 22 | 303 | 307 | 40 | 541 | 1039 | 51 | 724 | 725 |
| 4 | 90 | 93 | 22 | 303 | 1209 | 40 | 541 | 1175 | 51 | 724 | 1326 |
| 4 | 91 | 93 | 22 | 303 | 1210 | 40 | 541 | 1196 | 51 | 725 | 726 |
| 4 | 92 | 93 | 22 | 303 | 1212 | 40 | 542 | 543 | 51 | 726 | 727 |
| 4 | 93 | 94 | 22 | 303 | 1215 | 40 | 542 | 544 | 51 | 726 | 1326 |
| 4 | 93 | 95 | 22 | 303 | 1216 | 40 | 542 | 564 | 52 | 728 | 732 |
| 4 | 93 | 97 | 22 | 303 | 1219 | 40 | 542 | 575 | 52 | 728 | 733 |
| 4 | 93 | 1319 | 22 | 303 | 1222 | 40 | 542 | 1251 | 52 | 729 | 732 |
| 5 | 124 | 128 | 22 | 303 | 1223 | 40 | 542 | 1393 | 52 | 729 | 733 |
| 5 | 125 | 128 | 22 | 304 | 305 | 40 | 542 | 1448 | 52 | 730 | 732 |
| 5 | 126 | 128 | 22 | 304 | 306 | 40 | 543 | 544 | 52 | 730 | 733 |
| 5 | 127 | 128 | 22 | 304 | 307 | 40 | 543 | 1069 | 52 | 731 | 732 |
| 5 | 128 | 129 | 22 | 304 | 844 | 40 | 543 | 1137 | 52 | 731 | 733 |
| 5 | 128 | 130 | 22 | 304 | 871 | 40 | 543 | 1175 | 52 | 732 | 733 |
| 5 | 128 | 131 | 22 | 304 | 1209 | 40 | 544 | 564 | 52 | 732 | 734 |
| 5 | 128 | 711 | 22 | 304 | 1210 | 40 | 544 | 565 | 52 | 732 | 735 |
| 5 | 128 | 740 | 22 | 304 | 1215 | 40 | 544 | 999 | 52 | 733 | 734 |
| 5 | 128 | 929 | 22 | 304 | 1216 | 40 | 544 | 1155 | 52 | 733 | 735 |
| 6 | 140 | 144 | 22 | 304 | 1222 | 40 | 544 | 1196 | 53 | 319 | 753 |
| 6 | 141 | 144 | 22 | 304 | 1223 | 41 | 545 | 546 | 53 | 751 | 753 |
| 6 | 142 | 144 | 22 | 305 | 306 | 41 | 545 | 547 | 53 | 752 | 753 |
| 6 | 143 | 144 | 22 | 305 | 307 | 41 | 545 | 562 | 53 | 753 | 754 |
| 6 | 144 | 145 | 22 | 305 | 1210 | 41 | 545 | 567 | 53 | 753 | 755 |
| 6 | 144 | 146 | 22 | 305 | 1215 | 41 | 545 | 568 | 53 | 753 | 756 |
| 6 | 144 | 147 | 22 | 305 | 1216 | 41 | 545 | 571 | 53 | 753 | 1249 |
| 6 | 144 | 148 | 22 | 305 | 1221 | 41 | 545 | 572 | 53 | 755 | 758 |
| 7 | 163 | 165 | 22 | 305 | 1222 | 41 | 545 | 573 | 53 | 756 | 758 |
| 7 | 163 | 166 | 22 | 305 | 1223 | 41 | 545 | 594 | 53 | 756 | 759 |
| 7 | 164 | 165 | 23 | 330 | 334 | 41 | 545 | 601 | 53 | 757 | 758 |
| 7 | 165 | 166 | 23 | 331 | 334 | 41 | 545 | 643 | 53 | 757 | 759 |
| 7 | 165 | 167 | 23 | 332 | 334 | 41 | 545 | 690 | 53 | 758 | 759 |
| 7 | 165 | 168 | 23 | 333 | 334 | 41 | 545 | 1088 | 53 | 759 | 1249 |
| 7 | 165 | 173 | 23 | 334 | 335 | 41 | 545 | 1142 | 54 | 45 | 764 |
| 7 | 165 | 874 | 23 | 334 | 336 | 41 | 545 | 1145 | 54 | 62 | 764 |
| 7 | 165 | 1307 | 23 | 334 | 337 | 41 | 545 | 1163 | 54 | 104 | 760 |
| 7 | 166 | 167 | 23 | 334 | 749 | 41 | 545 | 1164 | 54 | 104 | 764 |
| 7 | 166 | 168 | 24 | 338 | 342 | 41 | 545 | 1171 | 54 | 109 | 760 |
| 7 | 166 | 173 | 24 | 338 | 343 | 41 | 545 | 1172 | 54 | 109 | 764 |
| 7 | 167 | 168 | 24 | 339 | 342 | 41 | 545 | 1176 | 54 | 136 | 761 |
| 7 | 167 | 169 | 24 | 340 | 342 | 41 | 545 | 1194 | 54 | 138 | 764 |
| 7 | 168 | 169 | 24 | 340 | 343 | 41 | 545 | 1197 | 54 | 151 | 764 |
| 7 | 168 | 170 | 24 | 341 | 342 | 41 | 545 | 1203 | 54 | 318 | 764 |
| 7 | 168 | 172 | 24 | 341 | 343 | 41 | 545 | 1237 | 54 | 375 | 760 |
| 7 | 168 | 173 | 24 | 341 | 344 | 41 | 546 | 547 | 54 | 378 | 760 |
| 7 | 169 | 170 | 24 | 342 | 343 | 41 | 546 | 567 | 54 | 392 | 764 |
| 7 | 169 | 171 | 24 | 342 | 344 | 41 | 546 | 568 | 54 | 475 | 764 |
| 7 | 169 | 172 | 24 | 343 | 344 | 41 | 546 | 571 | 54 | 515 | 760 |
| 7 | 169 | 173 | 24 | 343 | 345 | 41 | 546 | 572 | 54 | 760 | 761 |
| 7 | 170 | 171 | 24 | 343 | 346 | 41 | 546 | 573 | 54 | 760 | 762 |
| 7 | 170 | 172 | 24 | 343 | 908 | 41 | 546 | 602 | 54 | 760 | 763 |
| 7 | 171 | 172 | 24 | 343 | 978 | 41 | 546 | 774 | 54 | 760 | 764 |
| 7 | 171 | 173 | 24 | 344 | 345 | 41 | 546 | 1066 | 54 | 761 | 762 |
| 7 | 172 | 173 | 24 | 344 | 346 | 41 | 546 | 1088 | 54 | 761 | 763 |
| 7 | 173 | 691 | 24 | 344 | 910 | 41 | 546 | 1117 | 54 | 761 | 764 |
| 7 | 173 | 1034 | 24 | 344 | 913 | 41 | 546 | 1119 | 54 | 761 | 1213 |
| 8 | 174 | 175 | 24 | 344 | 1009 | 41 | 546 | 1142 | 54 | 762 | 763 |
| 8 | 174 | 176 | 24 | 345 | 346 | 41 | 546 | 1145 | 54 | 762 | 764 |
| 8 | 174 | 177 | 24 | 345 | 423 | 41 | 546 | 1171 | 54 | 763 | 764 |
| 8 | 174 | 178 | 24 | 345 | 424 | 41 | 546 | 1172 | 54 | 763 | 1213 |
| 8 | 174 | 179 | 25 | 347 | 348 | 41 | 546 | 1176 | 54 | 764 | 770 |
| 8 | 175 | 176 | 25 | 347 | 349 | 41 | 546 | 1194 | 54 | 764 | 1213 |
| 8 | 175 | 177 | 25 | 347 | 350 | 41 | 546 | 1203 | 54 | 764 | 1370 |
| 8 | 175 | 178 | 25 | 348 | 349 | 41 | 546 | 1234 | 55 | 765 | 766 |
| 8 | 175 | 179 | 25 | 348 | 350 | 41 | 546 | 1279 | 55 | 765 | 767 |
| 8 | 176 | 177 | 25 | 349 | 350 | 41 | 546 | 1308 | 55 | 765 | 768 |
| 8 | 176 | 178 | 25 | 349 | 351 | 41 | 547 | 562 | 55 | 765 | 769 |
| 8 | 176 | 179 | 25 | 350 | 351 | 41 | 547 | 567 | 55 | 766 | 767 |
| 8 | 177 | 178 | 25 | 350 | 352 | 41 | 547 | 568 | 55 | 766 | 768 |
| 8 | 177 | 179 | 25 | 350 | 353 | 41 | 547 | 571 | 55 | 766 | 769 |
| 8 | 178 | 179 | 25 | 351 | 353 | 41 | 547 | 572 | 55 | 767 | 768 |
| 8 | 178 | 1364 | 25 | 351 | 354 | 41 | 547 | 573 | 55 | 767 | 769 |
| 9 | 180 | 181 | 25 | 352 | 353 | 41 | 547 | 594 | 56 | 787 | 790 |
| 9 | 180 | 182 | 25 | 352 | 354 | 41 | 547 | 600 | 56 | 788 | 790 |
| 9 | 180 | 183 | 25 | 353 | 354 | 41 | 547 | 601 | 56 | 788 | 792 |
| 9 | 181 | 182 | 25 | 353 | 694 | 41 | 547 | 602 | 56 | 789 | 790 |
| 9 | 181 | 183 | 25 | 353 | 1489 | 41 | 547 | 643 | 56 | 789 | 791 |
| 9 | 182 | 183 | 26 | 355 | 356 | 41 | 547 | 690 | 56 | 789 | 792 |
| 10 | 184 | 187 | 26 | 355 | 357 | 41 | 547 | 1063 | 56 | 790 | 791 |
| 10 | 184 | 188 | 26 | 355 | 358 | 41 | 547 | 1117 | 56 | 790 | 792 |
| 10 | 185 | 187 | 26 | 356 | 357 | 41 | 547 | 1119 | 56 | 791 | 792 |
| 10 | 185 | 188 | 26 | 356 | 358 | 41 | 547 | 1138 | 56 | 791 | 793 |
| 10 | 186 | 187 | 26 | 357 | 358 | 41 | 547 | 1142 | 56 | 791 | 794 |
| 10 | 186 | 188 | 26 | 358 | 359 | 41 | 547 | 1145 | 56 | 791 | 795 |
| 10 | 187 | 188 | 26 | 358 | 360 | 41 | 547 | 1171 | 56 | 792 | 793 |
| 10 | 187 | 189 | 26 | 358 | 361 | 41 | 547 | 1172 | 56 | 792 | 794 |
| 10 | 187 | 190 | 26 | 358 | 974 | 41 | 547 | 1174 | 56 | 792 | 795 |
| 10 | 188 | 189 | 26 | 359 | 360 | 41 | 547 | 1176 | 56 | 792 | 796 |
| 10 | 188 | 190 | 26 | 359 | 361 | 41 | 547 | 1197 | 57 | 106 | 799 |
| 10 | 188 | 191 | 26 | 359 | 915 | 41 | 547 | 1203 | 57 | 797 | 798 |
| 10 | 189 | 190 | 26 | 360 | 361 | 41 | 547 | 1234 | 57 | 797 | 799 |
| 10 | 189 | 191 | 26 | 360 | 682 | 41 | 547 | 1236 | 57 | 798 | 799 |
| 10 | 189 | 192 | 26 | 360 | 868 | 41 | 547 | 1247 | 57 | 799 | 800 |
| 10 | 189 | 193 | 26 | 360 | 928 | 41 | 547 | 1265 | 58 | 52 | 805 |
| 10 | 189 | 194 | 26 | 361 | 772 | 41 | 547 | 1274 | 58 | 121 | 805 |
| 10 | 190 | 191 | 26 | 361 | 928 | 41 | 547 | 1323 | 58 | 328 | 805 |
| 10 | 190 | 192 | 26 | 361 | 1019 | 41 | 547 | 1428 | 58 | 801 | 805 |
| 10 | 190 | 193 | 26 | 361 | 1091 | 42 | 548 | 549 | 58 | 802 | 805 |
| 10 | 190 | 194 | 27 | 362 | 363 | 42 | 548 | 550 | 58 | 803 | 805 |
| 10 | 190 | 195 | 27 | 362 | 364 | 42 | 548 | 552 | 58 | 804 | 805 |
| 10 | 191 | 192 | 27 | 362 | 365 | 42 | 548 | 553 | 58 | 805 | 806 |
| 10 | 191 | 193 | 27 | 362 | 366 | 42 | 548 | 554 | 58 | 805 | 807 |
| 10 | 191 | 194 | 27 | 362 | 708 | 42 | 548 | 556 | 59 | 310 | 810 |
| 10 | 191 | 195 | 27 | 363 | 364 | 42 | 548 | 557 | 59 | 808 | 810 |
| 11 | 198 | 203 | 27 | 363 | 365 | 42 | 548 | 558 | 59 | 809 | 810 |
| 11 | 199 | 203 | 27 | 364 | 365 | 42 | 548 | 560 | 59 | 810 | 811 |
| 11 | 200 | 203 | 27 | 364 | 366 | 42 | 548 | 561 | 59 | 810 | 812 |
| 11 | 201 | 203 | 27 | 364 | 877 | 42 | 548 | 566 | 59 | 810 | 813 |
| 11 | 202 | 203 | 27 | 364 | 896 | 42 | 548 | 569 | 59 | 810 | 814 |
| 11 | 203 | 204 | 27 | 364 | 1043 | 42 | 548 | 577 | 59 | 810 | 815 |
| 11 | 204 | 205 | 27 | 364 | 1076 | 42 | 548 | 580 | 60 | 313 | 822 |
| 11 | 204 | 210 | 27 | 364 | 1261 | 42 | 548 | 583 | 60 | 385 | 822 |
| 11 | 205 | 206 | 27 | 365 | 366 | 42 | 548 | 595 | 60 | 476 | 822 |
| 11 | 205 | 207 | 27 | 366 | 367 | 42 | 548 | 596 | 60 | 818 | 822 |
| 11 | 205 | 209 | 27 | 366 | 368 | 42 | 548 | 597 | 60 | 819 | 822 |
| 11 | 206 | 207 | 27 | 366 | 931 | 42 | 548 | 598 | 60 | 820 | 822 |
| 11 | 206 | 208 | 27 | 366 | 933 | 42 | 548 | 780 | 60 | 821 | 822 |
| 11 | 206 | 209 | 27 | 366 | 1007 | 42 | 548 | 1040 | 60 | 822 | 823 |
| 11 | 207 | 208 | 27 | 366 | 1060 | 42 | 548 | 1074 | 60 | 822 | 824 |
| 11 | 207 | 209 | 27 | 366 | 1443 | 42 | 548 | 1075 | 60 | 822 | 825 |
| 11 | 208 | 209 | 27 | 367 | 368 | 42 | 548 | 1156 | 60 | 822 | 826 |
| 11 | 209 | 210 | 27 | 367 | 369 | 42 | 548 | 1173 | 60 | 822 | 827 |
| 11 | 209 | 211 | 27 | 367 | 370 | 42 | 548 | 1204 | 60 | 822 | 1218 |
| 11 | 210 | 211 | 27 | 367 | 817 | 42 | 548 | 1205 | 61 | 842 | 846 |
| 11 | 210 | 578 | 27 | 367 | 1298 | 42 | 548 | 1206 | 61 | 842 | 848 |
| 11 | 210 | 916 | 27 | 368 | 369 | 42 | 548 | 1207 | 61 | 843 | 846 |
| 11 | 210 | 930 | 27 | 368 | 370 | 42 | 548 | 1224 | 61 | 843 | 848 |
| 11 | 210 | 982 | 27 | 369 | 370 | 42 | 548 | 1226 | 61 | 845 | 846 |
| 11 | 210 | 1431 | 27 | 369 | 371 | 42 | 548 | 1227 | 61 | 845 | 847 |
| 11 | 210 | 1436 | 27 | 369 | 631 | 42 | 548 | 1228 | 61 | 845 | 848 |
| 11 | 211 | 1049 | 27 | 369 | 1046 | 42 | 548 | 1229 | 61 | 846 | 847 |
| 12 | 212 | 213 | 27 | 370 | 371 | 42 | 548 | 1230 | 61 | 846 | 848 |
| 12 | 212 | 214 | 27 | 370 | 1449 | 42 | 548 | 1231 | 61 | 847 | 848 |
| 12 | 212 | 215 | 28 | 386 | 388 | 42 | 548 | 1232 | 61 | 848 | 849 |
| 12 | 212 | 216 | 28 | 386 | 389 | 42 | 548 | 1241 | 61 | 848 | 851 |
| 12 | 212 | 217 | 28 | 387 | 388 | 42 | 548 | 1243 | 61 | 848 | 852 |
| 12 | 212 | 218 | 28 | 387 | 389 | 42 | 548 | 1244 | 62 | 879 | 882 |
| 12 | 213 | 214 | 28 | 388 | 389 | 42 | 548 | 1245 | 62 | 879 | 883 |
| 12 | 213 | 215 | 28 | 388 | 390 | 42 | 548 | 1248 | 62 | 880 | 882 |
| 12 | 213 | 216 | 28 | 389 | 390 | 42 | 548 | 1252 | 62 | 880 | 883 |
| 12 | 213 | 217 | 28 | 389 | 391 | 42 | 548 | 1253 | 62 | 881 | 882 |
| 12 | 214 | 215 | 28 | 389 | 779 | 42 | 548 | 1270 | 62 | 881 | 883 |
| 12 | 214 | 216 | 29 | 393 | 395 | 42 | 548 | 1277 | 62 | 882 | 883 |
| 12 | 214 | 217 | 29 | 393 | 397 | 42 | 548 | 1335 | 62 | 882 | 884 |
| 12 | 215 | 216 | 29 | 394 | 395 | 42 | 548 | 1344 | 62 | 882 | 885 |
| 12 | 215 | 217 | 29 | 395 | 396 | 42 | 548 | 1389 | 62 | 882 | 888 |
| 12 | 215 | 218 | 29 | 395 | 397 | 42 | 548 | 1438 | 62 | 883 | 884 |
| 12 | 216 | 217 | 29 | 396 | 397 | 42 | 549 | 550 | 62 | 883 | 885 |
| 12 | 216 | 218 | 29 | 397 | 398 | 42 | 549 | 552 | 62 | 883 | 886 |
| 12 | 217 | 218 | 29 | 397 | 399 | 42 | 549 | 553 | 63 | 919 | 920 |
| 13 | 219 | 220 | 29 | 397 | 400 | 42 | 549 | 554 | 63 | 919 | 922 |
| 13 | 219 | 221 | 29 | 397 | 961 | 42 | 549 | 556 | 63 | 920 | 921 |
| 13 | 219 | 222 | 29 | 397 | 1338 | 42 | 549 | 557 | 63 | 920 | 922 |
| 13 | 219 | 225 | 29 | 399 | 401 | 42 | 549 | 558 | 63 | 920 | 923 |
| 13 | 219 | 227 | 29 | 400 | 401 | 42 | 549 | 560 | 63 | 921 | 922 |
| 13 | 219 | 1485 | 29 | 401 | 402 | 42 | 549 | 561 | 63 | 921 | 923 |
| 13 | 220 | 221 | 29 | 401 | 403 | 42 | 549 | 566 | 63 | 922 | 923 |
| 13 | 220 | 222 | 29 | 401 | 1044 | 42 | 549 | 569 | 63 | 922 | 924 |
| 13 | 220 | 223 | 30 | 404 | 408 | 42 | 549 | 577 | 63 | 922 | 925 |
| 13 | 220 | 224 | 30 | 404 | 409 | 42 | 549 | 579 | 63 | 923 | 924 |
| 13 | 220 | 225 | 30 | 405 | 408 | 42 | 549 | 580 | 63 | 923 | 925 |
| 13 | 220 | 226 | 30 | 406 | 408 | 42 | 549 | 595 | 64 | 311 | 989 |
| 13 | 221 | 222 | 30 | 407 | 408 | 42 | 549 | 597 | 64 | 511 | 989 |
| 13 | 221 | 223 | 30 | 408 | 409 | 42 | 549 | 598 | 64 | 983 | 989 |
| 13 | 221 | 224 | 30 | 408 | 1000 | 42 | 549 | 1052 | 64 | 985 | 988 |
| 13 | 221 | 225 | 30 | 408 | 1011 | 42 | 549 | 1074 | 64 | 985 | 989 |
| 13 | 221 | 226 | 30 | 409 | 410 | 42 | 549 | 1160 | 64 | 986 | 988 |
| 13 | 222 | 223 | 30 | 409 | 586 | 42 | 549 | 1198 | 64 | 986 | 989 |
| 13 | 222 | 224 | 30 | 409 | 979 | 42 | 549 | 1204 | 64 | 987 | 988 |
| 13 | 222 | 225 | 30 | 409 | 1067 | 42 | 549 | 1205 | 64 | 987 | 989 |
| 13 | 222 | 226 | 30 | 409 | 1381 | 42 | 549 | 1206 | 64 | 988 | 989 |
| 13 | 223 | 224 | 30 | 409 | 1424 | 42 | 549 | 1207 | 64 | 988 | 990 |
| 13 | 223 | 225 | 30 | 409 | 1432 | 42 | 549 | 1224 | 64 | 989 | 990 |
| 13 | 223 | 226 | 30 | 409 | 1444 | 42 | 549 | 1225 | 64 | 989 | 991 |
| 13 | 223 | 227 | 30 | 410 | 411 | 42 | 549 | 1226 | 64 | 989 | 992 |
| 13 | 224 | 225 | 30 | 410 | 412 | 42 | 549 | 1227 | 65 | 1020 | 1023 |
| 13 | 224 | 226 | 30 | 410 | 413 | 42 | 549 | 1228 | 65 | 1021 | 1023 |
| 13 | 224 | 227 | 30 | 410 | 771 | 42 | 549 | 1229 | 65 | 1022 | 1023 |
| 13 | 225 | 226 | 30 | 410 | 1055 | 42 | 549 | 1230 | 65 | 1023 | 1024 |
| 13 | 225 | 227 | 30 | 410 | 1071 | 42 | 549 | 1231 | 65 | 1023 | 1025 |
| 13 | 225 | 832 | 30 | 410 | 1259 | 42 | 549 | 1232 | 65 | 1023 | 1026 |
| 13 | 226 | 227 | 30 | 411 | 412 | 42 | 549 | 1248 | 66 | 139 | 1028 |
| 13 | 227 | 680 | 31 | 417 | 419 | 42 | 549 | 1252 | 66 | 1027 | 1028 |
| 14 | 228 | 229 | 31 | 418 | 419 | 42 | 549 | 1253 | 66 | 1028 | 1029 |
| 14 | 228 | 230 | 31 | 418 | 420 | 42 | 549 | 1310 | 66 | 1028 | 1030 |
| 14 | 228 | 231 | 31 | 418 | 421 | 42 | 549 | 1312 | 66 | 1028 | 1031 |
| 14 | 228 | 232 | 31 | 419 | 420 | 42 | 549 | 1315 | 66 | 1028 | 1032 |
| 14 | 229 | 230 | 31 | 419 | 421 | 42 | 549 | 1317 | 66 | 1028 | 1033 |
| 14 | 229 | 231 | 31 | 419 | 1365 | 42 | 549 | 1330 | 67 | 502 | 1082 |
| 14 | 229 | 232 | 31 | 420 | 421 | 42 | 549 | 1332 | 67 | 1078 | 1081 |
| 14 | 229 | 887 | 31 | 420 | 422 | 42 | 549 | 1438 | 67 | 1079 | 1081 |
| 14 | 230 | 231 | 31 | 420 | 1331 | 42 | 550 | 551 | 67 | 1079 | 1082 |
| 14 | 230 | 232 | 31 | 421 | 422 | 42 | 550 | 552 | 67 | 1080 | 1081 |
| 14 | 231 | 232 | 32 | 426 | 429 | 42 | 550 | 553 | 67 | 1080 | 1082 |
| 14 | 231 | 233 | 32 | 427 | 429 | 42 | 550 | 554 | 67 | 1081 | 1082 |
| 14 | 232 | 233 | 32 | 427 | 430 | 42 | 550 | 556 | 67 | 1081 | 1083 |
| 14 | 232 | 936 | 32 | 428 | 429 | 42 | 550 | 557 | 67 | 1081 | 1084 |
| 14 | 233 | 736 | 32 | 428 | 430 | 42 | 550 | 558 | 67 | 1081 | 1085 |
| 15 | 240 | 241 | 32 | 428 | 431 | 42 | 550 | 560 | 67 | 1082 | 1083 |
| 15 | 240 | 242 | 32 | 428 | 432 | 42 | 550 | 561 | 67 | 1082 | 1084 |
| 15 | 240 | 243 | 32 | 429 | 430 | 42 | 550 | 569 | 67 | 1082 | 1085 |
| 15 | 241 | 242 | 32 | 429 | 431 | 42 | 550 | 579 | 67 | 1082 | 1086 |
| 15 | 241 | 243 | 32 | 429 | 432 | 42 | 550 | 980 | 67 | 1082 | 1386 |
| 15 | 241 | 738 | 32 | 429 | 433 | 42 | 550 | 981 | 68 | 42 | 1100 |
| 15 | 242 | 243 | 32 | 430 | 431 | 42 | 550 | 1074 | 68 | 56 | 1098 |
| 15 | 243 | 939 | 32 | 430 | 432 | 42 | 550 | 1075 | 68 | 84 | 1098 |
| 15 | 243 | 1238 | 32 | 430 | 433 | 42 | 550 | 1195 | 68 | 112 | 1098 |
| 16 | 234 | 235 | 32 | 431 | 432 | 42 | 550 | 1198 | 68 | 449 | 1098 |
| 16 | 234 | 236 | 32 | 431 | 433 | 42 | 550 | 1204 | 68 | 1094 | 1097 |
| 16 | 234 | 237 | 32 | 432 | 433 | 42 | 550 | 1205 | 68 | 1095 | 1097 |
| 16 | 234 | 238 | 32 | 433 | 873 | 42 | 550 | 1206 | 68 | 1095 | 1098 |
| 16 | 234 | 932 | 33 | 434 | 435 | 42 | 550 | 1207 | 68 | 1096 | 1097 |
| 16 | 235 | 236 | 33 | 434 | 436 | 42 | 550 | 1224 | 68 | 1096 | 1098 |
| 16 | 235 | 237 | 33 | 434 | 437 | 42 | 550 | 1225 | 68 | 1096 | 1099 |
| 16 | 235 | 238 | 33 | 434 | 1087 | 42 | 550 | 1226 | 68 | 1097 | 1098 |
| 16 | 236 | 237 | 33 | 435 | 436 | 42 | 550 | 1227 | 68 | 1097 | 1099 |
| 16 | 236 | 238 | 33 | 435 | 437 | 42 | 550 | 1228 | 68 | 1097 | 1100 |
| 16 | 236 | 239 | 33 | 435 | 438 | 42 | 550 | 1229 | 68 | 1097 | 1103 |
| 16 | 236 | 1378 | 33 | 435 | 998 | 42 | 550 | 1230 | 68 | 1097 | 1294 |
| 16 | 237 | 238 | 33 | 435 | 1012 | 42 | 550 | 1231 | 68 | 1098 | 1099 |
| 16 | 237 | 239 | 33 | 435 | 1087 | 42 | 550 | 1232 | 68 | 1098 | 1100 |
| 16 | 237 | 710 | 33 | 435 | 1368 | 42 | 550 | 1245 | 68 | 1098 | 1101 |
| 16 | 238 | 239 | 33 | 436 | 437 | 42 | 550 | 1248 | 68 | 1098 | 1102 |
| 16 | 238 | 244 | 33 | 436 | 438 | 42 | 550 | 1252 | 68 | 1098 | 1103 |
| 16 | 238 | 245 | 33 | 437 | 438 | 42 | 550 | 1253 | 68 | 1098 | 1294 |
| 16 | 239 | 244 | 33 | 437 | 439 | 42 | 550 | 1260 | 68 | 1099 | 1100 |
| 16 | 239 | 245 | 33 | 437 | 1087 | 42 | 550 | 1278 | 68 | 1099 | 1101 |
| 16 | 239 | 864 | 33 | 437 | 1254 | 42 | 550 | 1299 | 68 | 1099 | 1102 |
| 16 | 239 | 942 | 33 | 437 | 1445 | 42 | 550 | 1309 | 68 | 1099 | 1103 |
| 16 | 244 | 245 | 33 | 438 | 439 | 42 | 550 | 1312 | 68 | 1100 | 1101 |
| 16 | 244 | 835 | 33 | 438 | 440 | 42 | 550 | 1314 | 68 | 1100 | 1102 |
| 16 | 244 | 897 | 33 | 438 | 441 | 42 | 550 | 1317 | 68 | 1100 | 1103 |
| 16 | 244 | 996 | 33 | 438 | 442 | 42 | 550 | 1318 | 68 | 1100 | 1165 |
| 16 | 244 | 1220 | 33 | 438 | 585 | 42 | 550 | 1344 | 69 | 70 | 1105 |
| 16 | 245 | 859 | 34 | 454 | 456 | 42 | 551 | 552 | 69 | 87 | 1105 |
| 16 | 245 | 917 | 34 | 454 | 457 | 42 | 551 | 553 | 69 | 448 | 1105 |
| 16 | 245 | 969 | 34 | 455 | 456 | 42 | 551 | 554 | 69 | 1104 | 1105 |
| 16 | 245 | 1220 | 34 | 455 | 457 | 42 | 551 | 1204 | 69 | 1105 | 1106 |
| 16 | 245 | 1380 | 34 | 456 | 457 | 42 | 551 | 1205 | 69 | 1105 | 1107 |
| 17 | 246 | 247 | 34 | 456 | 458 | 42 | 551 | 1206 | 70 | 68 | 1111 |
| 17 | 246 | 248 | 34 | 456 | 1089 | 42 | 551 | 1207 | 70 | 102 | 1111 |
| 17 | 247 | 248 | 34 | 457 | 458 | 42 | 551 | 1224 | 70 | 487 | 1111 |
| 17 | 247 | 249 | 34 | 457 | 462 | 42 | 551 | 1225 | 70 | 1108 | 1111 |
| 17 | 247 | 250 | 34 | 457 | 1006 | 42 | 551 | 1226 | 70 | 1109 | 1111 |
| 17 | 247 | 251 | 34 | 457 | 1089 | 42 | 551 | 1227 | 70 | 1110 | 1111 |
| 17 | 248 | 249 | 34 | 457 | 1152 | 42 | 551 | 1228 | 70 | 1111 | 1112 |
| 17 | 248 | 250 | 34 | 457 | 1322 | 43 | 99 | 607 | 70 | 1111 | 1113 |
| 17 | 248 | 251 | 34 | 458 | 459 | 43 | 604 | 607 | 70 | 1111 | 1114 |
| 17 | 248 | 1488 | 34 | 458 | 460 | 43 | 605 | 607 | 70 | 1111 | 1115 |
| 17 | 249 | 250 | 34 | 458 | 461 | 43 | 606 | 607 | 70 | 1111 | 1116 |
| 17 | 249 | 251 | 34 | 458 | 462 | 43 | 607 | 608 | 71 | 507 | 1123 |
| 17 | 250 | 251 | 34 | 458 | 463 | 43 | 607 | 609 | 71 | 513 | 1123 |
| 17 | 251 | 830 | 34 | 458 | 1418 | 43 | 607 | 610 | 71 | 1120 | 1123 |
| 18 | 252 | 253 | 34 | 459 | 463 | 43 | 607 | 611 | 71 | 1121 | 1123 |
| 18 | 252 | 254 | 34 | 460 | 463 | 43 | 608 | 609 | 71 | 1122 | 1123 |
| 18 | 252 | 255 | 34 | 461 | 463 | 43 | 608 | 610 | 71 | 1122 | 1124 |
| 18 | 253 | 254 | 34 | 462 | 463 | 43 | 609 | 610 | 71 | 1122 | 1125 |
| 18 | 254 | 255 | 34 | 462 | 465 | 43 | 609 | 611 | 71 | 1123 | 1124 |
| 18 | 255 | 256 | 34 | 463 | 464 | 43 | 610 | 611 | 71 | 1123 | 1125 |
| 18 | 255 | 257 | 34 | 463 | 465 | 44 | 46 | 621 | 71 | 1123 | 1126 |
| 18 | 255 | 258 | 34 | 463 | 1054 | 44 | 612 | 613 | 71 | 1123 | 1127 |
| 18 | 255 | 259 | 34 | 463 | 1169 | 44 | 612 | 614 | 71 | 1124 | 1125 |
| 18 | 255 | 865 | 34 | 464 | 465 | 44 | 612 | 615 | 71 | 1124 | 1126 |
| 18 | 255 | 867 | 35 | 466 | 467 | 44 | 612 | 616 | 71 | 1125 | 1126 |
| 18 | 256 | 257 | 35 | 466 | 468 | 44 | 613 | 614 | 71 | 1125 | 1127 |
| 18 | 256 | 258 | 35 | 466 | 469 | 44 | 613 | 615 | 72 | 1128 | 1130 |
| 18 | 256 | 259 | 35 | 467 | 468 | 44 | 613 | 616 | 72 | 1128 | 1131 |
| 18 | 257 | 258 | 35 | 467 | 469 | 44 | 613 | 617 | 72 | 1129 | 1130 |
| 18 | 257 | 259 | 35 | 467 | 470 | 44 | 613 | 618 | 72 | 1129 | 1131 |
| 18 | 258 | 259 | 35 | 468 | 469 | 44 | 613 | 619 | 72 | 1130 | 1131 |
| 18 | 258 | 260 | 35 | 468 | 470 | 44 | 614 | 615 | 72 | 1130 | 1132 |
| 18 | 259 | 260 | 35 | 468 | 900 | 44 | 614 | 616 | 72 | 1131 | 1132 |
| 18 | 259 | 865 | 35 | 468 | 1072 | 44 | 614 | 617 | 72 | 1131 | 1133 |
| 18 | 259 | 935 | 35 | 468 | 1144 | 44 | 614 | 618 | 72 | 1131 | 1134 |
| 18 | 260 | 867 | 35 | 468 | 1146 | 44 | 614 | 619 | 73 | 1 | 1180 |
| 19 | 261 | 262 | 35 | 469 | 470 | 44 | 614 | 620 | 73 | 1 | 1182 |
| 19 | 261 | 263 | 35 | 469 | 471 | 44 | 615 | 616 | 73 | 1 | 1183 |
| 19 | 261 | 264 | 35 | 470 | 471 | 44 | 615 | 617 | 73 | 4 | 1180 |
| 19 | 261 | 265 | 35 | 470 | 472 | 44 | 616 | 617 | 73 | 4 | 1182 |
| 19 | 261 | 741 | 35 | 470 | 473 | 44 | 616 | 618 | 73 | 4 | 1183 |
| 19 | 261 | 816 | 35 | 470 | 474 | 44 | 616 | 619 | 73 | 37 | 1182 |
| 19 | 261 | 837 | 35 | 470 | 1072 | 44 | 616 | 621 | 73 | 37 | 1183 |
| 19 | 261 | 862 | 36 | 489 | 490 | 44 | 617 | 618 | 73 | 38 | 1180 |
| 19 | 261 | 876 | 36 | 489 | 491 | 44 | 617 | 619 | 73 | 38 | 1182 |
| 19 | 261 | 878 | 36 | 489 | 493 | 44 | 617 | 620 | 73 | 38 | 1183 |
| 19 | 261 | 1045 | 36 | 490 | 491 | 44 | 618 | 619 | 73 | 47 | 1182 |
| 19 | 262 | 263 | 36 | 490 | 492 | 44 | 618 | 620 | 73 | 55 | 1182 |
| 19 | 262 | 264 | 36 | 490 | 494 | 44 | 618 | 621 | 73 | 65 | 1183 |
| 19 | 262 | 265 | 36 | 490 | 495 | 44 | 619 | 620 | 73 | 74 | 1180 |
| 19 | 262 | 927 | 36 | 491 | 492 | 44 | 619 | 621 | 73 | 75 | 1182 |
| 19 | 262 | 1358 | 36 | 491 | 493 | 44 | 619 | 622 | 73 | 80 | 1182 |
| 19 | 263 | 264 | 36 | 491 | 494 | 44 | 619 | 623 | 73 | 85 | 1183 |
| 19 | 264 | 265 | 36 | 491 | 495 | 44 | 620 | 621 | 73 | 88 | 1183 |
| 19 | 264 | 266 | 36 | 492 | 493 | 44 | 620 | 622 | 73 | 98 | 1183 |
| 19 | 264 | 862 | 36 | 493 | 494 | 44 | 620 | 623 | 73 | 100 | 1182 |
| 19 | 264 | 941 | 36 | 493 | 495 | 44 | 621 | 622 | 73 | 103 | 1182 |
| 19 | 265 | 266 | 36 | 493 | 496 | 44 | 621 | 623 | 73 | 377 | 1183 |
| 19 | 265 | 267 | 36 | 493 | 497 | 45 | 624 | 625 | 73 | 446 | 1183 |
| 19 | 265 | 861 | 36 | 493 | 498 | 45 | 624 | 626 | 73 | 453 | 1180 |
| 19 | 265 | 862 | 37 | 518 | 519 | 45 | 624 | 627 | 73 | 453 | 1183 |
| 19 | 265 | 866 | 37 | 518 | 520 | 45 | 625 | 626 | 73 | 477 | 1183 |
| 19 | 265 | 872 | 37 | 518 | 521 | 45 | 625 | 627 | 73 | 478 | 1180 |
| 19 | 265 | 875 | 37 | 519 | 520 | 45 | 625 | 628 | 73 | 478 | 1182 |
| 19 | 265 | 876 | 37 | 519 | 521 | 45 | 626 | 627 | 73 | 501 | 1182 |
| 19 | 265 | 895 | 37 | 519 | 522 | 45 | 626 | 628 | 73 | 501 | 1183 |
| 19 | 265 | 927 | 37 | 519 | 523 | 45 | 627 | 628 | 73 | 506 | 1180 |
| 19 | 265 | 934 | 37 | 519 | 640 | 45 | 627 | 629 | 73 | 506 | 1182 |
| 19 | 265 | 954 | 37 | 520 | 521 | 46 | 2 | 650 | 73 | 512 | 1183 |
| 19 | 265 | 1211 | 37 | 520 | 522 | 46 | 2 | 651 | 73 | 516 | 1182 |
| 19 | 265 | 1336 | 37 | 520 | 523 | 46 | 2 | 652 | 73 | 1178 | 1180 |
| 19 | 265 | 1469 | 37 | 520 | 524 | 46 | 83 | 652 | 73 | 1178 | 1182 |
| 19 | 266 | 267 | 37 | 520 | 775 | 46 | 380 | 652 | 73 | 1179 | 1180 |
| 19 | 266 | 268 | 37 | 521 | 522 | 46 | 644 | 647 | 73 | 1179 | 1182 |
| 19 | 266 | 778 | 37 | 521 | 523 | 46 | 644 | 648 | 73 | 1180 | 1181 |
| 19 | 266 | 837 | 37 | 521 | 527 | 46 | 645 | 647 | 73 | 1180 | 1182 |
| 19 | 266 | 866 | 37 | 521 | 940 | 46 | 645 | 648 | 73 | 1180 | 1183 |
| 19 | 266 | 875 | 38 | 528 | 530 | 46 | 646 | 647 | 73 | 1181 | 1182 |
| 19 | 266 | 876 | 38 | 529 | 530 | 46 | 646 | 648 | 73 | 1181 | 1183 |
| 19 | 266 | 927 | 38 | 529 | 531 | 46 | 646 | 649 | 73 | 1182 | 1183 |
| 19 | 266 | 977 | 38 | 530 | 531 | 46 | 647 | 648 | 73 | 1182 | 1184 |
| 19 | 266 | 1070 | 38 | 530 | 532 | 46 | 647 | 649 | 73 | 1182 | 1256 |
| 19 | 266 | 1211 | 38 | 530 | 533 | 46 | 648 | 649 | 73 | 1183 | 1184 |
| 19 | 267 | 268 | 38 | 530 | 559 | 46 | 648 | 650 | 73 | 1183 | 1256 |
| 19 | 267 | 689 | 38 | 530 | 951 | 46 | 649 | 650 | 73 | 1183 | 1328 |
| 19 | 267 | 872 | 38 | 530 | 952 | 46 | 649 | 651 | 73 | 1183 | 1329 |
| 19 | 267 | 918 | 38 | 530 | 1077 | 46 | 649 | 1347 | 73 | 1183 | 1366 |
| 19 | 267 | 1211 | 38 | 530 | 1193 | 46 | 650 | 651 | 74 | 5 | 1187 |
| 19 | 268 | 862 | 38 | 530 | 1296 | 46 | 650 | 652 | 74 | 57 | 1187 |
| 20 | 269 | 270 | 38 | 531 | 532 | 46 | 651 | 652 | 74 | 327 | 1187 |
| 20 | 270 | 271 | 38 | 531 | 533 | 46 | 651 | 653 | 74 | 450 | 1187 |
| 20 | 270 | 272 | 38 | 531 | 559 | 46 | 652 | 653 | 74 | 488 | 1187 |
| 20 | 270 | 276 | 38 | 531 | 563 | 47 | 3 | 656 | 74 | 526 | 1187 |
| 20 | 270 | 1217 | 38 | 531 | 570 | 47 | 3 | 657 | 74 | 1185 | 1187 |
| 20 | 271 | 272 | 38 | 531 | 576 | 47 | 3 | 658 | 74 | 1186 | 1187 |
| 20 | 271 | 273 | 38 | 531 | 591 | 47 | 3 | 659 | 74 | 1187 | 1188 |
| 20 | 271 | 274 | 38 | 531 | 593 | 47 | 3 | 660 | 74 | 1187 | 1189 |
| 20 | 271 | 275 | 38 | 531 | 1201 | 47 | 39 | 656 | 74 | 1187 | 1190 |
| 20 | 271 | 276 | 38 | 531 | 1427 | 47 | 48 | 656 | 74 | 1187 | 1191 |
| 20 | 272 | 273 | 38 | 532 | 533 | 47 | 48 | 660 | 74 | 1187 | 1192 |
| 20 | 272 | 274 | 38 | 532 | 559 | 47 | 61 | 660 | 74 | 1187 | 1293 |
| 20 | 272 | 275 | 38 | 532 | 591 | 47 | 63 | 656 | 75 | 1284 | 1285 |
| 20 | 272 | 276 | 38 | 532 | 593 | 47 | 63 | 657 | 75 | 1285 | 1286 |
| 20 | 272 | 1217 | 38 | 532 | 1200 | 47 | 63 | 659 | 76 | 64 | 1305 |
| 20 | 273 | 274 | 38 | 532 | 1201 | 47 | 66 | 656 | 76 | 135 | 1305 |
| 20 | 273 | 275 | 38 | 533 | 570 | 47 | 66 | 657 | 76 | 376 | 1305 |
| 20 | 273 | 276 | 38 | 533 | 591 | 47 | 105 | 659 | 76 | 1301 | 1302 |
| 20 | 273 | 839 | 38 | 533 | 593 | 47 | 123 | 660 | 76 | 1302 | 1303 |
| 20 | 273 | 1500 | 38 | 533 | 952 | 47 | 321 | 660 | 76 | 1302 | 1304 |
| 20 | 274 | 275 | 38 | 533 | 997 | 47 | 329 | 660 | 76 | 1303 | 1304 |
| 20 | 274 | 276 | 38 | 533 | 1010 | 47 | 381 | 656 | 76 | 1304 | 1305 |
| 20 | 274 | 838 | 38 | 533 | 1014 | 47 | 414 | 658 | 76 | 1305 | 1306 |
| 20 | 274 | 839 | 38 | 533 | 1047 | 47 | 443 | 659 | 77 | 1352 | 1353 |
| 20 | 274 | 1217 | 38 | 533 | 1118 | 47 | 445 | 660 | 77 | 1353 | 1354 |
| 20 | 275 | 276 | 38 | 533 | 1167 | 47 | 486 | 660 | 77 | 1353 | 1355 |
| 20 | 275 | 1217 | 38 | 533 | 1168 | 47 | 499 | 660 | 77 | 1354 | 1355 |
| 20 | 276 | 838 | 38 | 533 | 1200 | 47 | 500 | 658 | 78 | 926 | 1421 |
| 20 | 276 | 853 | 38 | 533 | 1201 | 47 | 505 | 659 | 78 | 1419 | 1421 |
| 20 | 276 | 863 | 38 | 533 | 1342 | 47 | 654 | 656 | 78 | 1420 | 1421 |
| 20 | 276 | 1217 | 38 | 533 | 1350 | 47 | 654 | 657 | 78 | 1421 | 1422 |
| 21 | 277 | 278 | 39 | 534 | 535 | 47 | 655 | 656 | 79 | 1458 | 1459 |
| 21 | 277 | 279 | 39 | 534 | 536 | 47 | 655 | 657 | 79 | 1459 | 1460 |
| 21 | 277 | 1214 | 39 | 534 | 537 | 47 | 656 | 657 | 79 | 1459 | 1461 |
| 21 | 278 | 279 | 39 | 534 | 555 | 47 | 656 | 658 | 79 | 1460 | 1461 |
| 21 | 278 | 280 | 39 | 534 | 584 | 47 | 656 | 659 | 80 | 101 | 1464 |
| 21 | 278 | 1214 | 39 | 534 | 590 | 47 | 657 | 658 | 80 | 1463 | 1464 |
| 21 | 280 | 283 | 39 | 534 | 592 | 47 | 657 | 659 | 80 | 1464 | 1465 |
| 22 | 289 | 293 | 39 | 534 | 1064 | 47 | 657 | 664 | 80 | 1464 | 1466 |
| 22 | 290 | 291 | 39 | 534 | 1147 | 47 | 658 | 659 | 80 | 1465 | 1466 |
| 22 | 291 | 293 | 39 | 534 | 1170 | 47 | 658 | 660 | 80 | 1466 | 1467 |
| 22 | 293 | 294 | 39 | 534 | 1202 | 47 | 659 | 660 | 80 | 1466 | 1468 |
