# Supplementary material for: Identification of novel genes associated with longevity in Drosophila melanogaster - a computational approach
Source: Aging (Albany NY). 2019 Dec 3;11(23):11244–67. doi: 10.18632/aging.102527 (PMC6932890; doi:10.18632/aging.102527)
Supplement: Supplementary Table 4 [file aging-11-102527-s002..docx]

**Supplementary Table 4. Nodes and interactions comprising each cluster of the extended DGRP GWAS-based networks.**

| Cluster | Bin 1 | Bin 2 | Cluster | Bin 1 | Bin 2 | Cluster | Bin 1 | Bin 2 | Cluster | Bin 1 | Bin 2 |
| --- | --- | --- | --- | --- | --- | --- | --- | --- | --- | --- | --- |
| 0 | 15 | 21 | 14 | 311 | 312 | 30 | 729 | 732 | 46 | 1116 | 1120 |
| 0 | 15 | 22 | 14 | 311 | 313 | 30 | 729 | 733 | 46 | 1117 | 1120 |
| 0 | 18 | 21 | 14 | 311 | 741 | 31 | 2 | 736 | 46 | 1118 | 1120 |
| 0 | 19 | 21 | 14 | 311 | 754 | 31 | 233 | 736 | 46 | 1119 | 1120 |
| 0 | 20 | 21 | 14 | 311 | 827 | 31 | 243 | 736 | 46 | 1120 | 1121 |
| 0 | 21 | 22 | 14 | 311 | 838 | 31 | 266 | 736 | 46 | 1120 | 1122 |
| 0 | 21 | 591 | 14 | 311 | 868 | 31 | 418 | 736 | 47 | 249 | 1124 |
| 0 | 21 | 592 | 14 | 311 | 872 | 31 | 734 | 736 | 47 | 1123 | 1124 |
| 0 | 21 | 600 | 14 | 311 | 886 | 31 | 735 | 736 | 47 | 1124 | 1125 |
| 0 | 21 | 643 | 14 | 311 | 907 | 31 | 736 | 737 | 47 | 1124 | 1126 |
| 0 | 21 | 1070 | 14 | 311 | 935 | 31 | 736 | 738 | 48 | 64 | 1131 |
| 0 | 21 | 1077 | 14 | 311 | 941 | 31 | 736 | 739 | 48 | 489 | 1131 |
| 0 | 21 | 1305 | 14 | 311 | 1209 | 31 | 736 | 740 | 48 | 1127 | 1131 |
| 0 | 21 | 1357 | 14 | 311 | 1211 | 31 | 736 | 1215 | 48 | 1127 | 1132 |
| 0 | 21 | 1444 | 14 | 311 | 1212 | 32 | 107 | 747 | 48 | 1128 | 1131 |
| 0 | 22 | 23 | 14 | 311 | 1213 | 32 | 745 | 747 | 48 | 1129 | 1131 |
| 0 | 22 | 24 | 14 | 311 | 1214 | 32 | 746 | 747 | 48 | 1129 | 1132 |
| 0 | 22 | 558 | 14 | 311 | 1216 | 32 | 747 | 748 | 48 | 1130 | 1131 |
| 0 | 22 | 562 | 14 | 311 | 1220 | 32 | 747 | 749 | 48 | 1130 | 1132 |
| 0 | 22 | 591 | 14 | 311 | 1221 | 32 | 747 | 750 | 48 | 1131 | 1132 |
| 0 | 22 | 646 | 14 | 311 | 1248 | 33 | 772 | 774 | 48 | 1131 | 1133 |
| 0 | 22 | 1067 | 14 | 311 | 1434 | 33 | 773 | 774 | 48 | 1131 | 1134 |
| 0 | 22 | 1110 | 14 | 311 | 1497 | 33 | 774 | 775 | 48 | 1132 | 1133 |
| 0 | 22 | 1114 | 15 | 338 | 340 | 33 | 774 | 1358 | 48 | 1132 | 1134 |
| 0 | 22 | 1411 | 15 | 339 | 340 | 34 | 318 | 778 | 48 | 1132 | 1135 |
| 0 | 23 | 24 | 15 | 340 | 341 | 34 | 776 | 778 | 49 | 63 | 1152 |
| 1 | 25 | 27 | 15 | 340 | 342 | 34 | 777 | 778 | 49 | 457 | 1152 |
| 1 | 26 | 27 | 15 | 340 | 343 | 34 | 778 | 779 | 49 | 1147 | 1152 |
| 1 | 27 | 28 | 16 | 481 | 484 | 34 | 778 | 780 | 49 | 1148 | 1152 |
| 1 | 27 | 30 | 16 | 482 | 484 | 34 | 778 | 781 | 49 | 1149 | 1152 |
| 1 | 27 | 1055 | 16 | 483 | 484 | 34 | 778 | 782 | 49 | 1150 | 1152 |
| 1 | 27 | 1115 | 16 | 484 | 485 | 34 | 778 | 783 | 49 | 1151 | 1152 |
| 1 | 27 | 1196 | 16 | 484 | 486 | 34 | 778 | 784 | 49 | 1152 | 1153 |
| 1 | 28 | 29 | 16 | 484 | 487 | 34 | 778 | 785 | 49 | 1152 | 1154 |
| 1 | 28 | 30 | 16 | 484 | 488 | 35 | 139 | 788 | 49 | 1152 | 1155 |
| 1 | 29 | 30 | 17 | 507 | 509 | 35 | 218 | 787 | 50 | 1156 | 1157 |
| 1 | 29 | 31 | 17 | 508 | 509 | 35 | 337 | 787 | 50 | 1157 | 1158 |
| 1 | 29 | 1192 | 17 | 509 | 510 | 35 | 349 | 787 | 50 | 1157 | 1159 |
| 1 | 29 | 1195 | 17 | 509 | 511 | 35 | 358 | 787 | 50 | 1157 | 1160 |
| 1 | 30 | 31 | 17 | 509 | 585 | 35 | 387 | 787 | 50 | 1157 | 1161 |
| 1 | 30 | 1036 | 17 | 509 | 942 | 35 | 387 | 788 | 50 | 1157 | 1162 |
| 1 | 30 | 1191 | 18 | 523 | 529 | 35 | 786 | 787 | 50 | 1157 | 1163 |
| 2 | 12 | 34 | 18 | 524 | 529 | 35 | 787 | 788 | 51 | 14 | 1168 |
| 2 | 32 | 34 | 18 | 525 | 529 | 35 | 787 | 789 | 51 | 17 | 1168 |
| 2 | 33 | 34 | 18 | 526 | 529 | 35 | 787 | 790 | 51 | 1165 | 1168 |
| 2 | 34 | 35 | 18 | 527 | 529 | 35 | 787 | 1432 | 51 | 1166 | 1168 |
| 2 | 34 | 36 | 18 | 528 | 529 | 35 | 788 | 789 | 51 | 1167 | 1168 |
| 3 | 38 | 44 | 18 | 529 | 530 | 35 | 788 | 790 | 51 | 1168 | 1169 |
| 3 | 40 | 44 | 18 | 529 | 531 | 35 | 788 | 792 | 51 | 1168 | 1170 |
| 3 | 42 | 44 | 18 | 529 | 560 | 36 | 878 | 880 | 52 | 1171 | 1173 |
| 3 | 43 | 44 | 18 | 529 | 1253 | 36 | 879 | 880 | 52 | 1172 | 1173 |
| 3 | 44 | 45 | 19 | 533 | 534 | 36 | 880 | 881 | 52 | 1173 | 1174 |
| 3 | 44 | 46 | 19 | 534 | 535 | 36 | 880 | 882 | 52 | 1173 | 1326 |
| 3 | 44 | 47 | 19 | 534 | 536 | 36 | 880 | 883 | 53 | 3 | 1178 |
| 3 | 44 | 48 | 19 | 534 | 537 | 36 | 880 | 884 | 53 | 3 | 1179 |
| 3 | 44 | 556 | 19 | 534 | 554 | 36 | 880 | 885 | 53 | 6 | 1178 |
| 3 | 44 | 564 | 19 | 534 | 555 | 37 | 887 | 890 | 53 | 6 | 1179 |
| 3 | 44 | 567 | 19 | 534 | 559 | 37 | 888 | 890 | 53 | 7 | 1178 |
| 3 | 44 | 569 | 19 | 534 | 561 | 37 | 889 | 890 | 53 | 7 | 1179 |
| 3 | 44 | 583 | 19 | 534 | 568 | 37 | 890 | 891 | 53 | 359 | 1179 |
| 3 | 44 | 584 | 19 | 534 | 570 | 37 | 890 | 892 | 53 | 414 | 1179 |
| 3 | 44 | 594 | 19 | 534 | 571 | 37 | 890 | 893 | 53 | 422 | 1179 |
| 3 | 44 | 602 | 19 | 534 | 590 | 37 | 890 | 894 | 53 | 454 | 1179 |
| 3 | 44 | 605 | 19 | 534 | 606 | 37 | 890 | 895 | 53 | 476 | 1179 |
| 3 | 44 | 927 | 19 | 534 | 711 | 37 | 890 | 1217 | 53 | 493 | 1178 |
| 3 | 44 | 982 | 19 | 534 | 756 | 37 | 890 | 1222 | 53 | 493 | 1179 |
| 3 | 44 | 1072 | 19 | 534 | 926 | 38 | 952 | 956 | 53 | 506 | 1179 |
| 3 | 44 | 1206 | 19 | 534 | 1069 | 38 | 953 | 956 | 53 | 518 | 1178 |
| 3 | 44 | 1300 | 19 | 534 | 1082 | 38 | 954 | 956 | 53 | 518 | 1179 |
| 3 | 44 | 1416 | 19 | 534 | 1105 | 38 | 955 | 956 | 53 | 532 | 1178 |
| 3 | 45 | 46 | 19 | 534 | 1106 | 38 | 956 | 957 | 53 | 538 | 1179 |
| 3 | 45 | 47 | 19 | 534 | 1108 | 38 | 956 | 958 | 53 | 542 | 1178 |
| 3 | 46 | 47 | 19 | 534 | 1109 | 38 | 956 | 959 | 53 | 542 | 1179 |
| 3 | 46 | 48 | 19 | 534 | 1164 | 38 | 956 | 960 | 53 | 545 | 1178 |
| 3 | 46 | 49 | 19 | 534 | 1246 | 38 | 956 | 961 | 53 | 545 | 1179 |
| 3 | 46 | 604 | 19 | 534 | 1250 | 38 | 957 | 958 | 53 | 546 | 1178 |
| 3 | 46 | 707 | 19 | 534 | 1255 | 38 | 958 | 959 | 53 | 546 | 1179 |
| 3 | 46 | 1033 | 19 | 534 | 1307 | 38 | 958 | 960 | 53 | 547 | 1178 |
| 3 | 46 | 1143 | 20 | 105 | 576 | 38 | 958 | 961 | 53 | 547 | 1179 |
| 3 | 46 | 1462 | 20 | 478 | 576 | 39 | 965 | 970 | 53 | 1175 | 1178 |
| 3 | 47 | 48 | 20 | 521 | 576 | 39 | 966 | 970 | 53 | 1175 | 1179 |
| 3 | 47 | 49 | 20 | 572 | 576 | 39 | 967 | 970 | 53 | 1176 | 1178 |
| 4 | 53 | 56 | 20 | 573 | 576 | 39 | 968 | 970 | 53 | 1177 | 1178 |
| 4 | 54 | 56 | 20 | 574 | 576 | 39 | 969 | 970 | 53 | 1177 | 1179 |
| 4 | 55 | 56 | 20 | 575 | 576 | 39 | 970 | 971 | 53 | 1178 | 1179 |
| 4 | 56 | 57 | 20 | 576 | 577 | 39 | 970 | 972 | 53 | 1178 | 1180 |
| 4 | 56 | 58 | 20 | 576 | 578 | 39 | 970 | 973 | 53 | 1178 | 1181 |
| 4 | 56 | 59 | 20 | 576 | 579 | 39 | 970 | 974 | 53 | 1178 | 1279 |
| 4 | 56 | 60 | 20 | 576 | 580 | 40 | 103 | 989 | 53 | 1179 | 1180 |
| 4 | 56 | 61 | 20 | 576 | 581 | 40 | 426 | 989 | 53 | 1179 | 1181 |
| 4 | 56 | 1078 | 20 | 576 | 1227 | 40 | 458 | 989 | 53 | 1179 | 1247 |
| 4 | 56 | 1079 | 21 | 66 | 609 | 40 | 983 | 989 | 53 | 1179 | 1420 |
| 4 | 56 | 1338 | 21 | 541 | 609 | 40 | 985 | 989 | 54 | 1 | 1183 |
| 5 | 87 | 89 | 21 | 607 | 609 | 40 | 986 | 989 | 54 | 10 | 1183 |
| 5 | 88 | 89 | 21 | 608 | 609 | 40 | 987 | 989 | 54 | 37 | 1183 |
| 5 | 88 | 91 | 21 | 609 | 610 | 40 | 988 | 989 | 54 | 65 | 1183 |
| 5 | 89 | 90 | 21 | 609 | 611 | 40 | 989 | 990 | 54 | 85 | 1183 |
| 5 | 89 | 91 | 21 | 609 | 612 | 40 | 989 | 991 | 54 | 98 | 1183 |
| 5 | 89 | 93 | 22 | 613 | 618 | 40 | 989 | 992 | 54 | 104 | 1183 |
| 5 | 89 | 603 | 22 | 613 | 619 | 40 | 989 | 1210 | 54 | 166 | 1183 |
| 5 | 89 | 1012 | 22 | 614 | 618 | 40 | 989 | 1336 | 54 | 336 | 1183 |
| 5 | 90 | 91 | 22 | 614 | 619 | 41 | 999 | 1003 | 54 | 377 | 1183 |
| 5 | 91 | 92 | 22 | 616 | 618 | 41 | 999 | 1005 | 54 | 390 | 1183 |
| 5 | 91 | 93 | 22 | 616 | 619 | 41 | 1000 | 1003 | 54 | 431 | 1183 |
| 5 | 91 | 94 | 22 | 617 | 618 | 41 | 1000 | 1005 | 54 | 437 | 1183 |
| 5 | 91 | 582 | 22 | 617 | 619 | 41 | 1001 | 1003 | 54 | 440 | 1183 |
| 5 | 91 | 1086 | 22 | 618 | 619 | 41 | 1001 | 1005 | 54 | 446 | 1183 |
| 6 | 108 | 110 | 22 | 618 | 620 | 41 | 1002 | 1003 | 54 | 453 | 1183 |
| 6 | 109 | 110 | 22 | 619 | 620 | 41 | 1002 | 1005 | 54 | 462 | 1183 |
| 6 | 110 | 111 | 23 | 621 | 622 | 41 | 1003 | 1004 | 54 | 463 | 1183 |
| 6 | 110 | 112 | 23 | 622 | 623 | 41 | 1003 | 1005 | 54 | 466 | 1183 |
| 6 | 110 | 1193 | 23 | 622 | 624 | 41 | 1004 | 1005 | 54 | 470 | 1183 |
| 7 | 113 | 114 | 23 | 622 | 625 | 41 | 1005 | 1006 | 54 | 477 | 1183 |
| 7 | 114 | 115 | 24 | 369 | 631 | 41 | 1005 | 1007 | 54 | 496 | 1183 |
| 7 | 114 | 116 | 24 | 628 | 631 | 41 | 1005 | 1008 | 54 | 501 | 1183 |
| 7 | 114 | 117 | 24 | 629 | 631 | 42 | 261 | 1045 | 54 | 512 | 1183 |
| 7 | 114 | 118 | 24 | 630 | 631 | 42 | 502 | 1045 | 54 | 519 | 1183 |
| 7 | 114 | 119 | 24 | 631 | 632 | 42 | 1043 | 1045 | 54 | 522 | 1183 |
| 8 | 120 | 123 | 24 | 631 | 633 | 42 | 1044 | 1045 | 54 | 539 | 1183 |
| 8 | 121 | 123 | 24 | 631 | 634 | 42 | 1045 | 1046 | 54 | 543 | 1183 |
| 8 | 122 | 123 | 25 | 244 | 655 | 42 | 1045 | 1047 | 54 | 544 | 1183 |
| 8 | 123 | 124 | 25 | 653 | 655 | 42 | 1045 | 1048 | 54 | 548 | 1183 |
| 8 | 123 | 125 | 25 | 654 | 655 | 42 | 1045 | 1049 | 54 | 1182 | 1183 |
| 8 | 123 | 126 | 25 | 655 | 656 | 42 | 1046 | 1050 | 54 | 1183 | 1184 |
| 8 | 124 | 126 | 25 | 655 | 657 | 42 | 1047 | 1050 | 54 | 1183 | 1185 |
| 8 | 125 | 126 | 26 | 321 | 660 | 42 | 1048 | 1050 | 54 | 1183 | 1186 |
| 8 | 126 | 127 | 26 | 329 | 660 | 42 | 1049 | 1050 | 54 | 1183 | 1187 |
| 8 | 126 | 1104 | 26 | 412 | 660 | 42 | 1050 | 1051 | 54 | 1183 | 1188 |
| 9 | 128 | 129 | 26 | 445 | 660 | 42 | 1050 | 1052 | 54 | 1183 | 1256 |
| 9 | 129 | 130 | 26 | 499 | 660 | 42 | 1050 | 1054 | 54 | 1183 | 1328 |
| 9 | 129 | 131 | 26 | 658 | 660 | 43 | 4 | 1063 | 54 | 1183 | 1329 |
| 9 | 129 | 764 | 26 | 659 | 660 | 43 | 515 | 1063 | 54 | 1183 | 1366 |
| 9 | 129 | 928 | 26 | 660 | 661 | 43 | 1056 | 1058 | 55 | 9 | 1199 |
| 9 | 129 | 930 | 26 | 660 | 662 | 43 | 1057 | 1058 | 55 | 11 | 1199 |
| 9 | 129 | 997 | 26 | 660 | 663 | 43 | 1057 | 1063 | 55 | 76 | 1199 |
| 9 | 129 | 1107 | 26 | 660 | 1324 | 43 | 1058 | 1059 | 55 | 409 | 1199 |
| 9 | 129 | 1421 | 26 | 660 | 1367 | 43 | 1058 | 1060 | 55 | 455 | 1199 |
| 10 | 132 | 133 | 26 | 660 | 1418 | 43 | 1058 | 1061 | 55 | 498 | 1199 |
| 10 | 133 | 134 | 27 | 635 | 668 | 43 | 1058 | 1062 | 55 | 504 | 1199 |
| 10 | 133 | 135 | 27 | 664 | 668 | 43 | 1059 | 1063 | 55 | 513 | 1199 |
| 10 | 133 | 682 | 27 | 665 | 668 | 43 | 1060 | 1063 | 55 | 517 | 1199 |
| 11 | 156 | 159 | 27 | 666 | 668 | 43 | 1061 | 1063 | 55 | 540 | 1199 |
| 11 | 157 | 159 | 27 | 667 | 668 | 43 | 1062 | 1063 | 55 | 549 | 1199 |
| 11 | 158 | 159 | 28 | 330 | 670 | 43 | 1063 | 1064 | 55 | 1197 | 1199 |
| 11 | 159 | 160 | 28 | 344 | 670 | 43 | 1063 | 1065 | 55 | 1198 | 1199 |
| 11 | 159 | 161 | 28 | 366 | 670 | 43 | 1063 | 1066 | 55 | 1199 | 1200 |
| 11 | 159 | 162 | 28 | 413 | 670 | 43 | 1063 | 1249 | 55 | 1199 | 1201 |
| 11 | 159 | 163 | 28 | 669 | 670 | 44 | 361 | 1091 | 55 | 1199 | 1202 |
| 11 | 159 | 1026 | 28 | 670 | 671 | 44 | 429 | 1091 | 55 | 1199 | 1203 |
| 12 | 187 | 190 | 28 | 670 | 672 | 44 | 448 | 1091 | 55 | 1199 | 1254 |
| 12 | 188 | 190 | 28 | 670 | 673 | 44 | 1089 | 1091 | 56 | 550 | 1231 |
| 12 | 188 | 191 | 28 | 670 | 1419 | 44 | 1090 | 1091 | 56 | 553 | 1231 |
| 12 | 189 | 190 | 29 | 80 | 698 | 44 | 1091 | 1092 | 56 | 557 | 1231 |
| 12 | 189 | 191 | 29 | 199 | 699 | 44 | 1091 | 1093 | 56 | 593 | 1231 |
| 12 | 190 | 191 | 29 | 696 | 698 | 44 | 1091 | 1094 | 56 | 1010 | 1231 |
| 12 | 190 | 192 | 29 | 697 | 698 | 44 | 1091 | 1095 | 56 | 1204 | 1231 |
| 12 | 190 | 193 | 29 | 697 | 699 | 45 | 468 | 1097 | 56 | 1205 | 1231 |
| 12 | 190 | 194 | 29 | 698 | 699 | 45 | 1096 | 1097 | 56 | 1230 | 1231 |
| 12 | 190 | 195 | 29 | 698 | 700 | 45 | 1097 | 1098 | 56 | 1231 | 1232 |
| 12 | 191 | 192 | 29 | 698 | 701 | 45 | 1097 | 1099 | 57 | 364 | 1261 |
| 12 | 191 | 193 | 29 | 698 | 702 | 45 | 1097 | 1100 | 57 | 1259 | 1261 |
| 12 | 191 | 194 | 29 | 698 | 703 | 45 | 1097 | 1103 | 57 | 1260 | 1261 |
| 12 | 191 | 195 | 29 | 699 | 700 | 45 | 1097 | 1294 | 57 | 1261 | 1262 |
| 13 | 206 | 208 | 29 | 699 | 701 | 46 | 5 | 1120 | 58 | 1271 | 1272 |
| 13 | 207 | 208 | 29 | 699 | 702 | 46 | 39 | 1120 | 58 | 1272 | 1273 |
| 13 | 208 | 209 | 29 | 699 | 703 | 46 | 84 | 1120 | 58 | 1272 | 1274 |
| 14 | 307 | 311 | 30 | 727 | 729 | 46 | 267 | 1120 | 59 | 1339 | 1340 |
| 14 | 308 | 311 | 30 | 728 | 729 | 46 | 408 | 1120 | 59 | 1340 | 1341 |
| 14 | 309 | 311 | 30 | 729 | 730 | 46 | 425 | 1120 | 60 | 1453 | 1454 |
| 14 | 310 | 311 | 30 | 729 | 731 | 46 | 514 | 1120 | 60 | 1454 | 1455 |
