# Supplementary material for: Identification of novel genes associated with longevity in Drosophila melanogaster - a computational approach
Source: Aging (Albany NY). 2019 Dec 3;11(23):11244–67. doi: 10.18632/aging.102527 (PMC6932890; doi:10.18632/aging.102527)
Supplement: Supplementary Table 7 [file aging-11-102527-s003..docx]

**Supplementary Table 7. Genes sharing ‘long-lived’ and ‘short-lived’ phenotypes found in close proximity to mutated and non-mutated TAD border regions.**

| **Gene** | **SNPs in TAD**  **border region** | **TAD border position** | **TAD border region** |
| --- | --- | --- | --- |
| ap | YES | 2R:1589494 | 2R:1589394-1589594 |
| Atg7 | YES | 2R:14519600 | 2R:14519500-14519700 |
| Atpalpha | YES | 3R:16774364 | 3R:16774264-16774464 |
| ATPsynbetaL | YES | 3L:16083568 | 3L:16083468-16083668 |
| azot | YES | 2R:3843665 | 2R:3843565-3843765 |
| bmm | YES | 3L:14769200 | 3L:14769100-14769300 |
| Charon | YES | 2L:1164178 | 2L:1164078-1164278 |
| Charon | YES | 2L:1185674 | 2L:1185574-1185774 |
| Coq2 | YES | 3R:4131593 | 3R:4131493-4131693 |
| Debcl | YES | 2R:2532812 | 2R:2532712-2532912 |
| Debcl | YES | 2R:2549803 | 2R:2549703-2549903 |
| Diedel | YES | 3R:25329565 | 3R:25329465-25329665 |
| EcR | YES | 2R:1973416 | 2R:1973316-1973516 |
| EcR | YES | 2R:1964288 | 2R:1964188-1964388 |
| fh | YES | X:9036041 | X:9035941-9036141 |
| foxo | YES | 3R:9915094 | 3R:9914994-9915194 |
| Glo1 | YES | 2R:3295533 | 2R:3295433-3295633 |
| GlyP | YES | 2L:2130843 | 2L:2130743-2130943 |
| GlyS | YES | 3R:10973968 | 3R:10973868-10974068 |
| Ide | YES | 3L:20369097 | 3L:20368997-20369197 |
| Ide | YES | 3L:20354785 | 3L:20354685-20354885 |
| ImpL2 | YES | 3L:4239967 | 3L:4239867-4240067 |
| inaE | YES | X:13676418 | X:13676318-13676518 |
| inaE | YES | X:13716489 | X:13716389-13716589 |
| IncRNA:let7C | YES | 2L:18485351 | 2L:18485251-18485451 |
| InR | YES | 3R:17445387 | 3R:17445287-17445487 |
| Jafrac1 | YES | X:13227959 | X:13227859-13228059 |
| jp | YES | 2L:9567970 | 2L:9567870-9568070 |
| jp | YES | 2L:9576982 | 2L:9576882-9577082 |
| mei-9 | YES | X:4216268 | X:4216168-4216368 |
| Mrp4 | YES | 3R:7415809 | 3R:7415709-7415909 |
| MTF-1 | YES | 3L:9435499 | 3L:9435399-9435599 |
| Nadsyn | YES | X:13562020 | X:13561920-13562120 |
| p120ctn | YES | 2R:498879 | 2R:498779-498979 |
| Pcl | YES | 2R:14013277 | 2R:14013177-14013377 |
| Pmm2 | YES | 3L:12520015 | 3L:12519915-12520115 |
| Pten | YES | 2L:10265301 | 2L:10265201-10265401 |
| S6k | YES | 3L:5787264 | 3L:5787164-5787364 |
| slpr | YES | X:8072499 | X:8072399-8072599 |
| sm | YES | 2R:15390386 | 2R:15390286-15390486 |
| sNPF | YES | 2L:20026577 | 2L:20026477-20026677 |
| sra | YES | 3R:12030956 | 3R:12030856-12031056 |
| stai | YES | 2L:6098914 | 2L:6098814-6099014 |
| sun | YES | X:15743514 | X:15743414-15743614 |
| tefu | YES | 3R:11051226 | 3R:11051126-11051326 |
| teq | YES | 3L:9065799 | 3L:9065699-9065899 |
| teq | YES | 3L:9088271 | 3L:9088171-9088371 |
| Thor | YES | 2L:3480545 | 2L:3480445-3480645 |
| Trx-2 | YES | 2L:9613036 | 2L:9612936-9613136 |
| upd3 | YES | X:18168713 | X:18168613-18168813 |
| Zip89B | YES | 3R:12136164 | 3R:12136064-12136264 |
| Akh | NO | 3L:4139254 | 3L:4139154-4139354 |
| Akt1 | NO | 3R:11925932 | 3R:11925832-11926032 |
| AMPKalpha | NO | X:1269714 | X:1269614-1269814 |
| AMPKalpha | NO | X:1288192 | X:1288092-1288292 |
| aop | NO | 2L:2160995 | 2L:2160895-2161095 |
| aop/Glyp | NO | 2L:2151583 | 2L:2151483-2151683 |
| ap | NO | 2R:1585588 | 2R:1585488-1585688 |
| Atg7 | NO | 2R:14517627 | 2R:14517527-14517727 |
| Atg7 | NO | 2R:14499646 | 2R:14499546-14499746 |
| Atg7 | NO | 2R:14526182 | 2R:14526082-14526282 |
| Atg8a | NO | X:10659694 | X:10659594-10659794 |
| Atpalpha | NO | 3R:16804469 | 3R:16804369-16804569 |
| ATPsynbetaL | NO | 3L:16092935 | 3L:16092835-16093035 |
| ATPsynbetaL | NO | 3L:16106558 | 3L:16106458-16106658 |
| azot | NO | 2R:3827859 | 2R:3827759-3827959 |
| BI-1 | NO | 3L:8308401 | 3L:8308301-8308501 |
| BI-1 | NO | 3L:8292805 | 3L:8292705-8292905 |
| bmm | NO | 3L:14752415 | 3L:14752315-14752515 |
| bwa | NO | 2L:20060644 | 2L:20060544-20060744 |
| Cbs | NO | X:20931096 | X:20930996-20931196 |
| cher | NO | 3R:12906372 | 3R:12906272-12906472 |
| cher | NO | 3R:12945216 | 3R:12945116-12945316 |
| Cln3 | NO | 3L:17795259 | 3L:17795159-17795359 |
| Cyp4e3 | NO | 2L:9734314 | 2L:9734214-9734414 |
| Cyp4e3 | NO | 2L:9759187 | 2L:9759087-9759287 |
| Debcl | NO | 2R:2519984 | 2R:2519884-2520084 |
| Diedel | NO | 3R:25316993 | 3R:25316893-25317093 |
| Diedel | NO | 3R:25305202 | 3R:25305102-25305302 |
| dj-1beta | NO | 3R:26634960 | 3R:26634860-26635060 |
| dj-1beta | NO | 3R:26620592 | 3R:26620492-26620692 |
| Dl | NO | 3R:15135409 | 3R:15135309-15135509 |
| dnc | NO | X:3067862 | X:3067762-3067962 |
| dnr1 | NO | 2R:18450174 | 2R:18450074-18450274 |
| dnr1 | NO | 2R:18485082 | 2R:18484982-18485182 |
| dnr1 | NO | 2R:18493615 | 2R:18493515-18493715 |
| E(z) | NO | 3L:10623942 | 3L:10623842-10624042 |
| EcR | NO | 2R:1994410 | 2R:1994310-1994510 |
| EcR | NO | 2R:2038815 | 2R:2038715-2038915 |
| Egm | NO | 2R:7506293 | 2R:7506193-7506393 |
| esc | NO | 2L:11829818 | 2L:11829718-11829918 |
| esc | NO | 2L:11813649 | 2L:11813549-11813749 |
| fh | NO | X:9057982 | X:9057882-9058082 |
| Glaz | NO | 2R:9056097 | 2R:9055997-9056197 |
| glob1 | NO | 3R:11736839 | 3R:11736739-11736939 |
| GlyS | NO | 3R:10959843 | 3R:10959743-10959943 |
| HDAC1 | NO | 3L:4626677 | 3L:4626577-4626777 |
| hep | NO | X:12979165 | X:12979065-12979265 |
| Hsp22/Hsp27 | NO | 3L:9372279 | 3L:9372179-9372379 |
| Hsp22/Hsp27 | NO | 3L:9351645 | 3L:9351545-9351745 |
| htt | NO | 3R:24521032 | 3R:24520932-24521132 |
| ImpL2 | NO | 3L:4219625 | 3L:4219525-4219725 |
| IncRNA:let7C | NO | 2L:18454874 | 2L:18454774-18454974 |
| Indy | NO | 3L:18813002 | 3L:18812902-18813102 |
| InR | NO | 3R:17400261 | 3R:17400161-17400361 |
| iPLA2-VIA | NO | 3L:9853496 | 3L:9853396-9853596 |
| jp | NO | 2L:9540667 | 2L:9540567-9540767 |
| Lnk | NO | 3R:21714600 | 3R:21714500-21714700 |
| miple1/miple2 | NO | 3L:265050 | 3L:264950-265150 |
| mir-34 | NO | 3R:5934567 | 3R:5934467-5934667 |
| mir-8 | NO | 2R:12717713 | 2R:12717613-12717813 |
| mir-965 | NO | 2L:251331 | 2L:251231-251431 |
| Mrp4 | NO | 3R:7407293 | 3R:7407193-7407393 |
| Mrp4 | NO | 3R:7388521 | 3R:7388421-7388621 |
| Mt2 | NO | 2L:12093653 | 2L:12093553-12093753 |
| Mt2 | NO | 2L:12108860 | 2L:12108760-12108960 |
| MTF-1 | NO | 3L:9422683 | 3L:9422583-9422783 |
| mub | NO | 3L:21833026 | 3L:21832926-21833126 |
| Myc | NO | X:3238412 | X:3238312-3238512 |
| Myc | NO | X:3287315 | X:3287215-3287415 |
| Nadsyn | NO | X:13574417 | X:13574317-13574517 |
| Nadsyn | NO | X:13613311 | X:13613211-13613411 |
| ND-23 | NO | 3R:11661022 | 3R:11660922-11661122 |
| ND-SGDH | NO | 3L:12139909 | 3L:12139809-12140009 |
| Nf1 | NO | 3R:21808836 | 3R:21808736-21808936 |
| Nf1 | NO | 3R:21830112 | 3R:21830012-21830212 |
| Nlaz | NO | 2L:1348458 | 2L:1348358-1348558 |
| Nmdmc | NO | 3R:4865483 | 3R:4865383-4865583 |
| Nmdmc | NO | 3R:4873967 | 3R:4873867-4874067 |
| Nsun5 | NO | 3R:14987901 | 3R:14987801-14988001 |
| p53 | NO | 3R:18875135 | 3R:18875035-18875235 |
| p53 | NO | 3R:18888382 | 3R:18888282-18888482 |
| p88b | NO | 2L:13780207 | 2L:13780107-13780307 |
| park | NO | 3L:21189706 | 3L:21189606-21189806 |
| park | NO | 3L:21195173 | 3L:21195073-21195273 |
| Pgam5 | NO | X:1767917 | X:1767817-1768017 |
| Pgam5 | NO | X:1753234 | X:1753134-1753334 |
| Pi3K92E/LRRK2 | NO | 3R:16470119 | 3R:16470019-16470219 |
| Pi3K92E/LRRK2 | NO | 3R:16461594 | 3R:16461494-16461694 |
| Pmm2 | NO | 3L:12530235 | 3L:12530135-12530335 |
| Prx5 | NO | 3R:13990022 | 3R:13989922-13990122 |
| Pten | NO | 2L:10270185 | 2L:10270085-10270285 |
| Ras85D | NO | 3R:5323137 | 3R:5323037-5323237 |
| Ras85D | NO | 3R:5341240 | 3R:5341140-5341340 |
| S6k | NO | 3L:5806066 | 3L:5805966-5806166 |
| Scox | NO | 2L:4955929 | 2L:4955829-4956029 |
| Scox | NO | 2L:4971507 | 2L:4971407-4971607 |
| Scsalpha1 | NO | 3L:3812961 | 3L:3812861-3813061 |
| SelR | NO | 3R:6695234 | 3R:6695134-6695334 |
| SelR | NO | 3R:6677524 | 3R:6677424-6677624 |
| sgg | NO | X:2527673 | X:2527573-2527773 |
| sgg | NO | X:2557924 | X:2557824-2558024 |
| Sirt1 | NO | 2L:13165210 | 2L:13165110-13165310 |
| Sirt4 | NO | X:5559776 | X:5559676-5559876 |
| Sirt4 | NO | X:5565787 | X:5565687-5565887 |
| Sod1 | NO | 3L:11116217 | 3L:11116117-11116317 |
| Sod2 | NO | 2R:12660576 | 2R:12660476-12660676 |
| sra | NO | 3R:12013037 | 3R:12012937-12013137 |
| sturkopf | NO | 3L:1318273 | 3L:1318173-1318373 |
| tefu | NO | 3R:11068610 | 3R:11068510-11068710 |
| Thor | NO | 2L:3470304 | 2L:3470204-3470404 |
| Tpi | NO | 3R:25960027 | 3R:25959927-25960127 |
| Tpi | NO | 3R:25979717 | 3R:25979617-25979817 |
| Trx-2 | NO | 2L:9617247 | 2L:9617147-9617347 |
| Ucp4A | NO | X:17736806 | X:17736706-17736906 |
| Ucp4A | NO | X:17754347 | X:17754247-17754447 |
| VhaSFD | NO | 2L:16721543 | 2L:16721443-16721643 |
| VhaSFD | NO | 2L:16727693 | 2L:16727593-16727793 |
| Xrp1 | NO | 3R:14755317 | 3R:14755217-14755417 |
| Xrp1 | NO | 3R:14732623 | 3R:14732523-14732723 |
| Zip89B | NO | 3R:12144946 | 3R:12144846-12145046 |
