# Supplementary material for: Identification of novel genes associated with longevity in Drosophila melanogaster - a computational approach
Source: Aging (Albany NY). 2019 Dec 3;11(23):11244–67. doi: 10.18632/aging.102527 (PMC6932890; doi:10.18632/aging.102527)
Supplement: Supplementary Table 10 [file aging-11-102527-s006..docx]

**Supplementary Table 10**. **Chromosome, start and end positions for each 80 Kb bin.**

| Bin | chr | Start position | End position | Bin | chr | Start position | End position |
| --- | --- | --- | --- | --- | --- | --- | --- |
| 1 | 2L | 0 | 80000 | 752 | 3L | 16000000 | 16080000 |
| 2 | 2L | 80000 | 160000 | 753 | 3L | 16080000 | 16160000 |
| 3 | 2L | 160000 | 240000 | 754 | 3L | 16160000 | 16240000 |
| 4 | 2L | 240000 | 320000 | 755 | 3L | 16240000 | 16320000 |
| 5 | 2L | 320000 | 400000 | 756 | 3L | 16320000 | 16400000 |
| 6 | 2L | 400000 | 480000 | 757 | 3L | 16400000 | 16480000 |
| 7 | 2L | 480000 | 560000 | 758 | 3L | 16480000 | 16560000 |
| 8 | 2L | 560000 | 640000 | 759 | 3L | 16560000 | 16640000 |
| 9 | 2L | 640000 | 720000 | 760 | 3L | 16640000 | 16720000 |
| 10 | 2L | 720000 | 800000 | 761 | 3L | 16720000 | 16800000 |
| 11 | 2L | 800000 | 880000 | 762 | 3L | 16800000 | 16880000 |
| 12 | 2L | 880000 | 960000 | 763 | 3L | 16880000 | 16960000 |
| 13 | 2L | 960000 | 1040000 | 764 | 3L | 16960000 | 17040000 |
| 14 | 2L | 1040000 | 1120000 | 765 | 3L | 17040000 | 17120000 |
| 15 | 2L | 1120000 | 1200000 | 766 | 3L | 17120000 | 17200000 |
| 16 | 2L | 1200000 | 1280000 | 767 | 3L | 17200000 | 17280000 |
| 17 | 2L | 1280000 | 1360000 | 768 | 3L | 17280000 | 17360000 |
| 18 | 2L | 1360000 | 1440000 | 769 | 3L | 17360000 | 17440000 |
| 19 | 2L | 1440000 | 1520000 | 770 | 3L | 17440000 | 17520000 |
| 20 | 2L | 1520000 | 1600000 | 771 | 3L | 17520000 | 17600000 |
| 21 | 2L | 1600000 | 1680000 | 772 | 3L | 17600000 | 17680000 |
| 22 | 2L | 1680000 | 1760000 | 773 | 3L | 17680000 | 17760000 |
| 23 | 2L | 1760000 | 1840000 | 774 | 3L | 17760000 | 17840000 |
| 24 | 2L | 1840000 | 1920000 | 775 | 3L | 17840000 | 17920000 |
| 25 | 2L | 1920000 | 2000000 | 776 | 3L | 17920000 | 18000000 |
| 26 | 2L | 2000000 | 2080000 | 777 | 3L | 18000000 | 18080000 |
| 27 | 2L | 2080000 | 2160000 | 778 | 3L | 18080000 | 18160000 |
| 28 | 2L | 2160000 | 2240000 | 779 | 3L | 18160000 | 18240000 |
| 29 | 2L | 2240000 | 2320000 | 780 | 3L | 18240000 | 18320000 |
| 30 | 2L | 2320000 | 2400000 | 781 | 3L | 18320000 | 18400000 |
| 31 | 2L | 2400000 | 2480000 | 782 | 3L | 18400000 | 18480000 |
| 32 | 2L | 2480000 | 2560000 | 783 | 3L | 18480000 | 18560000 |
| 33 | 2L | 2560000 | 2640000 | 784 | 3L | 18560000 | 18640000 |
| 34 | 2L | 2640000 | 2720000 | 785 | 3L | 18640000 | 18720000 |
| 35 | 2L | 2720000 | 2800000 | 786 | 3L | 18720000 | 18800000 |
| 36 | 2L | 2800000 | 2880000 | 787 | 3L | 18800000 | 18880000 |
| 37 | 2L | 2880000 | 2960000 | 788 | 3L | 18880000 | 18960000 |
| 38 | 2L | 2960000 | 3040000 | 789 | 3L | 18960000 | 19040000 |
| 39 | 2L | 3040000 | 3120000 | 790 | 3L | 19040000 | 19120000 |
| 40 | 2L | 3120000 | 3200000 | 791 | 3L | 19120000 | 19200000 |
| 41 | 2L | 3200000 | 3280000 | 792 | 3L | 19200000 | 19280000 |
| 42 | 2L | 3280000 | 3360000 | 793 | 3L | 19280000 | 19360000 |
| 43 | 2L | 3360000 | 3440000 | 794 | 3L | 19360000 | 19440000 |
| 44 | 2L | 3440000 | 3520000 | 795 | 3L | 19440000 | 19520000 |
| 45 | 2L | 3520000 | 3600000 | 796 | 3L | 19520000 | 19600000 |
| 46 | 2L | 3600000 | 3680000 | 797 | 3L | 19600000 | 19680000 |
| 47 | 2L | 3680000 | 3760000 | 798 | 3L | 19680000 | 19760000 |
| 48 | 2L | 3760000 | 3840000 | 799 | 3L | 19760000 | 19840000 |
| 49 | 2L | 3840000 | 3920000 | 800 | 3L | 19840000 | 19920000 |
| 50 | 2L | 3920000 | 4000000 | 801 | 3L | 19920000 | 20000000 |
| 51 | 2L | 4000000 | 4080000 | 802 | 3L | 20000000 | 20080000 |
| 52 | 2L | 4080000 | 4160000 | 803 | 3L | 20080000 | 20160000 |
| 53 | 2L | 4160000 | 4240000 | 804 | 3L | 20160000 | 20240000 |
| 54 | 2L | 4240000 | 4320000 | 805 | 3L | 20240000 | 20320000 |
| 55 | 2L | 4320000 | 4400000 | 806 | 3L | 20320000 | 20400000 |
| 56 | 2L | 4400000 | 4480000 | 807 | 3L | 20400000 | 20480000 |
| 57 | 2L | 4480000 | 4560000 | 808 | 3L | 20480000 | 20560000 |
| 58 | 2L | 4560000 | 4640000 | 809 | 3L | 20560000 | 20640000 |
| 59 | 2L | 4640000 | 4720000 | 810 | 3L | 20640000 | 20720000 |
| 60 | 2L | 4720000 | 4800000 | 811 | 3L | 20720000 | 20800000 |
| 61 | 2L | 4800000 | 4880000 | 812 | 3L | 20800000 | 20880000 |
| 62 | 2L | 4880000 | 4960000 | 813 | 3L | 20880000 | 20960000 |
| 63 | 2L | 4960000 | 5040000 | 814 | 3L | 20960000 | 21040000 |
| 64 | 2L | 5040000 | 5120000 | 815 | 3L | 21040000 | 21120000 |
| 65 | 2L | 5120000 | 5200000 | 816 | 3L | 21120000 | 21200000 |
| 66 | 2L | 5200000 | 5280000 | 817 | 3L | 21200000 | 21280000 |
| 67 | 2L | 5280000 | 5360000 | 818 | 3L | 21280000 | 21360000 |
| 68 | 2L | 5360000 | 5440000 | 819 | 3L | 21360000 | 21440000 |
| 69 | 2L | 5440000 | 5520000 | 820 | 3L | 21440000 | 21520000 |
| 70 | 2L | 5520000 | 5600000 | 821 | 3L | 21520000 | 21600000 |
| 71 | 2L | 5600000 | 5680000 | 822 | 3L | 21600000 | 21680000 |
| 72 | 2L | 5680000 | 5760000 | 823 | 3L | 21680000 | 21760000 |
| 73 | 2L | 5760000 | 5840000 | 824 | 3L | 21760000 | 21840000 |
| 74 | 2L | 5840000 | 5920000 | 825 | 3L | 21840000 | 21920000 |
| 75 | 2L | 5920000 | 6000000 | 826 | 3L | 21920000 | 22000000 |
| 76 | 2L | 6000000 | 6080000 | 827 | 3L | 22000000 | 22080000 |
| 77 | 2L | 6080000 | 6160000 | 828 | 3L | 22080000 | 22160000 |
| 78 | 2L | 6160000 | 6240000 | 829 | 3L | 22160000 | 22240000 |
| 79 | 2L | 6240000 | 6320000 | 830 | 3L | 22240000 | 22320000 |
| 80 | 2L | 6320000 | 6400000 | 831 | 3L | 22320000 | 22400000 |
| 81 | 2L | 6400000 | 6480000 | 832 | 3L | 22400000 | 22480000 |
| 82 | 2L | 6480000 | 6560000 | 833 | 3L | 22480000 | 22560000 |
| 83 | 2L | 6560000 | 6640000 | 834 | 3L | 22560000 | 22640000 |
| 84 | 2L | 6640000 | 6720000 | 835 | 3L | 22640000 | 22720000 |
| 85 | 2L | 6720000 | 6800000 | 836 | 3L | 22720000 | 22800000 |
| 86 | 2L | 6800000 | 6880000 | 837 | 3L | 22800000 | 22880000 |
| 87 | 2L | 6880000 | 6960000 | 838 | 3L | 22880000 | 22960000 |
| 88 | 2L | 6960000 | 7040000 | 839 | 3L | 22960000 | 23040000 |
| 89 | 2L | 7040000 | 7120000 | 840 | 3L | 23040000 | 23120000 |
| 90 | 2L | 7120000 | 7200000 | 841 | 3L | 23120000 | 23200000 |
| 91 | 2L | 7200000 | 7280000 | 842 | 3L | 23200000 | 23280000 |
| 92 | 2L | 7280000 | 7360000 | 843 | 3L | 23280000 | 23360000 |
| 93 | 2L | 7360000 | 7440000 | 844 | 3L | 23360000 | 23440000 |
| 94 | 2L | 7440000 | 7520000 | 845 | 3L | 23440000 | 23520000 |
| 95 | 2L | 7520000 | 7600000 | 846 | 3L | 23520000 | 23600000 |
| 96 | 2L | 7600000 | 7680000 | 847 | 3L | 23600000 | 23680000 |
| 97 | 2L | 7680000 | 7760000 | 848 | 3L | 23680000 | 23760000 |
| 98 | 2L | 7760000 | 7840000 | 849 | 3L | 23760000 | 23840000 |
| 99 | 2L | 7840000 | 7920000 | 850 | 3L | 23840000 | 23920000 |
| 100 | 2L | 7920000 | 8000000 | 851 | 3L | 23920000 | 24000000 |
| 101 | 2L | 8000000 | 8080000 | 852 | 3L | 24000000 | 24080000 |
| 102 | 2L | 8080000 | 8160000 | 853 | 3L | 24080000 | 24160000 |
| 103 | 2L | 8160000 | 8240000 | 854 | 3L | 24160000 | 24240000 |
| 104 | 2L | 8240000 | 8320000 | 855 | 3L | 24240000 | 24320000 |
| 105 | 2L | 8320000 | 8400000 | 856 | 3L | 24320000 | 24400000 |
| 106 | 2L | 8400000 | 8480000 | 857 | 3L | 24400000 | 24480000 |
| 107 | 2L | 8480000 | 8560000 | 858 | 3L | 24480000 | 24560000 |
| 108 | 2L | 8560000 | 8640000 | 859 | 3R | 0 | 80000 |
| 109 | 2L | 8640000 | 8720000 | 860 | 3R | 80000 | 160000 |
| 110 | 2L | 8720000 | 8800000 | 861 | 3R | 160000 | 240000 |
| 111 | 2L | 8800000 | 8880000 | 862 | 3R | 240000 | 320000 |
| 112 | 2L | 8880000 | 8960000 | 863 | 3R | 320000 | 400000 |
| 113 | 2L | 8960000 | 9040000 | 864 | 3R | 400000 | 480000 |
| 114 | 2L | 9040000 | 9120000 | 865 | 3R | 480000 | 560000 |
| 115 | 2L | 9120000 | 9200000 | 866 | 3R | 560000 | 640000 |
| 116 | 2L | 9200000 | 9280000 | 867 | 3R | 640000 | 720000 |
| 117 | 2L | 9280000 | 9360000 | 868 | 3R | 720000 | 800000 |
| 118 | 2L | 9360000 | 9440000 | 869 | 3R | 800000 | 880000 |
| 119 | 2L | 9440000 | 9520000 | 870 | 3R | 880000 | 960000 |
| 120 | 2L | 9520000 | 9600000 | 871 | 3R | 960000 | 1040000 |
| 121 | 2L | 9600000 | 9680000 | 872 | 3R | 1040000 | 1120000 |
| 122 | 2L | 9680000 | 9760000 | 873 | 3R | 1120000 | 1200000 |
| 123 | 2L | 9760000 | 9840000 | 874 | 3R | 1200000 | 1280000 |
| 124 | 2L | 9840000 | 9920000 | 875 | 3R | 1280000 | 1360000 |
| 125 | 2L | 9920000 | 10000000 | 876 | 3R | 1360000 | 1440000 |
| 126 | 2L | 10000000 | 10080000 | 877 | 3R | 1440000 | 1520000 |
| 127 | 2L | 10080000 | 10160000 | 878 | 3R | 1520000 | 1600000 |
| 128 | 2L | 10160000 | 10240000 | 879 | 3R | 1600000 | 1680000 |
| 129 | 2L | 10240000 | 10320000 | 880 | 3R | 1680000 | 1760000 |
| 130 | 2L | 10320000 | 10400000 | 881 | 3R | 1760000 | 1840000 |
| 131 | 2L | 10400000 | 10480000 | 882 | 3R | 1840000 | 1920000 |
| 132 | 2L | 10480000 | 10560000 | 883 | 3R | 1920000 | 2000000 |
| 133 | 2L | 10560000 | 10640000 | 884 | 3R | 2000000 | 2080000 |
| 134 | 2L | 10640000 | 10720000 | 885 | 3R | 2080000 | 2160000 |
| 135 | 2L | 10720000 | 10800000 | 886 | 3R | 2160000 | 2240000 |
| 136 | 2L | 10800000 | 10880000 | 887 | 3R | 2240000 | 2320000 |
| 137 | 2L | 10880000 | 10960000 | 888 | 3R | 2320000 | 2400000 |
| 138 | 2L | 10960000 | 11040000 | 889 | 3R | 2400000 | 2480000 |
| 139 | 2L | 11040000 | 11120000 | 890 | 3R | 2480000 | 2560000 |
| 140 | 2L | 11120000 | 11200000 | 891 | 3R | 2560000 | 2640000 |
| 141 | 2L | 11200000 | 11280000 | 892 | 3R | 2640000 | 2720000 |
| 142 | 2L | 11280000 | 11360000 | 893 | 3R | 2720000 | 2800000 |
| 143 | 2L | 11360000 | 11440000 | 894 | 3R | 2800000 | 2880000 |
| 144 | 2L | 11440000 | 11520000 | 895 | 3R | 2880000 | 2960000 |
| 145 | 2L | 11520000 | 11600000 | 896 | 3R | 2960000 | 3040000 |
| 146 | 2L | 11600000 | 11680000 | 897 | 3R | 3040000 | 3120000 |
| 147 | 2L | 11680000 | 11760000 | 898 | 3R | 3120000 | 3200000 |
| 148 | 2L | 11760000 | 11840000 | 899 | 3R | 3200000 | 3280000 |
| 149 | 2L | 11840000 | 11920000 | 900 | 3R | 3280000 | 3360000 |
| 150 | 2L | 11920000 | 12000000 | 901 | 3R | 3360000 | 3440000 |
| 151 | 2L | 12000000 | 12080000 | 902 | 3R | 3440000 | 3520000 |
| 152 | 2L | 12080000 | 12160000 | 903 | 3R | 3520000 | 3600000 |
| 153 | 2L | 12160000 | 12240000 | 904 | 3R | 3600000 | 3680000 |
| 154 | 2L | 12240000 | 12320000 | 905 | 3R | 3680000 | 3760000 |
| 155 | 2L | 12320000 | 12400000 | 906 | 3R | 3760000 | 3840000 |
| 156 | 2L | 12400000 | 12480000 | 907 | 3R | 3840000 | 3920000 |
| 157 | 2L | 12480000 | 12560000 | 908 | 3R | 3920000 | 4000000 |
| 158 | 2L | 12560000 | 12640000 | 909 | 3R | 4000000 | 4080000 |
| 159 | 2L | 12640000 | 12720000 | 910 | 3R | 4080000 | 4160000 |
| 160 | 2L | 12720000 | 12800000 | 911 | 3R | 4160000 | 4240000 |
| 161 | 2L | 12800000 | 12880000 | 912 | 3R | 4240000 | 4320000 |
| 162 | 2L | 12880000 | 12960000 | 913 | 3R | 4320000 | 4400000 |
| 163 | 2L | 12960000 | 13040000 | 914 | 3R | 4400000 | 4480000 |
| 164 | 2L | 13040000 | 13120000 | 915 | 3R | 4480000 | 4560000 |
| 165 | 2L | 13120000 | 13200000 | 916 | 3R | 4560000 | 4640000 |
| 166 | 2L | 13200000 | 13280000 | 917 | 3R | 4640000 | 4720000 |
| 167 | 2L | 13280000 | 13360000 | 918 | 3R | 4720000 | 4800000 |
| 168 | 2L | 13360000 | 13440000 | 919 | 3R | 4800000 | 4880000 |
| 169 | 2L | 13440000 | 13520000 | 920 | 3R | 4880000 | 4960000 |
| 170 | 2L | 13520000 | 13600000 | 921 | 3R | 4960000 | 5040000 |
| 171 | 2L | 13600000 | 13680000 | 922 | 3R | 5040000 | 5120000 |
| 172 | 2L | 13680000 | 13760000 | 923 | 3R | 5120000 | 5200000 |
| 173 | 2L | 13760000 | 13840000 | 924 | 3R | 5200000 | 5280000 |
| 174 | 2L | 13840000 | 13920000 | 925 | 3R | 5280000 | 5360000 |
| 175 | 2L | 13920000 | 14000000 | 926 | 3R | 5360000 | 5440000 |
| 176 | 2L | 14000000 | 14080000 | 927 | 3R | 5440000 | 5520000 |
| 177 | 2L | 14080000 | 14160000 | 928 | 3R | 5520000 | 5600000 |
| 178 | 2L | 14160000 | 14240000 | 929 | 3R | 5600000 | 5680000 |
| 179 | 2L | 14240000 | 14320000 | 930 | 3R | 5680000 | 5760000 |
| 180 | 2L | 14320000 | 14400000 | 931 | 3R | 5760000 | 5840000 |
| 181 | 2L | 14400000 | 14480000 | 932 | 3R | 5840000 | 5920000 |
| 182 | 2L | 14480000 | 14560000 | 933 | 3R | 5920000 | 6000000 |
| 183 | 2L | 14560000 | 14640000 | 934 | 3R | 6000000 | 6080000 |
| 184 | 2L | 14640000 | 14720000 | 935 | 3R | 6080000 | 6160000 |
| 185 | 2L | 14720000 | 14800000 | 936 | 3R | 6160000 | 6240000 |
| 186 | 2L | 14800000 | 14880000 | 937 | 3R | 6240000 | 6320000 |
| 187 | 2L | 14880000 | 14960000 | 938 | 3R | 6320000 | 6400000 |
| 188 | 2L | 14960000 | 15040000 | 939 | 3R | 6400000 | 6480000 |
| 189 | 2L | 15040000 | 15120000 | 940 | 3R | 6480000 | 6560000 |
| 190 | 2L | 15120000 | 15200000 | 941 | 3R | 6560000 | 6640000 |
| 191 | 2L | 15200000 | 15280000 | 942 | 3R | 6640000 | 6720000 |
| 192 | 2L | 15280000 | 15360000 | 943 | 3R | 6720000 | 6800000 |
| 193 | 2L | 15360000 | 15440000 | 944 | 3R | 6800000 | 6880000 |
| 194 | 2L | 15440000 | 15520000 | 945 | 3R | 6880000 | 6960000 |
| 195 | 2L | 15520000 | 15600000 | 946 | 3R | 6960000 | 7040000 |
| 196 | 2L | 15600000 | 15680000 | 947 | 3R | 7040000 | 7120000 |
| 197 | 2L | 15680000 | 15760000 | 948 | 3R | 7120000 | 7200000 |
| 198 | 2L | 15760000 | 15840000 | 949 | 3R | 7200000 | 7280000 |
| 199 | 2L | 15840000 | 15920000 | 950 | 3R | 7280000 | 7360000 |
| 200 | 2L | 15920000 | 16000000 | 951 | 3R | 7360000 | 7440000 |
| 201 | 2L | 16000000 | 16080000 | 952 | 3R | 7440000 | 7520000 |
| 202 | 2L | 16080000 | 16160000 | 953 | 3R | 7520000 | 7600000 |
| 203 | 2L | 16160000 | 16240000 | 954 | 3R | 7600000 | 7680000 |
| 204 | 2L | 16240000 | 16320000 | 955 | 3R | 7680000 | 7760000 |
| 205 | 2L | 16320000 | 16400000 | 956 | 3R | 7760000 | 7840000 |
| 206 | 2L | 16400000 | 16480000 | 957 | 3R | 7840000 | 7920000 |
| 207 | 2L | 16480000 | 16560000 | 958 | 3R | 7920000 | 8000000 |
| 208 | 2L | 16560000 | 16640000 | 959 | 3R | 8000000 | 8080000 |
| 209 | 2L | 16640000 | 16720000 | 960 | 3R | 8080000 | 8160000 |
| 210 | 2L | 16720000 | 16800000 | 961 | 3R | 8160000 | 8240000 |
| 211 | 2L | 16800000 | 16880000 | 962 | 3R | 8240000 | 8320000 |
| 212 | 2L | 16880000 | 16960000 | 963 | 3R | 8320000 | 8400000 |
| 213 | 2L | 16960000 | 17040000 | 964 | 3R | 8400000 | 8480000 |
| 214 | 2L | 17040000 | 17120000 | 965 | 3R | 8480000 | 8560000 |
| 215 | 2L | 17120000 | 17200000 | 966 | 3R | 8560000 | 8640000 |
| 216 | 2L | 17200000 | 17280000 | 967 | 3R | 8640000 | 8720000 |
| 217 | 2L | 17280000 | 17360000 | 968 | 3R | 8720000 | 8800000 |
| 218 | 2L | 17360000 | 17440000 | 969 | 3R | 8800000 | 8880000 |
| 219 | 2L | 17440000 | 17520000 | 970 | 3R | 8880000 | 8960000 |
| 220 | 2L | 17520000 | 17600000 | 971 | 3R | 8960000 | 9040000 |
| 221 | 2L | 17600000 | 17680000 | 972 | 3R | 9040000 | 9120000 |
| 222 | 2L | 17680000 | 17760000 | 973 | 3R | 9120000 | 9200000 |
| 223 | 2L | 17760000 | 17840000 | 974 | 3R | 9200000 | 9280000 |
| 224 | 2L | 17840000 | 17920000 | 975 | 3R | 9280000 | 9360000 |
| 225 | 2L | 17920000 | 18000000 | 976 | 3R | 9360000 | 9440000 |
| 226 | 2L | 18000000 | 18080000 | 977 | 3R | 9440000 | 9520000 |
| 227 | 2L | 18080000 | 18160000 | 978 | 3R | 9520000 | 9600000 |
| 228 | 2L | 18160000 | 18240000 | 979 | 3R | 9600000 | 9680000 |
| 229 | 2L | 18240000 | 18320000 | 980 | 3R | 9680000 | 9760000 |
| 230 | 2L | 18320000 | 18400000 | 981 | 3R | 9760000 | 9840000 |
| 231 | 2L | 18400000 | 18480000 | 982 | 3R | 9840000 | 9920000 |
| 232 | 2L | 18480000 | 18560000 | 983 | 3R | 9920000 | 10000000 |
| 233 | 2L | 18560000 | 18640000 | 984 | 3R | 10000000 | 10080000 |
| 234 | 2L | 18640000 | 18720000 | 985 | 3R | 10080000 | 10160000 |
| 235 | 2L | 18720000 | 18800000 | 986 | 3R | 10160000 | 10240000 |
| 236 | 2L | 18800000 | 18880000 | 987 | 3R | 10240000 | 10320000 |
| 237 | 2L | 18880000 | 18960000 | 988 | 3R | 10320000 | 10400000 |
| 238 | 2L | 18960000 | 19040000 | 989 | 3R | 10400000 | 10480000 |
| 239 | 2L | 19040000 | 19120000 | 990 | 3R | 10480000 | 10560000 |
| 240 | 2L | 19120000 | 19200000 | 991 | 3R | 10560000 | 10640000 |
| 241 | 2L | 19200000 | 19280000 | 992 | 3R | 10640000 | 10720000 |
| 242 | 2L | 19280000 | 19360000 | 993 | 3R | 10720000 | 10800000 |
| 243 | 2L | 19360000 | 19440000 | 994 | 3R | 10800000 | 10880000 |
| 244 | 2L | 19440000 | 19520000 | 995 | 3R | 10880000 | 10960000 |
| 245 | 2L | 19520000 | 19600000 | 996 | 3R | 10960000 | 11040000 |
| 246 | 2L | 19600000 | 19680000 | 997 | 3R | 11040000 | 11120000 |
| 247 | 2L | 19680000 | 19760000 | 998 | 3R | 11120000 | 11200000 |
| 248 | 2L | 19760000 | 19840000 | 999 | 3R | 11200000 | 11280000 |
| 249 | 2L | 19840000 | 19920000 | 1000 | 3R | 11280000 | 11360000 |
| 250 | 2L | 19920000 | 20000000 | 1001 | 3R | 11360000 | 11440000 |
| 251 | 2L | 20000000 | 20080000 | 1002 | 3R | 11440000 | 11520000 |
| 252 | 2L | 20080000 | 20160000 | 1003 | 3R | 11520000 | 11600000 |
| 253 | 2L | 20160000 | 20240000 | 1004 | 3R | 11600000 | 11680000 |
| 254 | 2L | 20240000 | 20320000 | 1005 | 3R | 11680000 | 11760000 |
| 255 | 2L | 20320000 | 20400000 | 1006 | 3R | 11760000 | 11840000 |
| 256 | 2L | 20400000 | 20480000 | 1007 | 3R | 11840000 | 11920000 |
| 257 | 2L | 20480000 | 20560000 | 1008 | 3R | 11920000 | 12000000 |
| 258 | 2L | 20560000 | 20640000 | 1009 | 3R | 12000000 | 12080000 |
| 259 | 2L | 20640000 | 20720000 | 1010 | 3R | 12080000 | 12160000 |
| 260 | 2L | 20720000 | 20800000 | 1011 | 3R | 12160000 | 12240000 |
| 261 | 2L | 20800000 | 20880000 | 1012 | 3R | 12240000 | 12320000 |
| 262 | 2L | 20880000 | 20960000 | 1013 | 3R | 12320000 | 12400000 |
| 263 | 2L | 20960000 | 21040000 | 1014 | 3R | 12400000 | 12480000 |
| 264 | 2L | 21040000 | 21120000 | 1015 | 3R | 12480000 | 12560000 |
| 265 | 2L | 21120000 | 21200000 | 1016 | 3R | 12560000 | 12640000 |
| 266 | 2L | 21200000 | 21280000 | 1017 | 3R | 12640000 | 12720000 |
| 267 | 2L | 21280000 | 21360000 | 1018 | 3R | 12720000 | 12800000 |
| 268 | 2L | 21360000 | 21440000 | 1019 | 3R | 12800000 | 12880000 |
| 269 | 2L | 21520000 | 21600000 | 1020 | 3R | 12880000 | 12960000 |
| 270 | 2L | 21600000 | 21680000 | 1021 | 3R | 12960000 | 13040000 |
| 271 | 2L | 21680000 | 21760000 | 1022 | 3R | 13040000 | 13120000 |
| 272 | 2L | 21760000 | 21840000 | 1023 | 3R | 13120000 | 13200000 |
| 273 | 2L | 21840000 | 21920000 | 1024 | 3R | 13200000 | 13280000 |
| 274 | 2L | 21920000 | 22000000 | 1025 | 3R | 13280000 | 13360000 |
| 275 | 2L | 22000000 | 22080000 | 1026 | 3R | 13360000 | 13440000 |
| 276 | 2L | 22080000 | 22160000 | 1027 | 3R | 13440000 | 13520000 |
| 277 | 2L | 22160000 | 22240000 | 1028 | 3R | 13520000 | 13600000 |
| 278 | 2L | 22240000 | 22320000 | 1029 | 3R | 13600000 | 13680000 |
| 279 | 2L | 22320000 | 22400000 | 1030 | 3R | 13680000 | 13760000 |
| 280 | 2L | 22400000 | 22480000 | 1031 | 3R | 13760000 | 13840000 |
| 281 | 2L | 22480000 | 22560000 | 1032 | 3R | 13840000 | 13920000 |
| 282 | 2L | 22560000 | 22640000 | 1033 | 3R | 13920000 | 14000000 |
| 283 | 2L | 22640000 | 22720000 | 1034 | 3R | 14000000 | 14080000 |
| 284 | 2L | 22720000 | 22800000 | 1035 | 3R | 14080000 | 14160000 |
| 285 | 2L | 22800000 | 22880000 | 1036 | 3R | 14160000 | 14240000 |
| 286 | 2L | 22880000 | 22960000 | 1037 | 3R | 14240000 | 14320000 |
| 287 | 2L | 22960000 | 23040000 | 1038 | 3R | 14320000 | 14400000 |
| 288 | 2R | 0 | 80000 | 1039 | 3R | 14400000 | 14480000 |
| 289 | 2R | 80000 | 160000 | 1040 | 3R | 14480000 | 14560000 |
| 290 | 2R | 160000 | 240000 | 1041 | 3R | 14560000 | 14640000 |
| 291 | 2R | 240000 | 320000 | 1042 | 3R | 14640000 | 14720000 |
| 292 | 2R | 320000 | 400000 | 1043 | 3R | 14720000 | 14800000 |
| 293 | 2R | 400000 | 480000 | 1044 | 3R | 14800000 | 14880000 |
| 294 | 2R | 480000 | 560000 | 1045 | 3R | 14880000 | 14960000 |
| 295 | 2R | 560000 | 640000 | 1046 | 3R | 14960000 | 15040000 |
| 296 | 2R | 640000 | 720000 | 1047 | 3R | 15040000 | 15120000 |
| 297 | 2R | 720000 | 800000 | 1048 | 3R | 15120000 | 15200000 |
| 298 | 2R | 800000 | 880000 | 1049 | 3R | 15200000 | 15280000 |
| 299 | 2R | 880000 | 960000 | 1050 | 3R | 15280000 | 15360000 |
| 300 | 2R | 960000 | 1040000 | 1051 | 3R | 15360000 | 15440000 |
| 301 | 2R | 1040000 | 1120000 | 1052 | 3R | 15440000 | 15520000 |
| 302 | 2R | 1120000 | 1200000 | 1053 | 3R | 15520000 | 15600000 |
| 303 | 2R | 1200000 | 1280000 | 1054 | 3R | 15600000 | 15680000 |
| 304 | 2R | 1280000 | 1360000 | 1055 | 3R | 15680000 | 15760000 |
| 305 | 2R | 1360000 | 1440000 | 1056 | 3R | 15760000 | 15840000 |
| 306 | 2R | 1440000 | 1520000 | 1057 | 3R | 15840000 | 15920000 |
| 307 | 2R | 1520000 | 1600000 | 1058 | 3R | 15920000 | 16000000 |
| 308 | 2R | 1600000 | 1680000 | 1059 | 3R | 16000000 | 16080000 |
| 309 | 2R | 1680000 | 1760000 | 1060 | 3R | 16080000 | 16160000 |
| 310 | 2R | 1760000 | 1840000 | 1061 | 3R | 16160000 | 16240000 |
| 311 | 2R | 1840000 | 1920000 | 1062 | 3R | 16240000 | 16320000 |
| 312 | 2R | 1920000 | 2000000 | 1063 | 3R | 16320000 | 16400000 |
| 313 | 2R | 2000000 | 2080000 | 1064 | 3R | 16400000 | 16480000 |
| 314 | 2R | 2080000 | 2160000 | 1065 | 3R | 16480000 | 16560000 |
| 315 | 2R | 2160000 | 2240000 | 1066 | 3R | 16560000 | 16640000 |
| 316 | 2R | 2320000 | 2400000 | 1067 | 3R | 16640000 | 16720000 |
| 317 | 2R | 2400000 | 2480000 | 1068 | 3R | 16720000 | 16800000 |
| 318 | 2R | 2480000 | 2560000 | 1069 | 3R | 16800000 | 16880000 |
| 319 | 2R | 2560000 | 2640000 | 1070 | 3R | 16880000 | 16960000 |
| 320 | 2R | 2640000 | 2720000 | 1071 | 3R | 16960000 | 17040000 |
| 321 | 2R | 2720000 | 2800000 | 1072 | 3R | 17040000 | 17120000 |
| 322 | 2R | 2800000 | 2880000 | 1073 | 3R | 17120000 | 17200000 |
| 323 | 2R | 2880000 | 2960000 | 1074 | 3R | 17200000 | 17280000 |
| 324 | 2R | 2960000 | 3040000 | 1075 | 3R | 17280000 | 17360000 |
| 325 | 2R | 3040000 | 3120000 | 1076 | 3R | 17360000 | 17440000 |
| 326 | 2R | 3120000 | 3200000 | 1077 | 3R | 17440000 | 17520000 |
| 327 | 2R | 3200000 | 3280000 | 1078 | 3R | 17520000 | 17600000 |
| 328 | 2R | 3280000 | 3360000 | 1079 | 3R | 17600000 | 17680000 |
| 329 | 2R | 3360000 | 3440000 | 1080 | 3R | 17680000 | 17760000 |
| 330 | 2R | 3440000 | 3520000 | 1081 | 3R | 17760000 | 17840000 |
| 331 | 2R | 3520000 | 3600000 | 1082 | 3R | 17840000 | 17920000 |
| 332 | 2R | 3600000 | 3680000 | 1083 | 3R | 17920000 | 18000000 |
| 333 | 2R | 3680000 | 3760000 | 1084 | 3R | 18000000 | 18080000 |
| 334 | 2R | 3760000 | 3840000 | 1085 | 3R | 18080000 | 18160000 |
| 335 | 2R | 3840000 | 3920000 | 1086 | 3R | 18160000 | 18240000 |
| 336 | 2R | 3920000 | 4000000 | 1087 | 3R | 18240000 | 18320000 |
| 337 | 2R | 4000000 | 4080000 | 1088 | 3R | 18320000 | 18400000 |
| 338 | 2R | 4080000 | 4160000 | 1089 | 3R | 18400000 | 18480000 |
| 339 | 2R | 4160000 | 4240000 | 1090 | 3R | 18480000 | 18560000 |
| 340 | 2R | 4240000 | 4320000 | 1091 | 3R | 18560000 | 18640000 |
| 341 | 2R | 4320000 | 4400000 | 1092 | 3R | 18640000 | 18720000 |
| 342 | 2R | 4400000 | 4480000 | 1093 | 3R | 18720000 | 18800000 |
| 343 | 2R | 4480000 | 4560000 | 1094 | 3R | 18800000 | 18880000 |
| 344 | 2R | 4560000 | 4640000 | 1095 | 3R | 18880000 | 18960000 |
| 345 | 2R | 4640000 | 4720000 | 1096 | 3R | 18960000 | 19040000 |
| 346 | 2R | 4720000 | 4800000 | 1097 | 3R | 19040000 | 19120000 |
| 347 | 2R | 4800000 | 4880000 | 1098 | 3R | 19120000 | 19200000 |
| 348 | 2R | 4880000 | 4960000 | 1099 | 3R | 19200000 | 19280000 |
| 349 | 2R | 4960000 | 5040000 | 1100 | 3R | 19280000 | 19360000 |
| 350 | 2R | 5040000 | 5120000 | 1101 | 3R | 19360000 | 19440000 |
| 351 | 2R | 5120000 | 5200000 | 1102 | 3R | 19440000 | 19520000 |
| 352 | 2R | 5200000 | 5280000 | 1103 | 3R | 19520000 | 19600000 |
| 353 | 2R | 5280000 | 5360000 | 1104 | 3R | 19600000 | 19680000 |
| 354 | 2R | 5360000 | 5440000 | 1105 | 3R | 19680000 | 19760000 |
| 355 | 2R | 5440000 | 5520000 | 1106 | 3R | 19760000 | 19840000 |
| 356 | 2R | 5520000 | 5600000 | 1107 | 3R | 19840000 | 19920000 |
| 357 | 2R | 5600000 | 5680000 | 1108 | 3R | 19920000 | 20000000 |
| 358 | 2R | 5680000 | 5760000 | 1109 | 3R | 20000000 | 20080000 |
| 359 | 2R | 5760000 | 5840000 | 1110 | 3R | 20080000 | 20160000 |
| 360 | 2R | 5840000 | 5920000 | 1111 | 3R | 20160000 | 20240000 |
| 361 | 2R | 5920000 | 6000000 | 1112 | 3R | 20240000 | 20320000 |
| 362 | 2R | 6000000 | 6080000 | 1113 | 3R | 20320000 | 20400000 |
| 363 | 2R | 6080000 | 6160000 | 1114 | 3R | 20400000 | 20480000 |
| 364 | 2R | 6160000 | 6240000 | 1115 | 3R | 20480000 | 20560000 |
| 365 | 2R | 6240000 | 6320000 | 1116 | 3R | 20560000 | 20640000 |
| 366 | 2R | 6320000 | 6400000 | 1117 | 3R | 20640000 | 20720000 |
| 367 | 2R | 6400000 | 6480000 | 1118 | 3R | 20720000 | 20800000 |
| 368 | 2R | 6480000 | 6560000 | 1119 | 3R | 20800000 | 20880000 |
| 369 | 2R | 6560000 | 6640000 | 1120 | 3R | 20880000 | 20960000 |
| 370 | 2R | 6640000 | 6720000 | 1121 | 3R | 20960000 | 21040000 |
| 371 | 2R | 6720000 | 6800000 | 1122 | 3R | 21040000 | 21120000 |
| 372 | 2R | 6800000 | 6880000 | 1123 | 3R | 21120000 | 21200000 |
| 373 | 2R | 6880000 | 6960000 | 1124 | 3R | 21200000 | 21280000 |
| 374 | 2R | 6960000 | 7040000 | 1125 | 3R | 21280000 | 21360000 |
| 375 | 2R | 7040000 | 7120000 | 1126 | 3R | 21360000 | 21440000 |
| 376 | 2R | 7120000 | 7200000 | 1127 | 3R | 21440000 | 21520000 |
| 377 | 2R | 7200000 | 7280000 | 1128 | 3R | 21520000 | 21600000 |
| 378 | 2R | 7280000 | 7360000 | 1129 | 3R | 21600000 | 21680000 |
| 379 | 2R | 7360000 | 7440000 | 1130 | 3R | 21680000 | 21760000 |
| 380 | 2R | 7440000 | 7520000 | 1131 | 3R | 21760000 | 21840000 |
| 381 | 2R | 7520000 | 7600000 | 1132 | 3R | 21840000 | 21920000 |
| 382 | 2R | 7600000 | 7680000 | 1133 | 3R | 21920000 | 22000000 |
| 383 | 2R | 7680000 | 7760000 | 1134 | 3R | 22000000 | 22080000 |
| 384 | 2R | 7760000 | 7840000 | 1135 | 3R | 22080000 | 22160000 |
| 385 | 2R | 7840000 | 7920000 | 1136 | 3R | 22160000 | 22240000 |
| 386 | 2R | 7920000 | 8000000 | 1137 | 3R | 22240000 | 22320000 |
| 387 | 2R | 8000000 | 8080000 | 1138 | 3R | 22320000 | 22400000 |
| 388 | 2R | 8080000 | 8160000 | 1139 | 3R | 22400000 | 22480000 |
| 389 | 2R | 8160000 | 8240000 | 1140 | 3R | 22480000 | 22560000 |
| 390 | 2R | 8240000 | 8320000 | 1141 | 3R | 22560000 | 22640000 |
| 391 | 2R | 8320000 | 8400000 | 1142 | 3R | 22640000 | 22720000 |
| 392 | 2R | 8400000 | 8480000 | 1143 | 3R | 22720000 | 22800000 |
| 393 | 2R | 8480000 | 8560000 | 1144 | 3R | 22800000 | 22880000 |
| 394 | 2R | 8560000 | 8640000 | 1145 | 3R | 22880000 | 22960000 |
| 395 | 2R | 8640000 | 8720000 | 1146 | 3R | 22960000 | 23040000 |
| 396 | 2R | 8720000 | 8800000 | 1147 | 3R | 23040000 | 23120000 |
| 397 | 2R | 8800000 | 8880000 | 1148 | 3R | 23120000 | 23200000 |
| 398 | 2R | 8880000 | 8960000 | 1149 | 3R | 23200000 | 23280000 |
| 399 | 2R | 8960000 | 9040000 | 1150 | 3R | 23280000 | 23360000 |
| 400 | 2R | 9040000 | 9120000 | 1151 | 3R | 23360000 | 23440000 |
| 401 | 2R | 9120000 | 9200000 | 1152 | 3R | 23440000 | 23520000 |
| 402 | 2R | 9200000 | 9280000 | 1153 | 3R | 23520000 | 23600000 |
| 403 | 2R | 9280000 | 9360000 | 1154 | 3R | 23600000 | 23680000 |
| 404 | 2R | 9360000 | 9440000 | 1155 | 3R | 23680000 | 23760000 |
| 405 | 2R | 9440000 | 9520000 | 1156 | 3R | 23760000 | 23840000 |
| 406 | 2R | 9520000 | 9600000 | 1157 | 3R | 23840000 | 23920000 |
| 407 | 2R | 9600000 | 9680000 | 1158 | 3R | 23920000 | 24000000 |
| 408 | 2R | 9680000 | 9760000 | 1159 | 3R | 24000000 | 24080000 |
| 409 | 2R | 9760000 | 9840000 | 1160 | 3R | 24080000 | 24160000 |
| 410 | 2R | 9840000 | 9920000 | 1161 | 3R | 24160000 | 24240000 |
| 411 | 2R | 9920000 | 10000000 | 1162 | 3R | 24240000 | 24320000 |
| 412 | 2R | 10000000 | 10080000 | 1163 | 3R | 24320000 | 24400000 |
| 413 | 2R | 10080000 | 10160000 | 1164 | 3R | 24400000 | 24480000 |
| 414 | 2R | 10160000 | 10240000 | 1165 | 3R | 24480000 | 24560000 |
| 415 | 2R | 10240000 | 10320000 | 1166 | 3R | 24560000 | 24640000 |
| 416 | 2R | 10320000 | 10400000 | 1167 | 3R | 24640000 | 24720000 |
| 417 | 2R | 10400000 | 10480000 | 1168 | 3R | 24720000 | 24800000 |
| 418 | 2R | 10480000 | 10560000 | 1169 | 3R | 24800000 | 24880000 |
| 419 | 2R | 10560000 | 10640000 | 1170 | 3R | 24880000 | 24960000 |
| 420 | 2R | 10640000 | 10720000 | 1171 | 3R | 24960000 | 25040000 |
| 421 | 2R | 10720000 | 10800000 | 1172 | 3R | 25040000 | 25120000 |
| 422 | 2R | 10800000 | 10880000 | 1173 | 3R | 25120000 | 25200000 |
| 423 | 2R | 10880000 | 10960000 | 1174 | 3R | 25200000 | 25280000 |
| 424 | 2R | 10960000 | 11040000 | 1175 | 3R | 25280000 | 25360000 |
| 425 | 2R | 11040000 | 11120000 | 1176 | 3R | 25360000 | 25440000 |
| 426 | 2R | 11120000 | 11200000 | 1177 | 3R | 25440000 | 25520000 |
| 427 | 2R | 11200000 | 11280000 | 1178 | 3R | 25520000 | 25600000 |
| 428 | 2R | 11280000 | 11360000 | 1179 | 3R | 25600000 | 25680000 |
| 429 | 2R | 11360000 | 11440000 | 1180 | 3R | 25680000 | 25760000 |
| 430 | 2R | 11440000 | 11520000 | 1181 | 3R | 25760000 | 25840000 |
| 431 | 2R | 11520000 | 11600000 | 1182 | 3R | 25840000 | 25920000 |
| 432 | 2R | 11600000 | 11680000 | 1183 | 3R | 25920000 | 26000000 |
| 433 | 2R | 11680000 | 11760000 | 1184 | 3R | 26000000 | 26080000 |
| 434 | 2R | 11760000 | 11840000 | 1185 | 3R | 26080000 | 26160000 |
| 435 | 2R | 11840000 | 11920000 | 1186 | 3R | 26160000 | 26240000 |
| 436 | 2R | 11920000 | 12000000 | 1187 | 3R | 26240000 | 26320000 |
| 437 | 2R | 12000000 | 12080000 | 1188 | 3R | 26320000 | 26400000 |
| 438 | 2R | 12080000 | 12160000 | 1189 | 3R | 26400000 | 26480000 |
| 439 | 2R | 12160000 | 12240000 | 1190 | 3R | 26480000 | 26560000 |
| 440 | 2R | 12240000 | 12320000 | 1191 | 3R | 26560000 | 26640000 |
| 441 | 2R | 12320000 | 12400000 | 1192 | 3R | 26640000 | 26720000 |
| 442 | 2R | 12400000 | 12480000 | 1193 | 3R | 26720000 | 26800000 |
| 443 | 2R | 12480000 | 12560000 | 1194 | 3R | 26800000 | 26880000 |
| 444 | 2R | 12560000 | 12640000 | 1195 | 3R | 26880000 | 26960000 |
| 445 | 2R | 12640000 | 12720000 | 1196 | 3R | 26960000 | 27040000 |
| 446 | 2R | 12720000 | 12800000 | 1197 | 3R | 27040000 | 27120000 |
| 447 | 2R | 12800000 | 12880000 | 1198 | 3R | 27120000 | 27200000 |
| 448 | 2R | 12880000 | 12960000 | 1199 | 3R | 27200000 | 27280000 |
| 449 | 2R | 12960000 | 13040000 | 1200 | 3R | 27280000 | 27360000 |
| 450 | 2R | 13040000 | 13120000 | 1201 | 3R | 27360000 | 27440000 |
| 451 | 2R | 13120000 | 13200000 | 1202 | 3R | 27440000 | 27520000 |
| 452 | 2R | 13200000 | 13280000 | 1203 | 3R | 27520000 | 27600000 |
| 453 | 2R | 13280000 | 13360000 | 1204 | 3R | 27600000 | 27680000 |
| 454 | 2R | 13360000 | 13440000 | 1205 | 3R | 27680000 | 27760000 |
| 455 | 2R | 13440000 | 13520000 | 1206 | 3R | 27760000 | 27840000 |
| 456 | 2R | 13520000 | 13600000 | 1207 | 3R | 27840000 | 27920000 |
| 457 | 2R | 13600000 | 13680000 | 1208 | 4 | 0 | 80000 |
| 458 | 2R | 13680000 | 13760000 | 1209 | 4 | 80000 | 160000 |
| 459 | 2R | 13760000 | 13840000 | 1210 | 4 | 160000 | 240000 |
| 460 | 2R | 13840000 | 13920000 | 1211 | 4 | 240000 | 320000 |
| 461 | 2R | 13920000 | 14000000 | 1212 | 4 | 320000 | 400000 |
| 462 | 2R | 14000000 | 14080000 | 1213 | 4 | 400000 | 480000 |
| 463 | 2R | 14080000 | 14160000 | 1214 | 4 | 480000 | 560000 |
| 464 | 2R | 14160000 | 14240000 | 1215 | 4 | 560000 | 640000 |
| 465 | 2R | 14240000 | 14320000 | 1216 | 4 | 640000 | 720000 |
| 466 | 2R | 14320000 | 14400000 | 1217 | 4 | 720000 | 800000 |
| 467 | 2R | 14400000 | 14480000 | 1218 | 4 | 800000 | 880000 |
| 468 | 2R | 14480000 | 14560000 | 1219 | 4 | 880000 | 960000 |
| 469 | 2R | 14560000 | 14640000 | 1220 | 4 | 960000 | 1040000 |
| 470 | 2R | 14640000 | 14720000 | 1221 | 4 | 1040000 | 1120000 |
| 471 | 2R | 14720000 | 14800000 | 1222 | 4 | 1120000 | 1200000 |
| 472 | 2R | 14800000 | 14880000 | 1223 | 4 | 1200000 | 1280000 |
| 473 | 2R | 14880000 | 14960000 | 1224 | X | 0 | 80000 |
| 474 | 2R | 14960000 | 15040000 | 1225 | X | 80000 | 160000 |
| 475 | 2R | 15040000 | 15120000 | 1226 | X | 160000 | 240000 |
| 476 | 2R | 15120000 | 15200000 | 1227 | X | 240000 | 320000 |
| 477 | 2R | 15200000 | 15280000 | 1228 | X | 320000 | 400000 |
| 478 | 2R | 15280000 | 15360000 | 1229 | X | 400000 | 480000 |
| 479 | 2R | 15360000 | 15440000 | 1230 | X | 480000 | 560000 |
| 480 | 2R | 15440000 | 15520000 | 1231 | X | 560000 | 640000 |
| 481 | 2R | 15520000 | 15600000 | 1232 | X | 640000 | 720000 |
| 482 | 2R | 15600000 | 15680000 | 1233 | X | 720000 | 800000 |
| 483 | 2R | 15680000 | 15760000 | 1234 | X | 800000 | 880000 |
| 484 | 2R | 15760000 | 15840000 | 1235 | X | 880000 | 960000 |
| 485 | 2R | 15840000 | 15920000 | 1236 | X | 960000 | 1040000 |
| 486 | 2R | 15920000 | 16000000 | 1237 | X | 1040000 | 1120000 |
| 487 | 2R | 16000000 | 16080000 | 1238 | X | 1120000 | 1200000 |
| 488 | 2R | 16080000 | 16160000 | 1239 | X | 1200000 | 1280000 |
| 489 | 2R | 16160000 | 16240000 | 1240 | X | 1280000 | 1360000 |
| 490 | 2R | 16240000 | 16320000 | 1241 | X | 1360000 | 1440000 |
| 491 | 2R | 16320000 | 16400000 | 1242 | X | 1440000 | 1520000 |
| 492 | 2R | 16400000 | 16480000 | 1243 | X | 1520000 | 1600000 |
| 493 | 2R | 16480000 | 16560000 | 1244 | X | 1600000 | 1680000 |
| 494 | 2R | 16560000 | 16640000 | 1245 | X | 1680000 | 1760000 |
| 495 | 2R | 16640000 | 16720000 | 1246 | X | 1760000 | 1840000 |
| 496 | 2R | 16720000 | 16800000 | 1247 | X | 1840000 | 1920000 |
| 497 | 2R | 16800000 | 16880000 | 1248 | X | 1920000 | 2000000 |
| 498 | 2R | 16880000 | 16960000 | 1249 | X | 2000000 | 2080000 |
| 499 | 2R | 16960000 | 17040000 | 1250 | X | 2080000 | 2160000 |
| 500 | 2R | 17040000 | 17120000 | 1251 | X | 2160000 | 2240000 |
| 501 | 2R | 17120000 | 17200000 | 1252 | X | 2240000 | 2320000 |
| 502 | 2R | 17200000 | 17280000 | 1253 | X | 2320000 | 2400000 |
| 503 | 2R | 17280000 | 17360000 | 1254 | X | 2400000 | 2480000 |
| 504 | 2R | 17360000 | 17440000 | 1255 | X | 2480000 | 2560000 |
| 505 | 2R | 17440000 | 17520000 | 1256 | X | 2560000 | 2640000 |
| 506 | 2R | 17520000 | 17600000 | 1257 | X | 2640000 | 2720000 |
| 507 | 2R | 17600000 | 17680000 | 1258 | X | 2720000 | 2800000 |
| 508 | 2R | 17680000 | 17760000 | 1259 | X | 2800000 | 2880000 |
| 509 | 2R | 17760000 | 17840000 | 1260 | X | 2880000 | 2960000 |
| 510 | 2R | 17840000 | 17920000 | 1261 | X | 2960000 | 3040000 |
| 511 | 2R | 17920000 | 18000000 | 1262 | X | 3040000 | 3120000 |
| 512 | 2R | 18000000 | 18080000 | 1263 | X | 3120000 | 3200000 |
| 513 | 2R | 18080000 | 18160000 | 1264 | X | 3200000 | 3280000 |
| 514 | 2R | 18160000 | 18240000 | 1265 | X | 3280000 | 3360000 |
| 515 | 2R | 18240000 | 18320000 | 1266 | X | 3360000 | 3440000 |
| 516 | 2R | 18320000 | 18400000 | 1267 | X | 3440000 | 3520000 |
| 517 | 2R | 18400000 | 18480000 | 1268 | X | 3520000 | 3600000 |
| 518 | 2R | 18480000 | 18560000 | 1269 | X | 3600000 | 3680000 |
| 519 | 2R | 18560000 | 18640000 | 1270 | X | 3680000 | 3760000 |
| 520 | 2R | 18640000 | 18720000 | 1271 | X | 3760000 | 3840000 |
| 521 | 2R | 18720000 | 18800000 | 1272 | X | 3840000 | 3920000 |
| 522 | 2R | 18800000 | 18880000 | 1273 | X | 3920000 | 4000000 |
| 523 | 2R | 18880000 | 18960000 | 1274 | X | 4000000 | 4080000 |
| 524 | 2R | 18960000 | 19040000 | 1275 | X | 4080000 | 4160000 |
| 525 | 2R | 19040000 | 19120000 | 1276 | X | 4160000 | 4240000 |
| 526 | 2R | 19120000 | 19200000 | 1277 | X | 4240000 | 4320000 |
| 527 | 2R | 19200000 | 19280000 | 1278 | X | 4320000 | 4400000 |
| 528 | 2R | 19280000 | 19360000 | 1279 | X | 4400000 | 4480000 |
| 529 | 2R | 19360000 | 19440000 | 1280 | X | 4480000 | 4560000 |
| 530 | 2R | 19440000 | 19520000 | 1281 | X | 4560000 | 4640000 |
| 531 | 2R | 19520000 | 19600000 | 1282 | X | 4640000 | 4720000 |
| 532 | 2R | 19600000 | 19680000 | 1283 | X | 4720000 | 4800000 |
| 533 | 2R | 19680000 | 19760000 | 1284 | X | 4800000 | 4880000 |
| 534 | 2R | 19760000 | 19840000 | 1285 | X | 4880000 | 4960000 |
| 535 | 2R | 19840000 | 19920000 | 1286 | X | 4960000 | 5040000 |
| 536 | 2R | 19920000 | 20000000 | 1287 | X | 5040000 | 5120000 |
| 537 | 2R | 20000000 | 20080000 | 1288 | X | 5120000 | 5200000 |
| 538 | 2R | 20080000 | 20160000 | 1289 | X | 5200000 | 5280000 |
| 539 | 2R | 20160000 | 20240000 | 1290 | X | 5280000 | 5360000 |
| 540 | 2R | 20240000 | 20320000 | 1291 | X | 5360000 | 5440000 |
| 541 | 2R | 20320000 | 20400000 | 1292 | X | 5440000 | 5520000 |
| 542 | 2R | 20400000 | 20480000 | 1293 | X | 5520000 | 5600000 |
| 543 | 2R | 20480000 | 20560000 | 1294 | X | 5600000 | 5680000 |
| 544 | 2R | 20560000 | 20640000 | 1295 | X | 5680000 | 5760000 |
| 545 | 2R | 20640000 | 20720000 | 1296 | X | 5760000 | 5840000 |
| 546 | 2R | 20720000 | 20800000 | 1297 | X | 5840000 | 5920000 |
| 547 | 2R | 20800000 | 20880000 | 1298 | X | 5920000 | 6000000 |
| 548 | 2R | 20880000 | 20960000 | 1299 | X | 6000000 | 6080000 |
| 549 | 2R | 20960000 | 21040000 | 1300 | X | 6080000 | 6160000 |
| 550 | 2R | 21040000 | 21120000 | 1301 | X | 6160000 | 6240000 |
| 551 | 2R | 21120000 | 21200000 | 1302 | X | 6240000 | 6320000 |
| 552 | 3L | 0 | 80000 | 1303 | X | 6320000 | 6400000 |
| 553 | 3L | 80000 | 160000 | 1304 | X | 6400000 | 6480000 |
| 554 | 3L | 160000 | 240000 | 1305 | X | 6480000 | 6560000 |
| 555 | 3L | 240000 | 320000 | 1306 | X | 6560000 | 6640000 |
| 556 | 3L | 320000 | 400000 | 1307 | X | 6640000 | 6720000 |
| 557 | 3L | 400000 | 480000 | 1308 | X | 6720000 | 6800000 |
| 558 | 3L | 480000 | 560000 | 1309 | X | 6800000 | 6880000 |
| 559 | 3L | 560000 | 640000 | 1310 | X | 6880000 | 6960000 |
| 560 | 3L | 640000 | 720000 | 1311 | X | 6960000 | 7040000 |
| 561 | 3L | 720000 | 800000 | 1312 | X | 7040000 | 7120000 |
| 562 | 3L | 800000 | 880000 | 1313 | X | 7120000 | 7200000 |
| 563 | 3L | 880000 | 960000 | 1314 | X | 7200000 | 7280000 |
| 564 | 3L | 960000 | 1040000 | 1315 | X | 7280000 | 7360000 |
| 565 | 3L | 1040000 | 1120000 | 1316 | X | 7360000 | 7440000 |
| 566 | 3L | 1120000 | 1200000 | 1317 | X | 7440000 | 7520000 |
| 567 | 3L | 1200000 | 1280000 | 1318 | X | 7520000 | 7600000 |
| 568 | 3L | 1280000 | 1360000 | 1319 | X | 7600000 | 7680000 |
| 569 | 3L | 1360000 | 1440000 | 1320 | X | 7680000 | 7760000 |
| 570 | 3L | 1440000 | 1520000 | 1321 | X | 7760000 | 7840000 |
| 571 | 3L | 1520000 | 1600000 | 1322 | X | 7840000 | 7920000 |
| 572 | 3L | 1600000 | 1680000 | 1323 | X | 7920000 | 8000000 |
| 573 | 3L | 1680000 | 1760000 | 1324 | X | 8000000 | 8080000 |
| 574 | 3L | 1760000 | 1840000 | 1325 | X | 8080000 | 8160000 |
| 575 | 3L | 1840000 | 1920000 | 1326 | X | 8160000 | 8240000 |
| 576 | 3L | 1920000 | 2000000 | 1327 | X | 8240000 | 8320000 |
| 577 | 3L | 2000000 | 2080000 | 1328 | X | 8320000 | 8400000 |
| 578 | 3L | 2080000 | 2160000 | 1329 | X | 8400000 | 8480000 |
| 579 | 3L | 2160000 | 2240000 | 1330 | X | 8480000 | 8560000 |
| 580 | 3L | 2240000 | 2320000 | 1331 | X | 8560000 | 8640000 |
| 581 | 3L | 2320000 | 2400000 | 1332 | X | 8640000 | 8720000 |
| 582 | 3L | 2400000 | 2480000 | 1333 | X | 8720000 | 8800000 |
| 583 | 3L | 2480000 | 2560000 | 1334 | X | 8800000 | 8880000 |
| 584 | 3L | 2560000 | 2640000 | 1335 | X | 8880000 | 8960000 |
| 585 | 3L | 2640000 | 2720000 | 1336 | X | 8960000 | 9040000 |
| 586 | 3L | 2720000 | 2800000 | 1337 | X | 9040000 | 9120000 |
| 587 | 3L | 2800000 | 2880000 | 1338 | X | 9120000 | 9200000 |
| 588 | 3L | 2880000 | 2960000 | 1339 | X | 9200000 | 9280000 |
| 589 | 3L | 2960000 | 3040000 | 1340 | X | 9280000 | 9360000 |
| 590 | 3L | 3040000 | 3120000 | 1341 | X | 9360000 | 9440000 |
| 591 | 3L | 3120000 | 3200000 | 1342 | X | 9440000 | 9520000 |
| 592 | 3L | 3200000 | 3280000 | 1343 | X | 9520000 | 9600000 |
| 593 | 3L | 3280000 | 3360000 | 1344 | X | 9600000 | 9680000 |
| 594 | 3L | 3360000 | 3440000 | 1345 | X | 9680000 | 9760000 |
| 595 | 3L | 3440000 | 3520000 | 1346 | X | 9760000 | 9840000 |
| 596 | 3L | 3520000 | 3600000 | 1347 | X | 9840000 | 9920000 |
| 597 | 3L | 3600000 | 3680000 | 1348 | X | 9920000 | 10000000 |
| 598 | 3L | 3680000 | 3760000 | 1349 | X | 10000000 | 10080000 |
| 599 | 3L | 3760000 | 3840000 | 1350 | X | 10080000 | 10160000 |
| 600 | 3L | 3840000 | 3920000 | 1351 | X | 10160000 | 10240000 |
| 601 | 3L | 3920000 | 4000000 | 1352 | X | 10240000 | 10320000 |
| 602 | 3L | 4000000 | 4080000 | 1353 | X | 10320000 | 10400000 |
| 603 | 3L | 4080000 | 4160000 | 1354 | X | 10400000 | 10480000 |
| 604 | 3L | 4160000 | 4240000 | 1355 | X | 10480000 | 10560000 |
| 605 | 3L | 4240000 | 4320000 | 1356 | X | 10560000 | 10640000 |
| 606 | 3L | 4320000 | 4400000 | 1357 | X | 10640000 | 10720000 |
| 607 | 3L | 4400000 | 4480000 | 1358 | X | 10720000 | 10800000 |
| 608 | 3L | 4480000 | 4560000 | 1359 | X | 10800000 | 10880000 |
| 609 | 3L | 4560000 | 4640000 | 1360 | X | 10880000 | 10960000 |
| 610 | 3L | 4640000 | 4720000 | 1361 | X | 10960000 | 11040000 |
| 611 | 3L | 4720000 | 4800000 | 1362 | X | 11040000 | 11120000 |
| 612 | 3L | 4800000 | 4880000 | 1363 | X | 11120000 | 11200000 |
| 613 | 3L | 4880000 | 4960000 | 1364 | X | 11200000 | 11280000 |
| 614 | 3L | 4960000 | 5040000 | 1365 | X | 11280000 | 11360000 |
| 615 | 3L | 5040000 | 5120000 | 1366 | X | 11360000 | 11440000 |
| 616 | 3L | 5120000 | 5200000 | 1367 | X | 11440000 | 11520000 |
| 617 | 3L | 5200000 | 5280000 | 1368 | X | 11520000 | 11600000 |
| 618 | 3L | 5280000 | 5360000 | 1369 | X | 11600000 | 11680000 |
| 619 | 3L | 5360000 | 5440000 | 1370 | X | 11680000 | 11760000 |
| 620 | 3L | 5440000 | 5520000 | 1371 | X | 11760000 | 11840000 |
| 621 | 3L | 5520000 | 5600000 | 1372 | X | 11840000 | 11920000 |
| 622 | 3L | 5600000 | 5680000 | 1373 | X | 11920000 | 12000000 |
| 623 | 3L | 5680000 | 5760000 | 1374 | X | 12000000 | 12080000 |
| 624 | 3L | 5760000 | 5840000 | 1375 | X | 12080000 | 12160000 |
| 625 | 3L | 5840000 | 5920000 | 1376 | X | 12160000 | 12240000 |
| 626 | 3L | 5920000 | 6000000 | 1377 | X | 12240000 | 12320000 |
| 627 | 3L | 6000000 | 6080000 | 1378 | X | 12320000 | 12400000 |
| 628 | 3L | 6080000 | 6160000 | 1379 | X | 12400000 | 12480000 |
| 629 | 3L | 6160000 | 6240000 | 1380 | X | 12480000 | 12560000 |
| 630 | 3L | 6240000 | 6320000 | 1381 | X | 12560000 | 12640000 |
| 631 | 3L | 6320000 | 6400000 | 1382 | X | 12640000 | 12720000 |
| 632 | 3L | 6400000 | 6480000 | 1383 | X | 12720000 | 12800000 |
| 633 | 3L | 6480000 | 6560000 | 1384 | X | 12800000 | 12880000 |
| 634 | 3L | 6560000 | 6640000 | 1385 | X | 12880000 | 12960000 |
| 635 | 3L | 6640000 | 6720000 | 1386 | X | 12960000 | 13040000 |
| 636 | 3L | 6720000 | 6800000 | 1387 | X | 13040000 | 13120000 |
| 637 | 3L | 6800000 | 6880000 | 1388 | X | 13120000 | 13200000 |
| 638 | 3L | 6880000 | 6960000 | 1389 | X | 13200000 | 13280000 |
| 639 | 3L | 6960000 | 7040000 | 1390 | X | 13280000 | 13360000 |
| 640 | 3L | 7040000 | 7120000 | 1391 | X | 13360000 | 13440000 |
| 641 | 3L | 7120000 | 7200000 | 1392 | X | 13440000 | 13520000 |
| 642 | 3L | 7200000 | 7280000 | 1393 | X | 13520000 | 13600000 |
| 643 | 3L | 7280000 | 7360000 | 1394 | X | 13600000 | 13680000 |
| 644 | 3L | 7360000 | 7440000 | 1395 | X | 13680000 | 13760000 |
| 645 | 3L | 7440000 | 7520000 | 1396 | X | 13760000 | 13840000 |
| 646 | 3L | 7520000 | 7600000 | 1397 | X | 13840000 | 13920000 |
| 647 | 3L | 7600000 | 7680000 | 1398 | X | 13920000 | 14000000 |
| 648 | 3L | 7680000 | 7760000 | 1399 | X | 14000000 | 14080000 |
| 649 | 3L | 7760000 | 7840000 | 1400 | X | 14080000 | 14160000 |
| 650 | 3L | 7840000 | 7920000 | 1401 | X | 14160000 | 14240000 |
| 651 | 3L | 7920000 | 8000000 | 1402 | X | 14240000 | 14320000 |
| 652 | 3L | 8000000 | 8080000 | 1403 | X | 14320000 | 14400000 |
| 653 | 3L | 8080000 | 8160000 | 1404 | X | 14400000 | 14480000 |
| 654 | 3L | 8160000 | 8240000 | 1405 | X | 14480000 | 14560000 |
| 655 | 3L | 8240000 | 8320000 | 1406 | X | 14560000 | 14640000 |
| 656 | 3L | 8320000 | 8400000 | 1407 | X | 14640000 | 14720000 |
| 657 | 3L | 8400000 | 8480000 | 1408 | X | 14720000 | 14800000 |
| 658 | 3L | 8480000 | 8560000 | 1409 | X | 14800000 | 14880000 |
| 659 | 3L | 8560000 | 8640000 | 1410 | X | 14880000 | 14960000 |
| 660 | 3L | 8640000 | 8720000 | 1411 | X | 14960000 | 15040000 |
| 661 | 3L | 8720000 | 8800000 | 1412 | X | 15040000 | 15120000 |
| 662 | 3L | 8800000 | 8880000 | 1413 | X | 15120000 | 15200000 |
| 663 | 3L | 8880000 | 8960000 | 1414 | X | 15200000 | 15280000 |
| 664 | 3L | 8960000 | 9040000 | 1415 | X | 15280000 | 15360000 |
| 665 | 3L | 9040000 | 9120000 | 1416 | X | 15360000 | 15440000 |
| 666 | 3L | 9120000 | 9200000 | 1417 | X | 15440000 | 15520000 |
| 667 | 3L | 9200000 | 9280000 | 1418 | X | 15520000 | 15600000 |
| 668 | 3L | 9280000 | 9360000 | 1419 | X | 15600000 | 15680000 |
| 669 | 3L | 9360000 | 9440000 | 1420 | X | 15680000 | 15760000 |
| 670 | 3L | 9440000 | 9520000 | 1421 | X | 15760000 | 15840000 |
| 671 | 3L | 9520000 | 9600000 | 1422 | X | 15840000 | 15920000 |
| 672 | 3L | 9600000 | 9680000 | 1423 | X | 15920000 | 16000000 |
| 673 | 3L | 9680000 | 9760000 | 1424 | X | 16000000 | 16080000 |
| 674 | 3L | 9760000 | 9840000 | 1425 | X | 16080000 | 16160000 |
| 675 | 3L | 9840000 | 9920000 | 1426 | X | 16160000 | 16240000 |
| 676 | 3L | 9920000 | 10000000 | 1427 | X | 16240000 | 16320000 |
| 677 | 3L | 10000000 | 10080000 | 1428 | X | 16320000 | 16400000 |
| 678 | 3L | 10080000 | 10160000 | 1429 | X | 16400000 | 16480000 |
| 679 | 3L | 10160000 | 10240000 | 1430 | X | 16480000 | 16560000 |
| 680 | 3L | 10240000 | 10320000 | 1431 | X | 16560000 | 16640000 |
| 681 | 3L | 10320000 | 10400000 | 1432 | X | 16640000 | 16720000 |
| 682 | 3L | 10400000 | 10480000 | 1433 | X | 16720000 | 16800000 |
| 683 | 3L | 10480000 | 10560000 | 1434 | X | 16800000 | 16880000 |
| 684 | 3L | 10560000 | 10640000 | 1435 | X | 16880000 | 16960000 |
| 685 | 3L | 10640000 | 10720000 | 1436 | X | 16960000 | 17040000 |
| 686 | 3L | 10720000 | 10800000 | 1437 | X | 17040000 | 17120000 |
| 687 | 3L | 10800000 | 10880000 | 1438 | X | 17120000 | 17200000 |
| 688 | 3L | 10880000 | 10960000 | 1439 | X | 17200000 | 17280000 |
| 689 | 3L | 10960000 | 11040000 | 1440 | X | 17280000 | 17360000 |
| 690 | 3L | 11040000 | 11120000 | 1441 | X | 17360000 | 17440000 |
| 691 | 3L | 11120000 | 11200000 | 1442 | X | 17440000 | 17520000 |
| 692 | 3L | 11200000 | 11280000 | 1443 | X | 17520000 | 17600000 |
| 693 | 3L | 11280000 | 11360000 | 1444 | X | 17600000 | 17680000 |
| 694 | 3L | 11360000 | 11440000 | 1445 | X | 17680000 | 17760000 |
| 695 | 3L | 11440000 | 11520000 | 1446 | X | 17760000 | 17840000 |
| 696 | 3L | 11520000 | 11600000 | 1447 | X | 17840000 | 17920000 |
| 697 | 3L | 11600000 | 11680000 | 1448 | X | 17920000 | 18000000 |
| 698 | 3L | 11680000 | 11760000 | 1449 | X | 18000000 | 18080000 |
| 699 | 3L | 11760000 | 11840000 | 1450 | X | 18080000 | 18160000 |
| 700 | 3L | 11840000 | 11920000 | 1451 | X | 18160000 | 18240000 |
| 701 | 3L | 11920000 | 12000000 | 1452 | X | 18240000 | 18320000 |
| 702 | 3L | 12000000 | 12080000 | 1453 | X | 18320000 | 18400000 |
| 703 | 3L | 12080000 | 12160000 | 1454 | X | 18400000 | 18480000 |
| 704 | 3L | 12160000 | 12240000 | 1455 | X | 18480000 | 18560000 |
| 705 | 3L | 12240000 | 12320000 | 1456 | X | 18560000 | 18640000 |
| 706 | 3L | 12320000 | 12400000 | 1457 | X | 18640000 | 18720000 |
| 707 | 3L | 12400000 | 12480000 | 1458 | X | 18720000 | 18800000 |
| 708 | 3L | 12480000 | 12560000 | 1459 | X | 18800000 | 18880000 |
| 709 | 3L | 12560000 | 12640000 | 1460 | X | 18880000 | 18960000 |
| 710 | 3L | 12640000 | 12720000 | 1461 | X | 18960000 | 19040000 |
| 711 | 3L | 12720000 | 12800000 | 1462 | X | 19040000 | 19120000 |
| 712 | 3L | 12800000 | 12880000 | 1463 | X | 19120000 | 19200000 |
| 713 | 3L | 12880000 | 12960000 | 1464 | X | 19200000 | 19280000 |
| 714 | 3L | 12960000 | 13040000 | 1465 | X | 19280000 | 19360000 |
| 715 | 3L | 13040000 | 13120000 | 1466 | X | 19360000 | 19440000 |
| 716 | 3L | 13120000 | 13200000 | 1467 | X | 19440000 | 19520000 |
| 717 | 3L | 13200000 | 13280000 | 1468 | X | 19520000 | 19600000 |
| 718 | 3L | 13280000 | 13360000 | 1469 | X | 19600000 | 19680000 |
| 719 | 3L | 13360000 | 13440000 | 1470 | X | 19680000 | 19760000 |
| 720 | 3L | 13440000 | 13520000 | 1471 | X | 19760000 | 19840000 |
| 721 | 3L | 13520000 | 13600000 | 1472 | X | 19840000 | 19920000 |
| 722 | 3L | 13600000 | 13680000 | 1473 | X | 19920000 | 20000000 |
| 723 | 3L | 13680000 | 13760000 | 1474 | X | 20000000 | 20080000 |
| 724 | 3L | 13760000 | 13840000 | 1475 | X | 20080000 | 20160000 |
| 725 | 3L | 13840000 | 13920000 | 1476 | X | 20160000 | 20240000 |
| 726 | 3L | 13920000 | 14000000 | 1477 | X | 20240000 | 20320000 |
| 727 | 3L | 14000000 | 14080000 | 1478 | X | 20320000 | 20400000 |
| 728 | 3L | 14080000 | 14160000 | 1479 | X | 20400000 | 20480000 |
| 729 | 3L | 14160000 | 14240000 | 1480 | X | 20480000 | 20560000 |
| 730 | 3L | 14240000 | 14320000 | 1481 | X | 20560000 | 20640000 |
| 731 | 3L | 14320000 | 14400000 | 1482 | X | 20640000 | 20720000 |
| 732 | 3L | 14400000 | 14480000 | 1483 | X | 20720000 | 20800000 |
| 733 | 3L | 14480000 | 14560000 | 1484 | X | 20800000 | 20880000 |
| 734 | 3L | 14560000 | 14640000 | 1485 | X | 20880000 | 20960000 |
| 735 | 3L | 14640000 | 14720000 | 1486 | X | 20960000 | 21040000 |
| 736 | 3L | 14720000 | 14800000 | 1487 | X | 21040000 | 21120000 |
| 737 | 3L | 14800000 | 14880000 | 1488 | X | 21120000 | 21200000 |
| 738 | 3L | 14880000 | 14960000 | 1489 | X | 21200000 | 21280000 |
| 739 | 3L | 14960000 | 15040000 | 1490 | X | 21280000 | 21360000 |
| 740 | 3L | 15040000 | 15120000 | 1491 | X | 21360000 | 21440000 |
| 741 | 3L | 15120000 | 15200000 | 1492 | X | 21440000 | 21520000 |
| 742 | 3L | 15200000 | 15280000 | 1493 | X | 21520000 | 21600000 |
| 743 | 3L | 15280000 | 15360000 | 1494 | X | 21600000 | 21680000 |
| 744 | 3L | 15360000 | 15440000 | 1495 | X | 21760000 | 21840000 |
| 745 | 3L | 15440000 | 15520000 | 1496 | X | 21840000 | 21920000 |
| 746 | 3L | 15520000 | 15600000 | 1497 | X | 21920000 | 22000000 |
| 747 | 3L | 15600000 | 15680000 | 1498 | X | 22000000 | 22080000 |
| 748 | 3L | 15680000 | 15760000 | 1499 | X | 22080000 | 22160000 |
| 749 | 3L | 15760000 | 15840000 | 1500 | X | 22160000 | 22240000 |
| 750 | 3L | 15840000 | 15920000 | 1501 | X | 22240000 | 22320000 |
| 751 | 3L | 15920000 | 16000000 | 1502 | X | 22320000 | 22400000 |
|  |  |  |  | 1503 | X | 22400000 | 22480000 |
